# Polygenic risk for white matter hyperintensities is associated with early cerebrovascular events partly through hemodynamic measures in cognitively unimpaired middle-aged and older adults with low cardiovascular risk

**DOI:** 10.3389/fneur.2025.1667424

**Published:** 2026-01-05

**Authors:** Patricia Genius, Blanca Rodríguez-Fernández, Carolina Minguillon, Anna Brugulat-Serrat, Jordi Huguet, Manel Esteller, Carole H. Sudre, Marta Cortés Canteli, Catarina Tristão-Pereira, Inés García Lunar, Arcadi Navarro, Juan Domingo Gispert, Natalia Vilor-Tejedor

**Affiliations:** 1 Barcelonaβeta Brain Research Center (BBRC), Pasqual Maragall Foundation, Barcelona, Spain; 2 Hospital del Mar Medical Research Institute, Barcelona, Spain; 3 Centre for Genomic Regulation (CRG), Barcelona Institute of Science and Technology (BIST), Barcelona, Spain; 4 Doctoral School, PhD Programme in Bioinformatics, University of Vic–Central University of Catalonia (UVic-UCC), Vic, Spain; 5 Autonomous University of Barcelona, Barcelona, Spain; 6 Centro de Investigación Biomédica en Red de Fragilidad y Envejecimiento Saludable (CIBERFES), Instituto de Salud Carlos III, Madrid, Spain; 7 Global Brain Health Institute, University of California, San Francisco, San Francisco, CA, United States; 8 Josep Carreras Leukaemia Research Institute (IJC), Barcelona, Spain; 9 Institució Catalana de Recerca i Estudis Avançats (ICREA), Barcelona, Spain; 10 Department of Physiological Sciences, School of Medicine and Health Sciences, University of Barcelona (UB), Barcelona, Spain; 11 Centro de Investigación Biomédica en Red Cancer (CIBERONC), Madrid, Spain; 12 MRC Unit for Lifelong Health and Ageing, Department of Population Science and Experimental Medicine, University College London, London, United Kingdom; 13 Hawkes Institute, Department of Computer Science, University College London, London, United Kingdom; 14 Department of Neurodegenerative Disease, The Dementia Research Centre, UCL Queen Square Institute of Neurology, University College London, London, United Kingdom; 15 School of Biomedical Engineering and Imaging Sciences, King’s College London, London, United Kingdom; 16 Centro Nacional de Investigaciones Cardiovasculares (CNIC), Fuencarral-El Pardo, Madrid, Spain; 17 Centro de Neurociencias Cajal – Consejo Superior de Investigaciones Científicas (CSIC), Madrid, Spain; 18 Department of Cardiology, University Hospital La Moraleja, CIBER de Enfermedades Cardiovasculares (CIBERCV), Madrid, Spain; 19 Department of Experimental and Health Sciences, Institute of Evolutionary Biology (CSIC-UPF), Universitat Pompeu Fabra, Barcelona, Spain; 20 Institute of Evolutionary Biology (CSIC-UPF), Department of Experimental and Health Sciences, Universitat Pompeu Fabra, Barcelona, Spain; 21 Centro de Investigación Biomédica en Red Bioingeniería, Biomateriales y Nanomedicina, Instituto de Salud Carlos III, Madrid, Spain; 22 Department of Genetics, Radboud University Nijmegen Medical Center, Nijmegen, Netherlands; 23 Institute for Risk Assessment Sciences (IRAS), Department of Veterinary Medicine, Utrecht University, Utrecht, Netherlands

**Keywords:** cardiovascular risk, white matter hyperintensities, blood pressure measurement, polygenic risk score, cerebrovascular disease, dementia, risk factors

## Abstract

**Background:**

White matter hyperintensities (WMH) are a hallmark of cerebrovascular disease. They are often found in middle-aged individuals and are associated with a greater risk of stroke and vascular dementia. Although traditional cardiovascular risk factors are linked to WMH, some individuals with low vascular risk according to conventional scales still show WMH burden, suggesting an increased vulnerability. This study aimed to elucidate the biological mechanisms underlying the presence of WMH in cognitively unimpaired (CU) middle-aged and older individuals with low cardiovascular risk.

**Methods:**

We included 1,072 CU participants from the ALFA study with a low cardiovascular risk profile for late-life dementia (CAIDE-I score ≤ 9). We defined a multi-stage exploratory study design to reveal the potential biological pathways driving WMH in the study sample. First, we estimated the genetic predisposition to WMH using polygenic scoring (PRS_WMH_) and used this score as a predictor of: (a) WMHV as a subclinical quantitative measure of global and regional WMH burden and (b) pathological WMH levels (pathological: Fazekas score ≥ 2), as a qualitative measure of clinically relevant WMH. Covariate-adjusted Spearman’s rank correlation tests evaluated the association between the PRS_WMH_ and regional and global WMH volumes (WMHV), while a logistic regression model was performed to explore the association with pathological WMH. Second, group-stratified partial correlations (CAIDE-specific factors) were explored to identify homogeneous groups with persistent genetic associations with WMH, beyond the presence of cardiovascular risk factors. Third, an enrichment analysis of the PRS-annotated genes unveiled the biological mechanisms leading to WMH burden. Finally, based on the enrichment analysis, we examined the role of cardiometabolic traits as biomarkers of WMHV.

**Results:**

Genetic predisposition to WMH was associated with larger global and regional WMHV after adjusting for age and sex, specifically in frontal areas. In this group, larger WMHV were associated with poorer executive function. Group-stratified analyses showed significant correlations particularly among older participants, those with hypercholesterolemia and those with lower educational attainment. Gene-set enrichment involved vascular, neuronal and cellular pathways, and blood pressure measurements partially mediated the association between the genetic risk for WMH and the actual WMHV.

**Interpretation:**

These findings support a polygenic contribution to cerebrovascular burden and nominate cardiac function as a biological link along the heart-brain axis. While the PRS_WMH_ is not yet clinically actionable, our results propose and prioritize hemodynamic monitoring as an early, testable intervention in genetically susceptible individuals, to help prevent cerebrovascular damage and downstream cognitive impairment in healthy participants with low cardiovascular risk profile.

## 1 Introduction

White matter hyperintensities (WMH) are a well-established markers of cerebral small vessel disease (CSVD). Different pathophysiological pathways have been proposed to explain their appearance (1), mainly categorized into vascular (e.g., due to ischemia/hypoperfusion) and non-vascular (e.g., due to gliosis, axonal loss) origin. Age and hypertension are the main risk factors for WMH (2, 3), but other cardiovascular risk factors (CVRF) (4), such as cholesterol (5) and obesity (6) also play a significant role.

WMH contribute to cognitive impairment (CI) and neurodegeneration, and have a major clinical impact in the general population, increasing the risk of stroke, cognitive decline and dementia (7, 8). In longitudinal studies involving cognitively healthy elderly individuals (9), larger WMH have been associated with worse cognitive performance, even when controlling for the effect of CVRF (10). Remarkably, authors identified a pathological threshold for WMH burden (11), which, independently of age and other risk factors, increased the risk of deterioration and functional dependence among participants without baseline complaints (9). These findings suggest that, beyond the presence of WMH as a common feature of aging, there is a clinically relevant level of WMH severity that predicts global functional decline, and the study of cardiovascular risk factors may not be sufficient to mechanisms underlying the presence of WMH.

Given these implications, more research is needed to understand the mechanisms throughout WMH impair cognition and brain health. WMH are a frequent comorbidity in Alzheimer’s disease (AD) (1, 12–14). Recent studies have shown increased WMH burden in AD patients when compared to mild cognitive impaired (MCI) individuals and cognitively unimpaired (CU) older adults (15). Nonetheless, dementia in older adults typically lies on a continuum between AD and vascular dementia (VaD) (16–18), with frequent co-pathologies and overlapping phenotypes. At preclinical stages of the AD (19), WMH and AD pathology accumulate and interact in a spatio-temporal way across the whole disease continuum (20). Recent findings suggest that, in cognitively normal and MCI older adults, vascular burden is primarily related to larger frontal WMHV, whereas AD core CSF biomarkers are mainly linked to temporal and parietal WMH (21). These patterns imply that, at preclinical stages of AD, WMH burden may reflect both vascular and AD-specific processes. Therefore, targeting modifiable vascular pathways in pre-symptomatic individuals with abnormal CSF profiles could help to reduce cerebrovascular burden, improve brain health and open a new window for targeted preventive strategies in AD participants, for which available treatments still present specific challenges, such as stringent eligibility criteria and safety monitoring of the participants (van (22)).

To explore additional therapeutic targets, the evaluation of WMH should extend beyond modifiable risk factors alone. WMH are a strong heritable phenotype (~40 to 70%) (23) and share common genetic risk factors with AD and other dementias (24, 25). For instance, several studies have found significant associations between the *APOLIPOPROTEIN E* gene (*APOE*) *ε4* allele and larger WMHV in cognitively unimpaired (CU) individuals (4, 26, 27). However, contrary to the previous findings, others have suggested that there may be no association or even a reverse association between the *APOE-ε4* allele and white matter lesions in AD patients (28). These results should be cautiously interpreted, as heterogeneous vascular risk profiles may play a role in developing WMH in the absence of the *APOE-ε4* allele in AD. Beyond *APOE*, genome-wide association studies (GWAS) have identified novel genetic variants associated with WMH burden and microstructure (29, 30), which suggested a polygenic architecture of WMH (31). Interestingly, recent studies have found a positive association between higher polygenic risk of WMH and increased risk of both CSVD and AD (32). Genetic architecture of WMH has been linked to genetic loci that are implicated in inflammatory and glial proliferative pathways, variants mapped to genes implicated in AD, intracerebral hemorrhage, neuroinflammatory diseases and glioma, as well as blood pressure regulation (30). Specifically, recent studies found that genes associated with WMHV closer to the ventricles (i.e., *periventricular WMH*), which are highly linked to elevated systolic blood pressure (SBP), were mostly involved in vascular function and ischemic stroke (33). On the other hand, genes associated with more peripheral WMHV (i.e., *deep WMH*), which are highly linked to arterial hypertension (both SBP and diastolic blood pressure (DBP)), were mostly involved in vascular, astrocyte and neuronal function. These findings suggest separate causes for periventricular and deep WMH, which they both have been associated with cognitive decline and obesity and gait dysfunction.

However, less is known about the causes and consequences of WMH lesions in asymptomatic cognitively healthy individuals. In our ALzheimer’s and FAmilies (ALFA) cohort (34), characterized by individuals healthier than an age-matched cohort, but with a high proportion of AD patients’ offsprings, we found a high prevalence of incidental findings in MRI, remarkably for severe WMH being prevalent in 8% of the sample (35). In a study including ALFA participants, we also found that larger WMHV were associated with worse cognitive outcomes (36). Nonetheless, the mechanisms contributing to cerebrovascular disease in these healthy asymptomatic populations still remain unclear.

Given the lack of research, the urgency to identify additional therapeutic targets to improve cognitive outcomes, and the suitability of the ALFA cohort to examine cerebrovascular imaging features in the context of dementia prevention, the present work adopts a multi-stage exploratory study design to examine the biological mechanisms underlying the presence of WMH in CU middle-aged and older individuals of the ALFA cohort (34), at low cardiovascular risk for late-life dementia but elevated genetic risk for AD. By selecting participants with low cardiovascular risk, based on the Cardiovascular Risk Factors, Aging, and Incidence of Dementia I (CAIDE-I) risk score (CAIDE-I score ≤ 9), but enriched with AD-related genetic risk factors, we obtain a suitable cohort for studying the genetic influences of WMH, minimizing the impact of known modifiable risk factors and increasing the likelihood of identifying potential pathways related to cerebrovascular burden in individuals at risk of AD. First, we assessed whether the genetic predisposition to WMH, assessed through the polygenic risk score of WMH (PRS_WMH_), was a proxy for larger global and regional WMHV as well as a proxy of clinically defined WMH pathological levels in the study sample. Second, we examined in which specific groups of individuals, the PRS_WMH_ was persistently associated with WMHV even in presence of specific CVRF. Third, we explored the biological mechanisms associated with WMH by performing an enrichment analysis of the genes annotated to the genetic variants included in the PRS_WMH_. Lastly, based on the enriched biological pathways observed in the enrichment analysis, we investigated the role of lipids and blood pressure (BP) measurements as potential biomarkers of WMH.

## 2 Methods

### 2.1 Study population

Participants of this study are part of the ALFA study composed of 2,743 CU european individuals aged between 45 to 75 years old, most of them offspring of AD patients. Participants had a Clinical Dementia Rating score equal to 0 and were excluded if they had major psychiatric disorders or other diseases that could affect cognition, neurological disorders, brain injury (i.e., head trauma with parenchymal lesion or extra axial macroscopic large vessel ischemic stroke or hemorrhagic stroke) that could affect cognition, or family history of AD with suspected autosomal dominant pattern. Although individuals with recent neurological disorders and brain injury were excluded, cardiovascular and endocrino-metabolic comorbidities were self-reported by 30 and 42% of the participants, respectively, being both current hypertension and dyslipidemia the most prevalent. However, 80% of individuals had a BMI ≤ 30 and 74% a measured systolic blood pressure (SBP) ≤140, indicating a lower risk of developing cardiovascular disease. For a full detailed description of the ALFA study and its inclusion and exclusion criteria see (34).

### 2.2 Standard protocol approvals, registrations, and patient consents

The ALFA study protocol and informed consent were approved by an independent Ethics Committee “Parc de Salut Mar,” Barcelona, and is registered at Clinicaltrials.gov (Identifier: NCT01835717).

### 2.3 Sociodemographic, lifestyle and clinical factors

Sociodemographic, lifestyle and clinical factors were collected. Participants were considered to have hypertension when (i) the measured systolic blood pressure was above 140 mmHg, (ii) they were under an antihypertensive treatment or (iii) it was self-reported. Body mass index (BMI) was derived from the height and weight and individuals were classified as underweight (BMI < 18.5), normal weight (18.5 ≤ BMI ≤ 24.9), overweight (25 ≤ BMI ≤ 29.9), and obese (BMI ≤ 30). Physical activity was measured using the Spanish short version of Minnesota Leisure Time Physical Activity Questionnaire (37). Since total cholesterol levels were not available for all the participants, individuals were classified as hypercholesterolemic when they self-reported either the presence of dyslipidemia or the use of medication (lipid modifying therapies (e.g., simvastatina, atorvastatina, pitavastatina). Educational attainment was determined by assessing the number of years of formal education completed.

### 2.4 Cardiovascular risk for late-life dementia: CAIDE score

Specific sociodemographic, lifestyle and clinical risk factors were included in the CAIDE-I risk score predicting 20-year risk for late-life dementia (Supplementary Figure 1). In the standard protocol for the CAIDE score computation (38), variables are dichotomized or categorized in tertiles based on standard cutoffs. In ALFA, participants were assigned a score depending on the group they belonged to for each one of the vascular risk factors (4); Supplementary Table 1). In the current study, individuals scoring below 10 (CAIDE-I score ≤ 9) were classified as low-risk for developing dementia late in life and were included in the final sample of the study. Individuals with a CAIDE-I score >9 were excluded from the study (Supplementary Figure 2).

### 2.5 Cognitive measures in the sample

A total of 758 individuals from the study sample (*n =* 1,072) had available information on cognitive function. Verbal episodic memory was evaluated through the Spanish validated version A of the Free and Cued Selective Reminding Test (FCSRT) (39). The FCSRT consists of the learning and retention of a list of 16 semantically unrelated words through a controlled learning process that uses semantic encoding (40). Moreover, executive functioning was evaluated using the Wechsler Adult Intelligence Scale-WAIS-IV (41). We calculated composites for episodic memory and executive functioning domains by averaging the normalized scores of the tests included in the FCSRT and WAIS-IV, respectively. More details can be found in Supplementary Results.

### 2.6 Genetic data acquisition, quality control and imputation

DNA was obtained from blood samples through a salting out protocol. Genotyping was performed with the Illumina Infinium Neuro Consortium (NeuroChip) Array (build GRCh37/hg19). The NeuroChip array is a custom genotyping Illumina platform enriched for neurological and neurodegenerative disease-related variants (170 K). Quality control was performed using PLINK software. Imputation was performed using the Michigan Imputation Server with the haplotype Reference Consortium Panel (HRC r1.1 2016) (42) following default parameters and established guidelines. A full description of the genotyping, quality control and imputation procedures is available elsewhere (43).

### 2.7 Polygenicity of white matter hyperintensities

Polygenicity of WMH was calculated using the PRSice version 2 tool (44). Summary statistics from a recent GWAS for WMH (29) were obtained to compute the PRS_WMH_ (Supplementary Table 2). We explored the joint effect of single nucleotide polymorphisms (SNPs) displaying a significance of 5·10^−6^, to guarantee enough variability in the sample while maintaining the most significant loci related to WMH. For the PRS computation, we first applied the clumping method, which consisted on retaining the single nucleotide polymorphisms (SNPs) with the smallest *p*-value in each 250 kb window and removing the SNPs that were in linkage disequilibrium (r^2^ > 0.1). After removing the highly correlated SNPs, the PRS_WMH_ was computed by adding up the alleles carried by participants, weighted by the SNP allele effect size from the GWAS, and normalizing by the total number of alleles (Supplementary Tables 2, 3). A total number of 25 SNPs remained in the computation of the PRS, out of 708 with a significance below 5 × 10^−6^. To further explore the contribution of genetic factors beyond *APOE,* the same PRS_WMH_ was re-calculated excluding the *APOE* region (PRS_WMHnoAPOE_) (chr19:45,409,011-45,412,650; GRCh37/hg19).

### 2.8 Magnetic resonance imaging acquisition and WMH volume quantification

MRIs were acquired on a Philips Ingenia CX 3 T MRI scanner. The MRI protocol included a high resolution a 3D T1-weighted sequence (voxel size = 0.75 × 0.75 × 0.75 mm, TR/TE = 9.9/4.6 ms, Flip Angle = 8°) and a 3D fluid attenuation inversion recovery (T2-FLAIR) scan (voxel size = 1 × 1 × 1 mm, TR/TE/IR = 5000/312/1700 ms). All scans were visually assessed for quality and incidental findings by a trained neuroradiologist (35). A Bayesian algorithm was used to quantify WMHV (mm^3^) from T1-weighted and T2-FLAIR MRI scans (45). Regional WMHV were quantified at five different distances from the ventricles and for six different regions in both the right and left hemispheres. We worked with regional volumes in the frontal, temporal, parietal and occipital lobes, as well as in the basal ganglia. We did not include the infratentorial region. To reduce dimensionality, regional WMH were categorized into three subtypes based on the distance from the ventricles (periventricular, deep and juxtacortical). Volumes were averaged across hemispheres to produce a single regional measure with equal hemisphere weighting, as pronounced WMH asymmetry is typically associated with clinical populations (46). Volumes were adjusted for total intracranial volume (TIV). Similarly, global WMHV was expressed as the average volume between right and left hemispheres and normalized by TIV. All MRIs were visually assessed by a trained neuroradiologist who was blinded to the *APOE* genotype of the participants. Images were rated using modifications of the Fazekas Scale (11), which separately categorizes WMH load in a pathological level based on a scale ranging from 0 to 3 (0, none or a single punctate WMH lesion; 1, multiple punctate lesions; 2, beginning confluency of lesions (bridging); and 3, large confluent lesions). WMH pathological levels were defined based on the cut-off of 2 (Fazekas score <2: non-pathological WMH, Fazekas score ≥ 2: pathological WMH) (9, 47, 48).

### 2.9 Available cardiometabolic traits

#### 2.9.1 Lipid levels in serum

Biochemical analyses of lipids were performed on serum samples by the Reference Laboratory of Catalunya. Measurements for triglycerides (TG), total cholesterol (Tchol), low density lipoprotein (LDL) and high density lipoprotein (HDL) levels were available for a reduced study sample (*N =* 237, 22% of the total sample). LDL levels were calculated through the Friedewald formula (49). Lipid levels were categorized as pathologic or non-pathologic based on standard reference ranges for high blood cholesterol treatment in adults (50). Pathological levels were considered when Tchol ≥ 240 mg/dL, LDL ≥ 160 mg/dL, TG ≥ 200 mg/dL and HDL < 40 mg/dL (LDL = Tchol-HDL + TG/5). To account for potential variability in serum lipid measurements due to biological, dietary or pharmacological influences, a binary classification of dyslipidemia was applied. Participants were classified as dyslipidemic if they either self-reported hypercholesterolemia, used lipid-modifying treatments or displayed pathological lipid levels. Conversely, participants were classified as non-dyslipidemic if the previous conditions were not met. A total of 173 were classified as displaying dyslipidemia for HDL (73%), 155 for LDL (66%), 165 for Tchol (70%) and finally, 158 for TG (66.6%). More details about grou p composition can be found in Supplementary Results.

#### 2.9.2 Blood pressure measurements

SBP, DBP and heart rate (HR; cardiac frequency) were available for a total of 754 individuals (70% of the study population). These measurements reflected systemic arterial pressure measured at rest. SBP measures the BP when the heart is beating, while DBP refers to the BP when the heart is resting. Both indices were measured per mmHg. HR was reported as the number of heart beats per minute. Measures were obtained at two different times in the same visit. We worked with the averaged value. We derived additional metrics from these measurements: Pulse pressure (PP = SBP - DBP), Mean arterial pressure (MAP = DBP + 1/3(SBP – DBP)), and Rate-pressure product (RPP = HR x SBP). RPP is an indirect index of myocardial oxygen consumption that predicts cardiac function and mortality in patients with cardiovascular disease (51). MAP refers to the average arterial pressure throughout one cardiac cycle, systole, and diastole, and it is influenced by cardiac output and systemic vascular resistance. MAP is the closest surrogate for cerebral perfusion pressure over the cardiac cycle. Finally, PP refers to the pulsatile component of arterial pressure and correlates with arterial stiffness and excessive transmission of pressure. Both steady measurements (SBP, DBP, MAP) and pulsatile components (HR, PP, RPP) independently predict mortality (52). More details about BP measurements distribution can be found in Supplementary Results.

### 2.10 Statistical analysis

A descriptive analysis of the study sample was performed using chi-square tests for categorical variables and parametric (*t*-test) and non-parametric tests (Wilcox test) for continuous normally and non-normally distributed variables, as appropriate. Moreover, non-parametric tests (Mann–Whitney and Kruskal Wallis) were performed to explore differences in the median value for the PRS_WMH_ and WMHV between risk groups for each CAIDE-I component. An extended descriptive characterization of the sample included the cross-sectional evaluation of the relationship between WMHV and cognitive functioning. Analyses were not included as a part of the main design, but explained and presented in Supplementary Results.

A multi-stage exploratory design was defined to explore the biological mechanisms linked to WMH, including: (1) validation of the PRS_WMH_ as a proxy of larger WMHV and clinically defined pathological WMHV, (2) identification of individuals for whom genetics were still predicting larger WMHV even in presence of cardiovascular risk factors, (3) examination of biological pathways leading to WMH and (4) exploration of potential biomarkers of WMH.

The first step aimed to test whether the polygenic risk score of WMH was a proxy of larger WMHV and clinically defined pathological WMH in the sample. When working with global and regional WMHV (quantitative outcome) we applied covariate-adjusted Spearman’s rank correlation test (partial Spearman) (53) to examine the association with larger WMHV, both globally and regionally. This non-parametric approach was selected because of the highly asymmetric and skewed distribution of WMHV and log-transformed WMHV, as well as the non-linear association between the PRS-WMH and WMHV. Details can be found in Supplementary Methods. Partial correlations were adjusted for age and sex by computing Spearman correlations between the probability-scaled residuals (54) of PRS_WMH_ and WMHV regressed on age and sex. Sensitivity analyses were performed adjusting the models for the CAIDE-I score and hypertension status. To assess the specific impact of *APOE-𝜀4* carriership, we conducted the same partial correlations between the PRS-WMH and WMHV using the PRS_WMH_ that excluded the *APOE* region (PRS_WMHnoAPOE_) while additionally adjusting for *APOE-𝜀4* carriership. When working with the clinical definition of pathological WMH levels, we performed a logistic regression model to explore the association with the genetic predisposition to WMH adjusting for age, sex and hypertension.

The second step consisted of identifying the profile of the participants for whom genetics were persistently related to larger WMHV even in presence of WMH-related cardiovascular risk factors (CAIDE-I components). For each CAIDE-I component, we classified individuals into more homogeneous groups based on the absence or presence of the risk factors. Individuals who belonged to the “underweight” group (*n =* 3) were excluded and a binary variable was created to classify individuals as obese and non-obese (cut-off point BMI ≥ 30). Individuals were classified as being active ( ≥ 150 min) or inactive (<150 min) based on the time of physical activity per week. For quantitative variables, the classification was based on the distribution of the values in the sample. Based on the distribution of the age, participants were divided into three groups: (i) 45–54 years old group, (ii) 55–64 years old group and (iii) 65–77 years old group. Similarly, individuals were classified into low (0–6 years), intermediate (7–9 years) and high ( ≥ 10 years) education groups. First, a chi-square test was used to compare the cardiovascular risk profile of individuals classified into different groups within each cardiovascular risk factor. Second, stratified partial Spearman’s rank correlation tests were performed to assess the correlation between the PRS_WMH_ and global WMHV in each group for the seven CAIDE-I components. Models were adjusted for age and sex when required.

The third step aimed to reveal the biology behind the PRS_WMH_. Therefore, genetic variants associated with WMH (*p* < 5 × 10^−6^) were annotated to their nearest genes (distance of 10 kb) using *biomaRt* package in R as well as the *snpXplorer* algorithm, which allowed for a deeper characterization and functional interpretation of the effects of genetic variants (55). More details can be found in Supplementary Methods. An enrichment analysis was conducted to identify the primary biological pathways (Gene Ontology; GO terms) linked with the genes that confer higher risk of WMH by using *clusterProfiler 4.6* package (56) in R. One-sided version of Fisher’s exact test was used to determine whether known biological functions were overrepresented in the gene list and to calculate the probability of observing a set of genes in a particular biological pathway by chance. Enrichment analysis was performed based on the list of SNP-annotated genes pre- and post-clumping. Results are displayed for the post-clumped SNPs list, which is cohort-specific. Nonetheless, a more detailed exploration of the biological mechanisms linked to WMH, invariant across cohorts and non-influenced by cohort-specific aspects, can be found in Supplementary Methods.

Finally, based on the enrichment analysis results, we planned to examine potential biomarkers of WMH, mainly focusing on two main mechanistic pathways underlying the polygenicity of WMHV: (a) lipid-related mechanisms, due to the observed results in the non-cohort specific enrichment analysis, and (b) vascular-related mechanisms, due to the observed results in the cohort-specific enrichment. We first proposed (a) lipid levels in serum as potential biomarkers of WMHV. Nonetheless, only a subset of individuals had available measurements (*N =* 237, 22% of the total sample). This loss in sample size and, in consequence, in statistical power, complicated the possibility to extend the analyses to further explore a potential mediation role between the genetic architecture of WMH and WMHV. Results and potential sources of bias were described in Supplementary Results. Secondly, we proposed (b) a set of blood pressure measures as potential biomarkers of WMHV. A total of 754 individuals (70%) had available information for SBP, DBP and HR, which facilitated further explorations. Partial correlations were used to explore the association between the hemodynamic variables and WMHV, after adjusting for age and sex. Sensitivity analyses included hypertension as a covariate, to assess the direct effect of blood pressure measurements on WMHV. We also explored the association between the PRS of WMH and blood pressure measures, adjusting for age and sex. Given the significant associations observed between blood pressure measures, WMHV and the PRS_WMH_, we investigated whether these hemodynamic measures mediate the relationship between the PRS_WMH_ and both global and regional WMHV. WMHV values were transformed using the *bestNormalize* R package (57), and causal mediation effects were estimated with the *mediation* R package All the analyses were performed using the R software version 4.2.2.

## 3 Results

### 3.1 Characterization of the study sample: demographics, WMH profile and cognitive vulnerability to WMHV

The study sample included 1,072 individuals (Supplementary Figure 2) at low risk to develop dementia late in life, where 64.3% (*N =* 689) were women. The median age of the sample was 59 years [IQR 53; 64] and the median value for total years of education was 14 [IQR 11; 17] (Table 1). We observed significant differences when comparing between non-pathological and pathological groups based on WMH severity. In the group displaying pathological WMH, individuals were older (*p <* 0.001), had higher risk to develop dementia late in life based on the CAIDE score (*p <* 0.001), and there was a higher percentage of hypertensive (*p <* 0.001) individuals. They also displayed higher TIV (*p =* 0.001) and global WMHV (*p <* 0.001), which increased with higher Fazekas scores (Supplementary Figure 3). Non-significant differences were found in the genetic predisposition to WMH between pathological WMH groups. Nonetheless, when comparing between individuals with different cardiovascular risk profiles, we observed that genetic predisposition to WMH was higher in obese, hypercholesterolemic and hypertensive individuals compared to the non-risk counterparts within each group (Supplementary Figure 4). Global WMHV increased across all age ranges and was higher in hypertensive individuals and women when compared to their counterparts (Supplementary Figure 5). Additionally, in the individuals with low cardiovascular risk and available cognitive measurements (*n =* 758), WMHV were associated with poorer executive function independently of aging- and AD-related cortical thickness, hypertension, neurodegeneration and other main risk factors. Detailed results are provided in Supplementary Results.

**Table 1:** Demographic and cardiovascular characteristics of the study sample, stratifying by pathological WMH levels.

Sociodemographic and clinical factors	Sample of the study *N =* 1,072	Non-pathological WMH (Fazekas <2, *N =* 980)	Pathological WMH (Fazekas ≥2, *N =* 83)	*p*-value
Sex				0.108
Men	383 (35.728%)	344 (35.102%)	37 (44.578%)	
Women	689 (64.272%)	636 (64.898%)	46 (55.422%)	
Age	59.000 [53.000; 64.000]	58.000 [53.000; 63.000]	64.000 [60.000; 67.000]	**<0.001**
Categorical Age				**<0.001**
47–55 yo	377 (35.168%)	365 (37.245%)	10 (12.048%)	
56–64 yo	469 (43.750%)	427 (43.571%)	36 (43.373%)	
65–77 yo	226 (21.082%)	188 (19.184%)	37 (44.578%)	
Years of Education	14.000 [11.000; 17.000]	14.000 [11.000; 17.000]	14.000 [11.000; 17.000]	0.539
Categorical Education				0.986
5–10 yr	225 (20.989%)	206 (21.020%)	18 (21.687%)	
11–14 yr	317 (29.571%)	290 (29.592%)	24 (28.916%)	
15–18 yr	530 (49.440%)	484 (49.388%)	41 (49.398%)	
*APOE ε*4 carriership				0.211
Allele ε4 Carriers	415 (38.713%)	374 (38.163%)	38 (45.783%)	
Allele ε4 Non-Carriers	657 (61.287%)	606 (61.837%)	45 (54.217%)	
CAIDE-I score	5.000 [4.000; 7.000]	5.000 [4.000; 7.000]	7.000 [5.000; 8.000]	**<0.001**
Hypertension				**<0.001**
Hypertensive	163 (15.205%)	136 (13.878%)	26 (31.325%)	
Non-Hypertensive	909 (84.795%)	844 (86.122%)	57 (68.675%)	
Body Mass Index				0.051
Underweight	3 (0.280%)	3 (0.306%)	0 (0.000%)	
Normal Weight	443 (41.325%)	416 (42.449%)	23 (27.711%)	
Overweight	471 (43.937%)	419 (42.755%)	47 (56.627%)	
Obese	155 (14.459%)	142 (14.490%)	13 (15.663%)	
Hypercholesterolemia				0.132
Hypercholesterolemic	270 (25.187%)	250 (25.510%)	28 (33.735%)	
Non-Hypercholesterolemic	802 (74.813%)	730 (74.490%)	55 (66.265%)	
Minutes of physical activity (per month)	2011.875 [1012.875; 3514.375]	1998.438 [983.250; 3531.656]	2452.500 [1139.375; 3413.438]	0.400
Physical activity				0.515
Active (≥150 min/week)	1,037 (96.735%)	946 (96.531%)	82 (98.795%)	
Inactive (<150 min/week)	35 (3.265%)	34 (3.469%)	1 (1.205%)	
PRS-WMH	−0.002 [−0.005; <0.001]	−0.002 [−0.005; <0.001]	−0.001 [−0.004; 0.001]	0.168
Total intracranial volume	1441538.539 [1332020.471; 1561860.173]	1438698.539 [1329745.167; 1554750.589]	1517769.830 [1379781.759; 1622791.098]	**0.001**
WMH volume	0.001 [0.001; 0.002]	0.001 [0.001; 0.002]	0.006 [0.004; 0.009]	**<0.001**
Fazekas scale				**<0.001**
NA	9 (0.840%)			
0	343 (31.996%)	343 (35.000%)	0 (0.000%)	
1	637 (59.421%)	637 (65.000%)	0 (0.000%)	
2	73 (6.81%)	0 (0.000%)	73 (87.952%)	
3	10 (0.933%)	0 (0.000%)	10 (12.048%)	
Blood pressure and cardiac indicators (*n =* 746)				
*SBP*	125 [114; 138]	124 [113; 138]	138 [125; 147]	**<0.001**
*DBP*	79.0 [72.0; 85.4]	78.8 [72.0; 85.0]	84.2 [77.0; 91.0]	**0.001**
*HR*	68.5 [61.6; 75.5]	68.5 [61.5; 75.5]	69.5 [63.0; 78.9]	0.222
*MAP*	94.7 [87.0; 102]	94.2 [86.7; 101]	100 [94.3; 108]	**<0.001**
*RPP*	8,614 [7,437; 9,833]	8,539 [7,400; 9,766]	9,616 [8,351; 10,793]	**<0.001**
*PP*	46.5 [37.5; 56.0]	46.0 [37.4; 55.0]	53.5 [43.0; 65.0]	**<0.001**
Time between clinical evaluation and MRI scan	3.58 [3.11; 4.33]	3.595 [3.123; 4.363]	3.422 [3.089; 3.970]	0.058

TIV, Total Intracranial Volume; MRI, Magnetic Resonance Imaging; WMH, White Matter Hyperintensities; yo, years old; yr, years; SBP, Systolic blood pressure; DBP, Diastolic blood pressure; HR, Heart rate; MAP, Mean arterial pressure; RPP, Rate-pressure product; PP, Pulse pressure. Median and interquartile range were reported for continuous non-normally distributed variables. Counts and percentage were reported for categorical variables. TIV was defined as mm^3^. WMH volume was adjusted for TIV and expressed for each 100 mm^3^. There were 9 missing values for the Fazekas scale score. *p*-values below the established significance level (.05) are highlighted in bold.

### 3.2 The polygenic risk score of WMH, beyond APOE effect, is a proxy of larger WMHV in individuals at low cardiovascular risk for late-life dementia, although it is not indicative of pathological WMH levels

**Figure 1:**
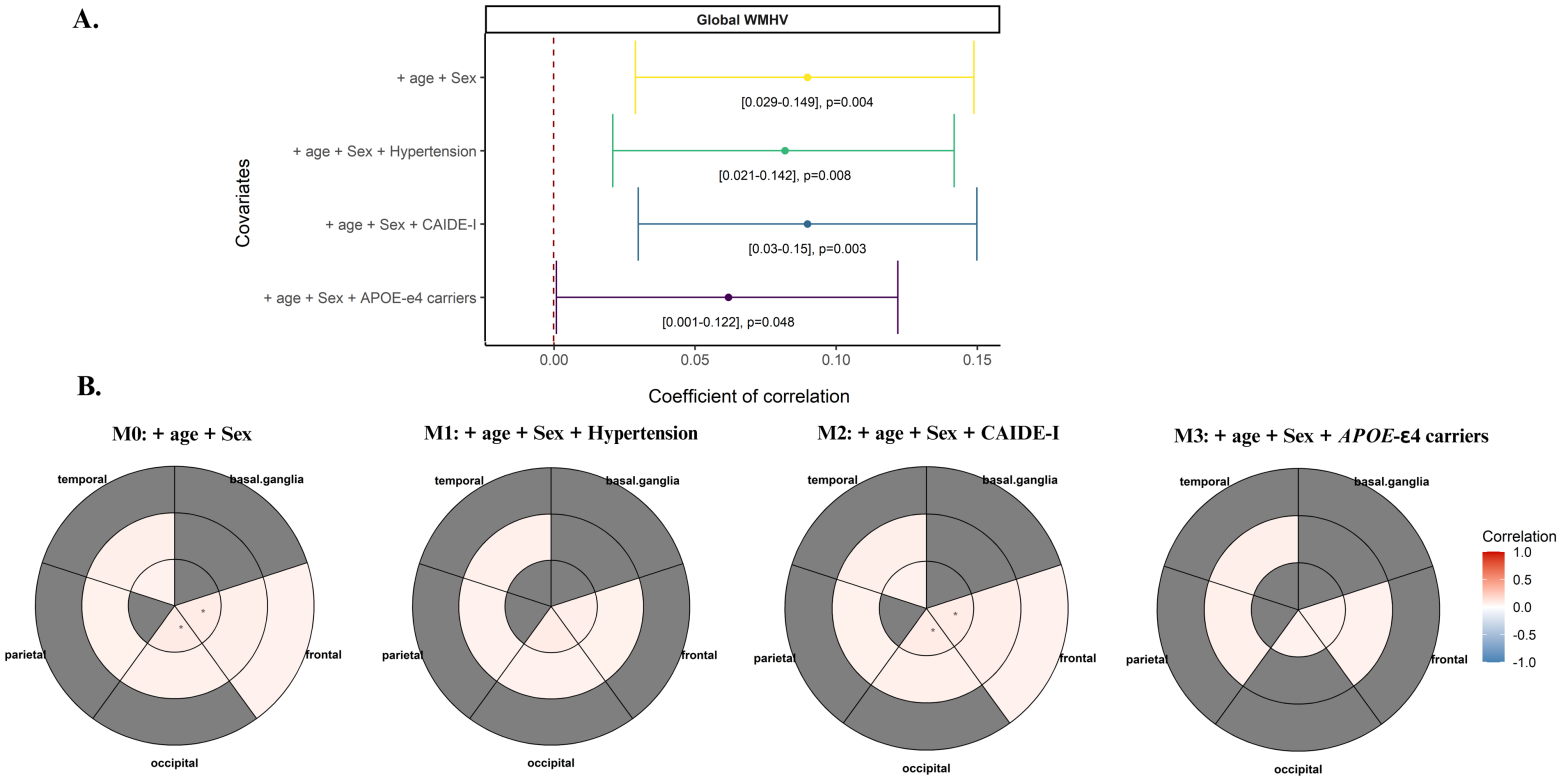
Covariate adjusted Spearman’s rank correlation test assessing the association between both **(A)** global and **(B)** regional WMHV and the genetic predisposition to WMH. The main model included age and sex as covariates. Sensitivity analyses additionally included hypertension status, the CAIDE-I score and APOE-E4 carriership (PRSWMHnoAPOE as predictor). **(A)** A forest plot was used to display the results of the partial correlation between the PRS of WMH and global WMHV. Confidence intervals and *p*-values were reported. **(B)** Bullseye plots were used to indicate the correlation between the PRS of WMH and regional WMHV. Concentric circles indicate the distance of the lesion, from internal layers referring to periventricular WMHV to more external layers referring to juxtacortical WMHV. Significant correlations (nominal *p* < 0.05) were indicated by colouring the cells in the bullseye plot. Gray areas indicate non-significant correlations. The asterisk indicates FDR <. 05.

Partial correlations showed that genetic predisposition to WMH was positively associated with larger global WMHV, even after adjusting for the effect of age and sex (*ρ* = 0.090 [0.029, 0.149], *p =* 0.004; Figure 1; Table 2A). Sensitivity analyses showed that results were still significant after adjusting the model for hypertension status (*ρ* = 0.082 [0.021, 0.142], *p =* 0.008), CAIDE-I score (*ρ* = 0.090 [0.030, 0.150], *p* = 0.003) and *APOE-𝜀4* carriership (*ρ* = 0.062 [0.001, 0.122], *p* = 0.048; Figure 1A; Table 2). Regarding regional WMH, the PRS_WMH_ was associated with larger periventricular WMHV in frontal (*ρ* = 0.088 [0.028, 0.147], *p* = 0.004), occipital (*ρ* = 0.093 [0.034, 0.152], *p* = 0.002) and temporal (*ρ* = 0.062 [0.092, 0.122], *p* = 0.043) areas, larger WMHV in all deep areas except basal ganglia, and finally juxtacortical frontal (*ρ* = 0.065 [0.006, 0.125], *p* = 0.032) areas (Figure 1B; Table 2). Only the association between the PRS of WMH and periventricular WMHV in both the frontal and occipital lobes remained significant after FDR correction (Table 2). Results from the logistic regression model did not show a significant association between higher genetic predisposition to WMH and pathological WMH levels (*p* = 0.471; Supplementary Table 4). The odds of displaying pathological WMH levels were higher with age (OR = 2.028, *p <* 0.001) and positive history of hypertension (OR = 1.863, *p =* 0.02; Supplementary Table 4).

**Table 2:** Results for the covariate-adjusted Spearman’s rank correlation test between the PRS of WMH and both WMHV.

Model	*ρ*	*p*-value	Low CI	High CI
Global WMHV				
PRS-WMH|WMH volume~ age_scan + Sex	0.090	**0.004**	0.029	0.149
PRS-WMH|WMH volume~ age_scan + Sex + Hypertension	0.082	**0.008**	0.021	0.142
PRS-WMH|WMH volume~ age_scan + Sex + CAIDE-I	0.090	**0.003**	0.030	0.150
PRS-WMH_noAPOE_ | WMH volume ~age_scan+Sex+APOE-𝜀4 carriership	0.062	**0.048**	0.001	0.122

Regional WMHV	*ρ*	*p*-value	Low CI	High CI	Adjusted *p*-value
Model 0					
PRS-WMH~periventricular basal.ganglia~ age_scan + Sex	0.030	0.315	−0.029	0.090	0.363
PRS-WMH~deep basal.ganglia~ age_scan + Sex	0.031	0.302	−0.028	0.091	0.363
PRS-WMH~juxtacortical basal.ganglia~ age_scan + Sex	0.021	0.489	−0.038	0.079	0.524
PRS-WMH~periventricular frontal~ age_scan + Sex	0.088	**0.004**	0.028	0.147	**0.030**
PRS-WMH~periventricular parietal~ age_scan + Sex	0.056	0.072	−0.005	0.116	0.108
PRS-WMH~periventricular occipital~ age_scan + Sex	0.093	**0.002**	0.034	0.152	**0.030**
PRS-WMH~periventricular temporal~ age_scan + Sex	0.062	**0.043**	0.002	0.122	0.081
PRS-WMH~deep frontal~ age_scan + Sex	0.077	**0.011**	0.017	0.135	0.055
PRS-WMH~deep parietal~ age_scan + Sex	0.068	**0.026**	0.008	0.128	0.065
PRS-WMH~deep occipital~ age_scan + Sex	0.070	**0.023**	0.009	0.130	0.065
PRS-WMH~deep temporal~ age_scan + Sex	0.075	**0.016**	0.014	0.135	0.060
PRS-WMH~juxtacortical frontal~ age_scan + Sex	0.065	**0.032**	0.006	0.125	0.069
PRS-WMH~juxtacortical parietal~ age_scan + Sex	0.059	0.054	−0.001	0.119	0.090
PRS-WMH~juxtacortical occipital~ age_scan + Sex	0.054	0.083	−0.007	0.114	0.113
PRS-WMH~juxtacortical temporal~ age_scan + Sex	0.003	0.922	−0.057	0.063	0.922
Model 1					
PRS-WMH~periventricular basal.ganglia~ age_scan + Sex + Hypertension	0.023	0.455	−0.037	0.082	0.525
PRS-WMH~deep basal.ganglia~ age_scan + Sex + Hypertension	0.028	0.366	−0.032	0.087	0.458
PRS-WMH~juxtacortical basal.ganglia~ age_scan + Sex + Hypertension	0.020	0.504	−0.039	0.079	0.540
PRS-WMH~periventricular frontal~ age_scan + Sex + Hypertension	0.079	**0.010**	0.019	0.139	0.075
PRS-WMH~periventricular parietal~ age_scan + Sex + Hypertension	0.049	0.117	−0.012	0.109	0.164
PRS-WMH~periventricular occipital~ age_scan + Sex + Hypertension	0.088	**0.004**	0.028	0.147	0.060
PRS-WMH~periventricular temporal~ age_scan + Sex + Hypertension	0.051	0.101	−0.010	0.111	0.164
PRS-WMH~deep frontal~ age_scan + Sex + Hypertension	0.068	**0.024**	0.009	0.128	0.094
PRS-WMH~deep parietal~ age_scan + Sex + Hypertension	0.063	**0.042**	0.002	0.123	0.105
PRS-WMH~deep occipital~ age_scan + Sex + Hypertension	0.067	**0.032**	0.006	0.127	0.096
PRS-WMH~deep temporal~ age_scan + Sex + Hypertension	0.070	**0.025**	0.009	0.130	0.094
PRS-WMH~juxtacortical frontal~ age_scan + Sex + Hypertension	0.059	0.053	−0.001	0.118	0.114
PRS-WMH~juxtacortical parietal~ age_scan + Sex + Hypertension	0.048	0.120	−0.013	0.108	0.164
PRS-WMH~juxtacortical occipital~ age_scan + Sex + Hypertension	0.056	0.069	−0.004	0.116	0.129
PRS-WMH~juxtacortical temporal~ age_scan + Sex + Hypertension	0.000	0.999	−0.060	0.060	0.999
Model 2					
PRS-WMH~periventricular basal.ganglia~ age_scan + Sex + CAIDE-I	0.032	0.295	−0.028	0.091	0.340
PRS-WMH~deep basal.ganglia~ age_scan + Sex + CAIDE-I	0.034	0.268	−0.026	0.093	0.335
PRS-WMH~juxtacortical basal.ganglia~ age_scan + Sex + CAIDE-I	0.023	0.447	−0.036	0.081	0.479
PRS-WMH~periventricular frontal~ age_scan + Sex + CAIDE-I	0.090	**0.003**	0.030	0.149	0.023
PRS-WMH~periventricular parietal~ age_scan + Sex + CAIDE-I	0.056	0.070	−0.005	0.116	0.102
PRS-WMH~periventricular occipital~ age_scan + Sex + CAIDE-I	0.089	**0.003**	0.030	0.148	**0.023**
PRS-WMH~periventricular temporal~ age_scan + Sex + CAIDE-I	0.062	**0.045**	0.001	0.122	0.084
PRS-WMH~deep frontal~ age_scan + Sex + CAIDE-I	0.078	**0.010**	0.019	0.137	0.050
PRS-WMH~deep parietal~ age_scan + Sex + CAIDE-I	0.068	**0.027**	0.008	0.128	0.068
PRS-WMH~deep occipital~ age_scan + Sex + CAIDE-I	0.070	**0.024**	0.009	0.130	0.068
PRS-WMH~deep temporal~ age_scan + Sex + CAIDE-I	0.075	**0.016**	0.014	0.135	0.060
PRS-WMH~juxtacortical frontal~ age_scan + Sex + CAIDE-I	0.065	**0.032**	0.005	0.124	0.069
PRS-WMH~juxtacortical parietal~ age_scan + Sex + CAIDE-I	0.058	0.058	−0.002	0.118	0.097
PRS-WMH~juxtacortical occipital~ age_scan + Sex + CAIDE-I	0.055	0.075	−0.006	0.115	0.102
PRS-WMH~juxtacortical temporal~ age_scan + Sex + CAIDE-I	0.004	0.899	−0.056	0.064	0.899
Model 3					
PRS-WMH_noAPOE_~periventricular basal.ganglia~ age_scan + Sex + APOE_e4_carriership	0.034	0.259	−0.025	0.094	0.363
PRS-WMH_noAPOE_~deep basal.ganglia~ age_scan + Sex + APOE_e4_carriership	0.052	0.091	−0.008	0.111	0.228
PRS-WMH_noAPOE_~juxtacortical basal.ganglia~ age_scan + Sex + APOE_e4_carriership	0.013	0.671	−0.046	0.071	0.671
PRS-WMH_noAPOE_~periventricular frontal~ age_scan + Sex + APOE_e4_carriership	0.062	**0.041**	0.002	0.122	0.123
PRS-WMH_noAPOE_~periventricular parietal~ age_scan + Sex + APOE_e4_carriership	0.034	0.266	−0.026	0.094	0.363
PRS-WMH_noAPOE_~periventricular occipital~ age_scan + Sex + APOE_e4_carriership	0.064	**0.035**	0.005	0.124	0.123
PRS-WMH_noAPOE_~periventricular temporal~ age_scan + Sex + APOE_e4_carriership	0.040	0.191	−0.020	0.099	0.358
PRS-WMH_noAPOE_~deep frontal~ age_scan + Sex + APOE_e4_carriership	0.067	**0.027**	0.008	0.127	0.123
PRS-WMH_noAPOE_~deep parietal~ age_scan + Sex + APOE_e4_carriership	0.071	**0.021**	0.010	0.131	0.123
PRS-WMH_noAPOE_~deep occipital~ age_scan + Sex + APOE_e4_carriership	0.022	0.478	−0.039	0.082	0.598
PRS-WMH_noAPOE_~deep temporal~ age_scan + Sex + APOE_e4_carriership	0.075	**0.017**	0.014	0.136	0.123
PRS-WMH_noAPOE_~juxtacortical frontal~ age_scan + Sex + APOE_e4_carriership	0.047	0.128	−0.013	0.106	0.274
PRS-WMH_noAPOE_~juxtacortical parietal~ age_scan + Sex + APOE_e4_carriership	0.038	0.217	−0.022	0.098	0.362
PRS-WMH_noAPOE_~juxtacortical occipital~ age_scan + Sex + APOE_e4_carriership	0.016	0.599	−0.044	0.077	0.671
PRS-WMH_noAPOE_~juxtacortical temporal~ age_scan + Sex + APOE_e4_carriership	0.013	0.664	−0.047	0.073	0.671

CI, confidence interval; OR, Odds Ratio; *ρ*, Covariate-adjusted Spearman’s correlation coefficient; SE, standard error. Age and PRS-WMH were standardized when included in the logistic regression model. The non-pathological group served as the reference category for WMH pathological levels in the logistic regression model. *p*-values below the established significance level (.05) are highlighted in bold.

### 3.3 The polygenic risk score of WMH reflects higher WMH volume in cardiometabolic risk groups, despite low cardiovascular risk for late-life dementia

**Figure 2:**
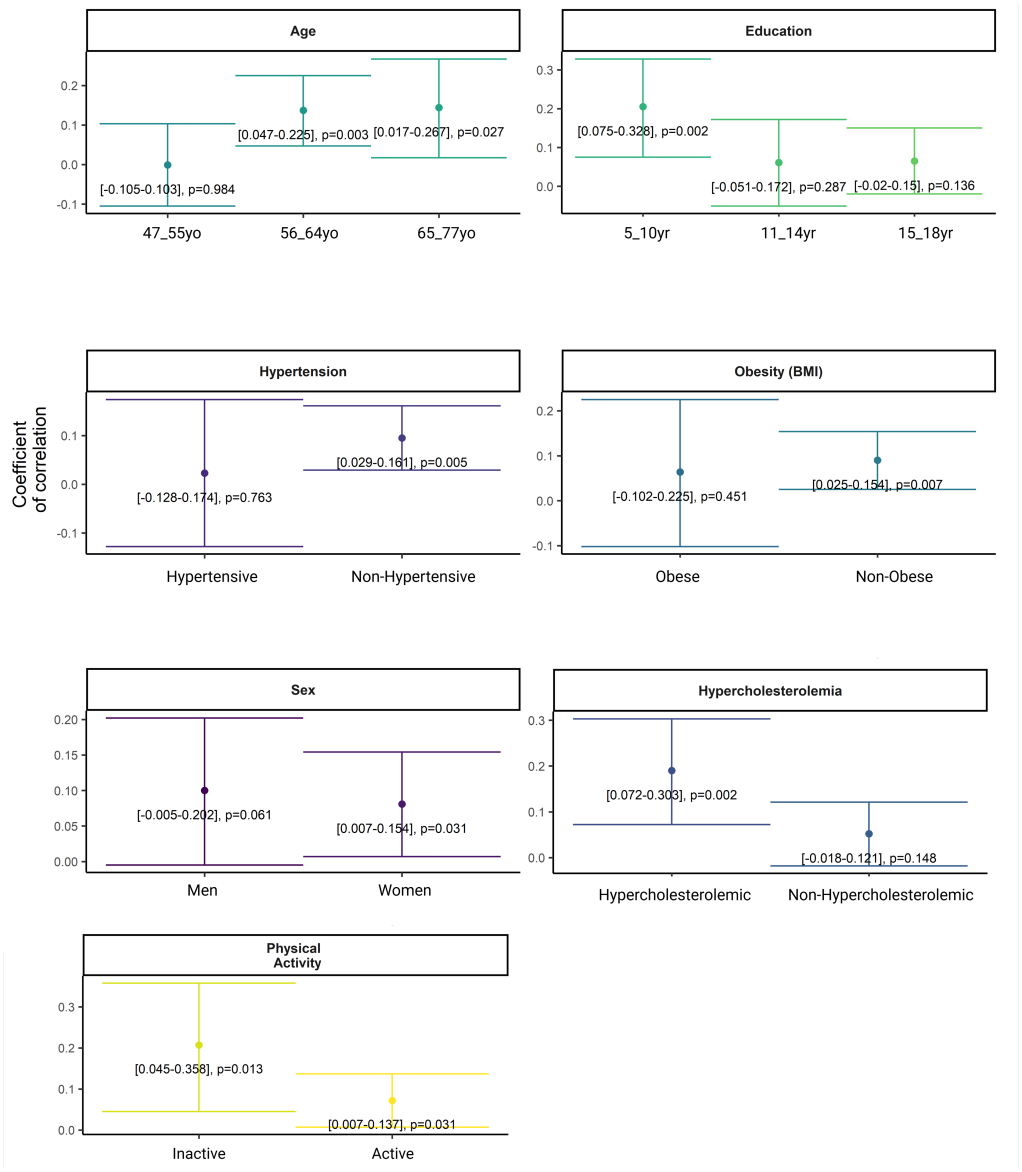
Stratified covariate-adjusted Spearman’s rank correlation test assessing the association between WMHV and PRS-WMH in individuals at low cardiovascular risk for late-life dementia. Models were stratified by CAIDE-I components. Age and sex were included as covariates when required. Confidence intervals and *p*-values were reported.

Genetic predisposition to WMH was positively associated with larger WMHV in non-hypertensives (*ρ* = 0.095 [0.029, 0.161], *p =* 0.005) and non-obese (*ρ* = 0.09 [0.025, 0.154], *p =* 0.007) individuals at low cardiovascular risk for late-life dementia (Table 3; Figure 2). Non-hypertensive individuals were mainly younger (*p =* 1.26×10^−10^), with a higher proportion of non-obese (*p =* 8.60×10^−07^) and non-hypercholesterolemic (*p =* 1.69×10^−04^) participants and higher percentage of women (*p =* 5.14×10^−03^) when compared to their counterparts (Supplementary Figure 6). Non-obese individuals were physically more active (*p =* 4.66×10^−04^) than their counterparts, characterized by a higher percentage of low educated (*p =* 0.02) and non-hypertensive (*p =* 8.60×10^−07^) individuals, when compared to obese group (Supplementary Figure 7). Moreover, genetic predisposition to WMH was positively associated with larger WMHV in women (*ρ* = 0.081 [0.007, 0.154], *p =* 0.031), hypercholesterolemic individuals (*ρ* = 0.19 [0.072, 0.303], *p =* 0.002) and participants with less than 11 years of education (*ρ* = 0.205 [0.075, 0.328], *p =* 0.002; Table 3; Figure 2). Women were characterized by a higher percentage of low educated (*p =* 3.74×10^−04^) and non-hypertensive (*p =* 5.14×10^−03^) individuals than men (Supplementary Figure 8). In the hypercholesterolemic group we observed a higher proportion of old (*p =* 3.42×10^−10^) and hypertensive participants (*p =* 1.69×10^−04^) when compared to their counterparts (Supplementary Figure 9). In the group with lower educational attainment (5–10 years) we found a higher proportion of non-obese individuals (*p =* 0.02), and women (*p =* 3.74×10^−04^; Supplementary Figure 10). Similarly, in participants older than 55 years, WMHV were positively correlated with a higher genetic predisposition to WMH (56–64 years-old group: *ρ* = 0.137 [0.047, 0.225], *p =* 0.003; 65–77 years-old group: *ρ* = 0.144 [0.017, 0.267], *p =* 0.027; Table 3; Figure 2). In the group of individuals older than 55, we found a higher proportion of physically active participants than in the youngest group (*p =* 3.81×10^−03^). Moreover, older participants were characterized by a higher percentage of hypertensive (*p =* 3.42×10^−10^) and hypercholesterolemic (*p =* 1.26×10^−10^) individuals when compared to their counterparts (Supplementary Figure 11). Regarding physical activity, genetic predisposition to WMH was positively associated with larger WMHV in both groups, although the correlation was stronger in the inactive group (*ρ* = 0.207 [0.045, 0.368], *p =* 0.013) than in the active one (*ρ* = 0.072 [0.007, 0.137], *p =* 0.031; Table 3; Figure 2). The inactive group was mainly characterized by a higher proportion of young (*p =* 3.81×10^−03^), obese (*p =* 4.66×10^−04^) and low-educated (*p =* 0.05) individuals when compared to the active group (Supplementary Figure 12).

**Table 3:** Results for the covariate-adjusted Spearman’s rank correlation test assessing the association between WMHV and PRS-WMH stratifying by the CAIDE-components.

Groups	Model	*ρ*	*p*-value	Low CI	High CI
Women	PRS-WMH|WMH volume ~ age_scan	0.081	**0.031**	0.007	0.154
Men	PRS-WMH|WMH volume ~ age_scan	0.1	0.061	−0.005	0.202
Hypertensive	PRS-WMH|WMH volume ~ age_scan + Sex	0.023	0.763	−0.128	0.174
Non-Hypertensive	PRS-WMH|WMH volume ~ age_scan + Sex	0.095	**0.005**	0.029	0.161
Hypercholesterolemic	PRS-WMH|WMH volume ~ age_scan + Sex	0.19	**0.002**	0.072	0.303
Non-Hypercholesterolemic	PRS-WMH|WMH volume ~ age_scan + Sex	0.052	0.148	−0.018	0.121
Obese	PRS-WMH|WMH volume ~ age_scan + Sex	0.064	0.451	−0.102	0.225
Non-Obese	PRS-WMH|WMH volume ~ age_scan + Sex	0.09	**0.007**	0.025	0.154
Age 47–55 yo	PRS-WMH|WMH volume ~ Sex	−0.001	0.984	−0.105	0.103
Age 56–64 yo	PRS-WMH|WMH volume ~ Sex	0.137	**0.003**	0.047	0.225
Age 65–77 yo	PRS-WMH|WMH volume ~ Sex	0.144	**0.027**	0.017	0.267
Education 5–10 yr	PRS-WMH|WMH volume ~ age_scan + Sex	0.205	**0.002**	0.075	0.328
Education 11–14 yr	PRS-WMH|WMH volume ~ age_scan + Sex	0.061	0.287	−0.051	0.172
Education 15–18 yr	PRS-WMH|WMH volume ~ age_scan + Sex	0.065	0.136	−0.02	0.15
Active	PRS-WMH|WMH volume ~ age_scan + Sex	0.072	**0.031**	0.007	0.137
Inactive	PRS-WMH|WMH volume ~ age_scan + Sex	0.207	**0.013**	0.045	0.358

CI, confidence interval; *ρ*, Covariate-adjusted Spearman’s correlation coefficient. *p*-values below the established significance level (.05) are highlighted in bold.

### 3.4 Genetic factors conferring higher risk to WMH are mainly involved in vascular, cellular and neural function highly interconnected through lipid-regulatory mechanisms

**Figure 3:**
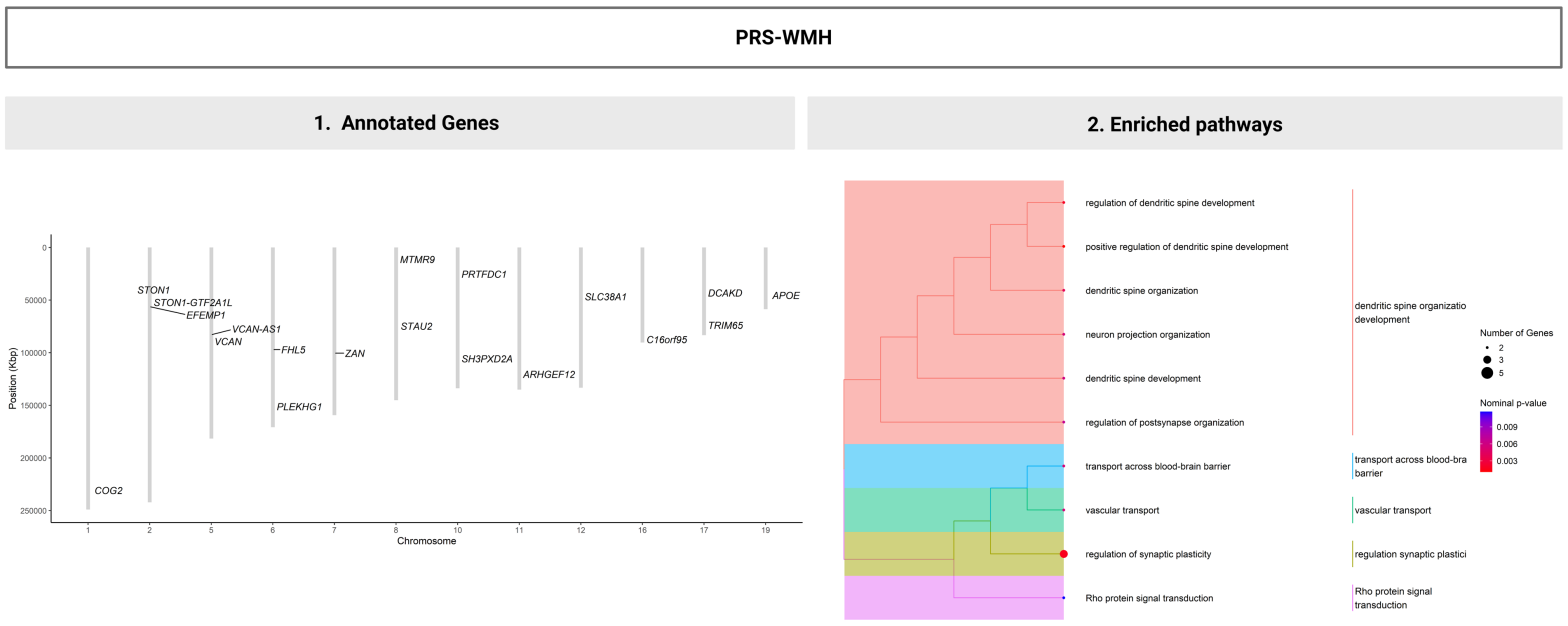
(1) Gene annotation of the variants included in the polygenic risk score of WMH and (2) results of the enrichment analysis showing the main biological processes in which post-clumped SNP-annotated genes are involved. One-sided version of Fisher’s exact test was performed to determine whether known biological functions were overrepresented or enriched in the gene list and calculate the probability of observing a set of genes in a particular biological pathway by chance. Significant results were reported at nominal *p* < 0.05 and biological mechanisms were grouped into main functions based on their similarity.

Most of the SNPs included in the PRS_WMH_ after clumping (*n =* 25) were located in chromosomes 2, 6, 10, 16, and 17. Among them, around 25% (*n =* 6) had a regulatory role and were found to be associated with gene expression levels (i.e., eQTLS; Supplementary Table 5). These SNPs have been related to several GWAS-catalog specific traits, such as AD, C-reactive protein, HDL, parental longevity, serum alanine aminotransferase, WM microstructure, cerebral amyloid deposition, hypertension and amyloid-*ꞵ* 42 levels (Supplementary Results). The majority of genes mapped to these SNPs relate to additional traits such as hemoglobin measurement, mean corpuscular hemoglobin concentration, coronary artery disease, diverticular disease and hematocrit, among others. Enrichment analysis of the genes identified significantly enriched biological pathways (*p* < 0.05) mainly involved in neuronal structure and synaptic organization and vascular-related processes, mainly related to brain–blood barrier and transport (Figure 3; Supplementary Table 5). From a general overview, the enrichment analysis results of the non-cohort specific SNPs related to WMHV, suggest an overall contribution of these genes into lipoprotein metabolic processes (Supplementary Table 6).

### 3.5 Blood pressure measurements are associated with larger WMHV and higher polygenic risk of WMH

**Figure 4:**
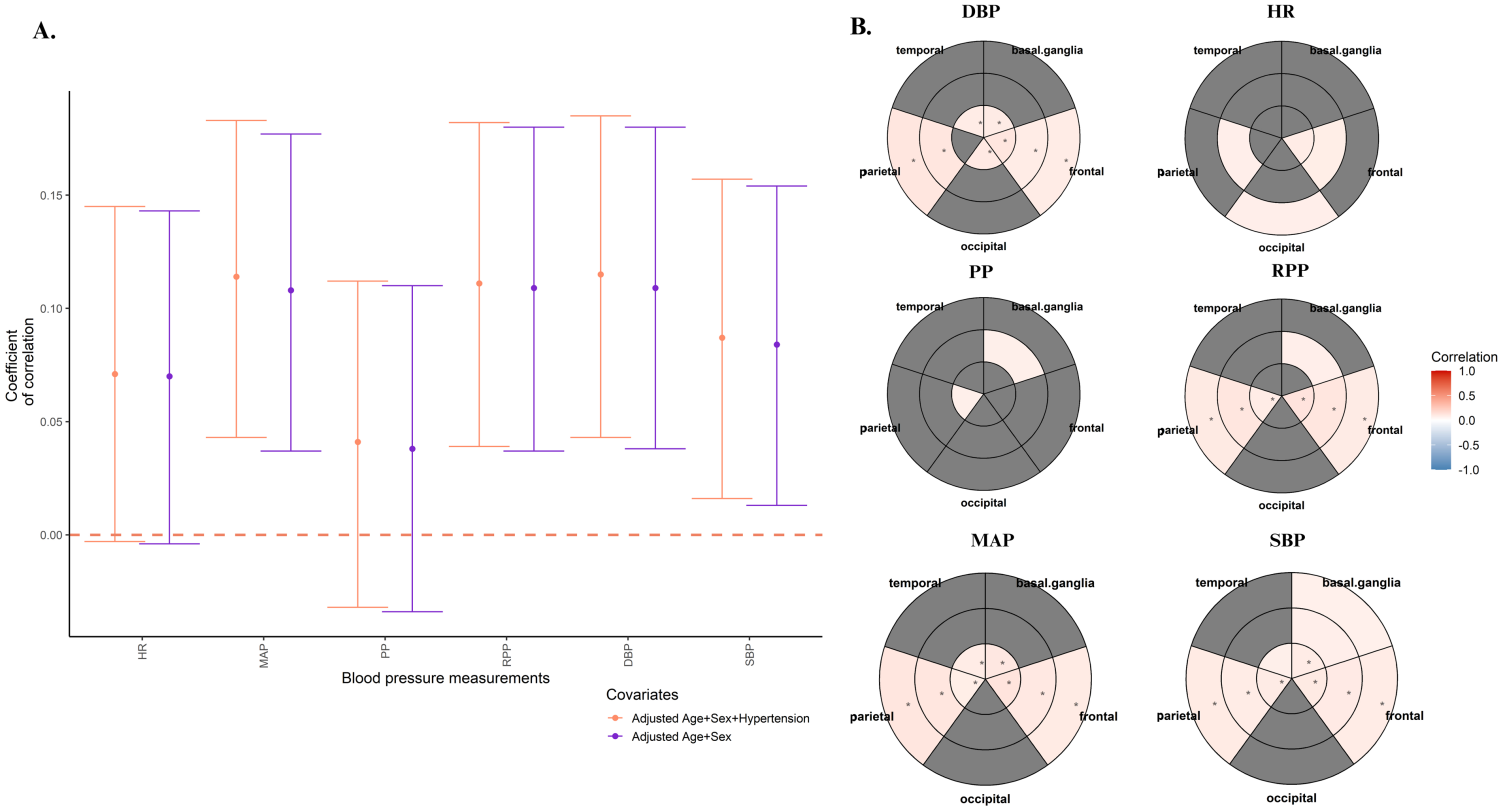
Covariate adjusted Spearman’s rank correlation test assessing the association between blood pressure measurements and both **(A)** global and **(B)** regional WMHV. The main model included age and sex as covariates. Sensitivity analyses additionally included hypertension status, which was defined based on both the use of antihypertensive and SBP levels. **(A)** A forest plot was used to display the results of the partial correlation between the blood pressure measurements and global WMHV. Confidence intervals were reported (α = 0.05, 95% CI). Dashed line indicates no correlation (*p* = 0).**(B)** Bullseye plots were used to indicate the correlation between the blood pressure measurement and regional WMHV. Concentric circles indicate the distance of the lesion, from internal layers referring to periventricular WMHV to more external layers referring to juxtacortical WMHV. Significant correlations (nominal *p* < 0.05) were indicated by colouring the cells in the bullseye plot. Gray areas indicate non-significant correlations. The asterisk indicates FDR <. 05. HR, Heart Rate; MAP, Mean Arterial Pulse; PP, Pulse Pressure; RPP, Rate-pressure Product; DBP, Diastolic blood pressure; SBP, Systolic blood pressure.

The association between cardiometabolic traits and WMHV was explored. Partial Spearman’s rank correlation test showed a significant correlation between both SBP (*ρ* = 0.087 [0.016, 0.157], *p =* 0.016) and DBP (*ρ* = 0.115 [0.043, 0.185], *p =* 0.002) with higher WMHV (Table 4), even when adjusting for hypertension status (Figure 4A). Similarly, MAP (*ρ* = 0.114 [0.043, 0.183], *p =* 0.002) and RPP (*ρρρ* = 0.111 [0.039, 0.182], *p =* 0.003) also displayed significant correlations with larger WMHV (Table 4), even after controlling for hypertension status (Figure 4A). HR (*ρ* = 0.071 [−0.003, 0.145], *p =* 0.06) and PP (*ρ* = 0.041 [−0.032, 0.112], *p =* 0.272) showed the same positive trend, but results did not reach statistical significance (Table 4; Figure 4A). At a regional level, SBP was significantly associated with WMHV in frontal and parietal areas (FDR < 0.05), as well as with periventricular WMH in basal ganglia (*ρ* = 0.092 [0.019, 0.164], FDR = 0.03; Figure 4B; Supplementary Table 7). DBP positively correlated with juxtacortical and deep WMHV in frontal and parietal regions, as well as with periventricular WMH in the basal ganglia and frontal, occipital and temporal regions (FDR < 0.05; Figure 4B; Supplementary Table 7). Similarly, RPP and MAP were positively correlated with frontal and parietal WMHV. Additionally, MAP was also positively correlated with periventricular WMHV in the basal ganglia and the temporal lobe. Non-significant correlations were found between regional WMHV and neither HR nor PP (Figure 4B; Supplementary Table 7). The PRS of WMH was uniquely significantly associated with higher RPP (*ꞵ* = 0.074 [0.004, 0.145], *p =* 0.038; Supplementary Table 8). Nonetheless, a positive trend was also observed for the association between the PRS_WMH_ and DBP (*ꞵ* = 0.070 [−0.001, 0.140], *p =* 0.051) and MAP (*ꞵ* = 0.067 [−0.002, 0.136], *p =* 0.058), although results did not reach statistical significance (Supplementary Table 8). Finally, in a subset of participants with available lipids measurements (*N =* 237), displaying dyslipidemia based on either self-reported hypercholesterolemia, use of medication or Tchol ≥ 240 mg/dL, was inversely associated with WMHV. Further description of the results and the sample characteristics can be found in Supplementary Results.

**Table 4:** Results for the covariate-adjusted Spearman’s rank correlation test assessing the association between blood pressure measurements and global WMHV.

Model	*ρ*	*p*-value	Low CI	High CI
DBP| WMHV ~ age_scan + Sex	0.115	**0.002**	0.043	0.185
SBP| WMHV ~ age_scan + Sex	0.087	**0.016**	0.016	0.157
HR| WMHV ~ age_scan + Sex	0.071	**0.06**	−0.003	0.145
PP| WMHV ~ age_scan + Sex	0.041	0.272	−0.032	0.112
RPP| WMHV ~ age_scan + Sex	0.111	**0.003**	0.039	0.182
MAP| WMHV ~ age_scan + Sex	0.114	**0.002**	0.043	0.183

CI, confidence interval; *ρ*, Covariate-adjusted Spearman’s correlation coefficient. *p*-values below the established significance level (.05) are highlighted in bold.

### 3.6 Diastolic blood pressure and mean arterial pressure partially mediate the association between the PRS of WMH and WMHV in low cardiovascular risk individuals

**Figure 5:**
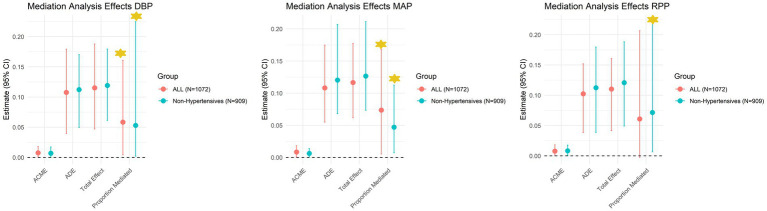
Mediation analysis results exploring the mediator role of blood pressure measurements in the association between the PRS of WMH and WMH in the whole sample of study as well as in non-hypertensive individuals Confidence intervals were reported (α = 0.05, 95%CI). Significant mediated pathways were highlighted with a yellow star symbol. ACME, Average Causal Mediation Effect; ADE, Average Direct Effect.

**Figure 6:**
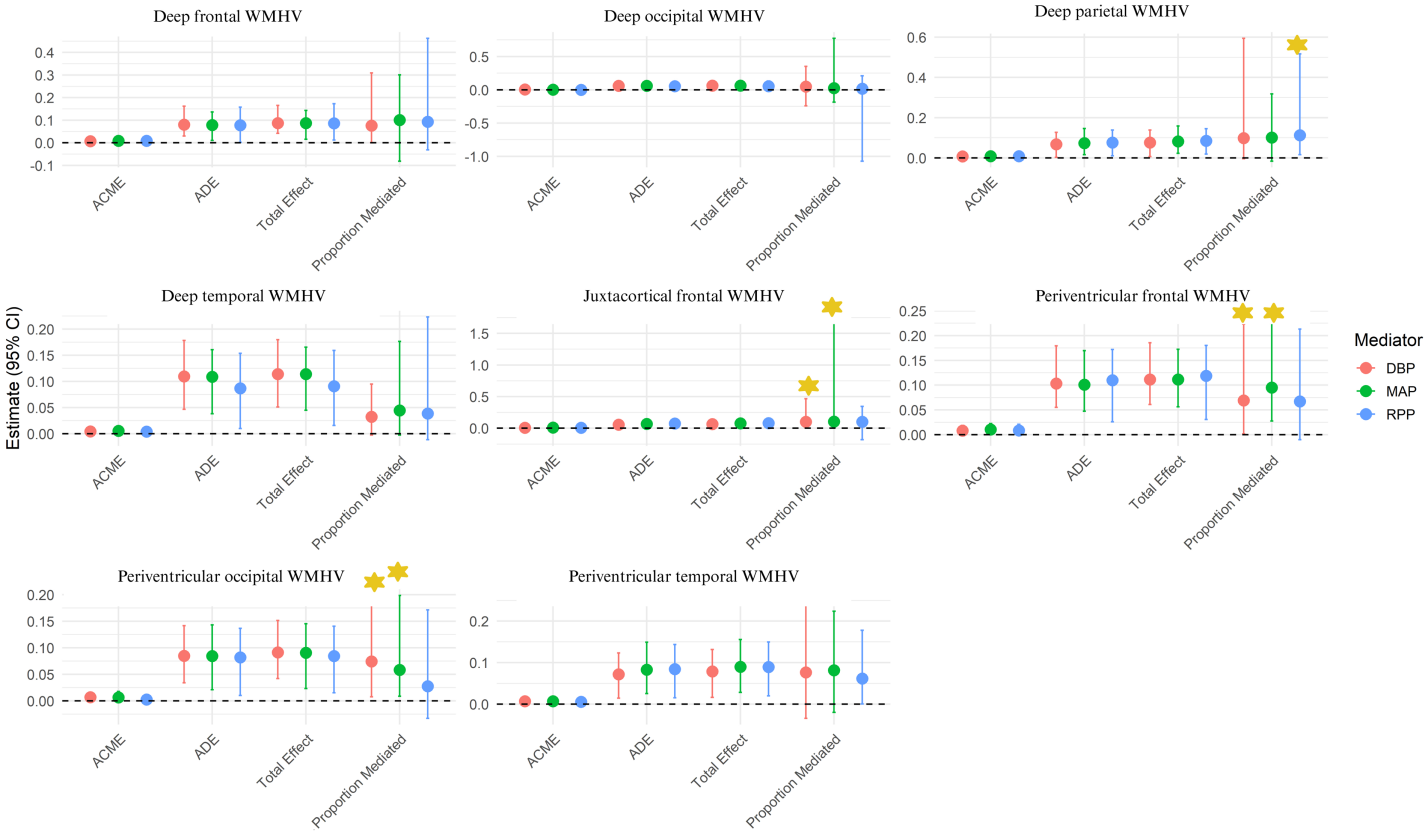
Mediation analysis results exploring the mediator role of DBP, MAP and PP in the association between the PRS of WMH and WMH in the whole sample of study. Confidence intervals were reported (α = 0.05, 95%CI). Significant mediated pathways were highlighted with a yellow star symbol. ACME, Average Causal Mediation Effect; ADE, Average Direct Effect; DBP, Diastolic blood pressure; MAP, Mean arterial pressure; PP, Pulse pressure.

Based on the aforementioned significant associations between the PRS of WMH and BP measurements, as well as on the statistically significant correlations between WMHV and the PRS_WMH_ (Table 2A), mediation analyses were explored. Results showed that DBP (Prop.mediated = 0.06, *p =* 0.04) and MAP (Prop.mediated = 0.08, *p* < 0.001) partially mediated the association between the genetic predisposition to WMH and the observed WMHV both in the whole sample (Table 5; Figure 5). Same partially mediated paths were observed for the subset of non-hypertensive individuals (Figure 5), regardless of the fact that they displayed lower median values for DBP and MAP when compared to hypertensive individuals (Supplementary Results). At a regional level, both DBP and MAP partially mediated the association between the PRS_WMH_ and periventricular frontal (DBP: Prop.mediated = 0.07, *p =* 0.04; MAP: Prop.mediated = 0.095, *p <* 0.001), periventricular occipital (DBP: Prop.mediated = 0.074, *p =* 0.04; MAP: Prop.mediated = 0.058, *p <* 0.001) and juxtacortical frontal (DBP: Prop.mediated = 0.010, *p =* 0.04; MAP: Prop.mediated = 0.105, *p =* 0.04) WMHV (Figure 6; Supplementary Table 9). Additionally, RPP partially mediated the association between the PRS of WMH and deep parietal WMHV (Prop.mediated = 0.113, *p <* 0.001; Figure 6; Supplementary Table 9). In non-hypertensive individuals, the three metrics mediated the association between the PRS of WMH and deep parietal WMHV (DBP: Prop.mediated = 0.087, *p =* 0.04; MAP: Prop.mediated = 0.092, *p <* 0.001; RPP: Prop.mediated = 0.076, *p <* 0.001; Supplementary Figure 13; Supplementary Table 10). Additionally, MAP (Prop.mediated = 0.071, *p <* 0.001) and RPP (Prop.mediated = 0.096 *p <* 0.001) partially mediated the association between the PRS_WMH_ and deep frontal WMHV. RPP partially mediated the association between the PRS of WMH and periventricular frontal WMHV (Prop.mediated = 0.082, *p <* 0.001; Supplementary Figure 13; Supplementary Table 10). Finally, DBP was additionally partially mediating the association between genetic risk of WMH and periventricular temporal WMHV (Prop.mediated = 0.061, *p =* 0.04) in non-hypertensive individuals (Supplementary Figure 13; Supplementary Table 10).

**Table 5:** Results for the mediation analysis exploring the mediator role of DBP, RPP and MAP in the association between the PRS of WMH and WMHV.

Effect	Estimate	*p*-value	Low CI	High CI
DBP				
ACME	0.008	**0.040**	0.001	0.020
ADE	0.109	**<0.001**	0.051	0.164
Total Effect	0.117	**<0.001**	0.063	0.174
Proportion Mediated	0.062	**0.040**	0.008	0.241
RPP				
ACME	0.008	0.080	0.000	0.018
ADE	0.111	**<0.001**	0.056	0.164
Total Effect	0.119	**<0.001**	0.061	0.171
Proportion Mediated	0.070	0.080	0.001	0.171
MAP				
ACME	0.010	**<0.001**	0.001	0.022
ADE	0.106	**<0.001**	0.055	0.155
Total Effect	0.116	**<0.001**	0.064	0.172
Proportion Mediated	0.082	**<0.001**	0.013	0.252

CI, confidence interval; ACME, Average Causal Mediation Effect; ADE, Average Direct Effect. *p*-values below the established significance level (.05) are highlighted in bold.

## 4 Discussion

In this study, we explored potential biomarkers of WMH in a sample of CU middle-aged and older individuals at low cardiovascular risk for dementia, for whom larger WMHV were associated with lower executive function. This setting enabled the investigation of the biological processes associated with developing WMH in CU participants in the context of AD prevention, without major confounding effects of cardiovascular risk factors. Our findings show that vascular mechanisms lead to WMH and demonstrate that blood pressure measures in midlife, independently of the clinical definition of hypertension, were associated with larger WMHV. These results underscore the potential of genetics for identifying middle-aged and older at-risk asymptomatic populations susceptible to benefiting from stricter hypertension control to further prevent cognitive decline.

In the sample of the study, which was characterized by displaying low cerebrovascular burden but still contributing to poorer EF, the polygenic risk of WMH was significantly associated with actual global and regional WMHV, specifically in the frontal lobe. WMH risk was mainly driven by vascular, neuronal and cellular processes. The construction of the PRS incorporated suggestive genome-wide significant hits (i.e., *p =* 5·10^−6^) previously reported to be associated with WMHV (29). Most of the genome-wide significant SNPs were located in chromosomes 2 (e.g., STON1, COG2), chr6 (e.g., PLEKHG1, FHL5), chr10 (e.g., PRTFDC1, SH3PXD2A), chr16 (e.g., C16orf95, SLC38A1) and chr17 (e.g., DCAKD, TRIM65). These loci have been associated with other traits beyond WMH, such as AD, HDL, cerebral amyloid deposition, hypertension and coronary artery disease, among others (29). In the WMH-GWAS of reference (29), post-GWAS analyses showed that genome-wide significant hits mapped to genes predominantly involved in the regulation of atrial cardiac muscle cell action potential and post-embryonic eye morphogenesis. Non-cohort specific gene-set enrichment analysis (i.e., inclusion of pre-clumping SNP-annotated genes) revealed lipid-regulatory mechanisms as main biological pathways associated with risk of WMH.

Based on the enriched biological pathways, we explored two main mechanisms as mediating pathways in the association between the genetic vulnerability to WMH and the actual WMHV. First, we explored the potential mediating role of dyslipidemia status. Nonetheless, less than 25% of the sample had available lipid measurements, which made the mediation analysis not possible. However, when we performed the association models between dyslipidemia status and WMHV, we observed that dyslipidemia was negatively associated with WMHV, contrary to what we hypothesized. The definition of dyslipidemia was based on either self-reported hypercholesterolemia status, use of medication or lipid levels in serum. Therefore we should cautiously interpret these findings in our sample, where around 30% of the participants were using lipid-modifying medication. Results in this subset of the study sample lead us to hypothesize that either (a) we did not have robust cross-sectional lipid measurements to define dyslipidemia status, due to the influential effect of sustained use of medication over time; or that (b) dyslipidemia status was not a good proxy of the lipidic-related mechanisms linking genetic vulnerability to WMHV (e.g., cholesterol efflux (58) or other different from lipid levels); or finally that (c) based on the sample characteristics, other measurements could be driving this genetic vulnerability in the sample. Although, there is evidence in the literature on the association between lipid levels and WMHV (5, 59, 60) as well as on the use of statins and its contribution on reducing cerebrovascular burden (61) and the incidence of cardiovascular events (62), we could not prove any of these findings with the available data.

Second, we explored the potential mediating role of BP measurements, as vascular transport and transport across the blood–brain barrier (BBB) emerged as two important pathways in the cohort-based enrichment analysis. Results showed that in the study sample, as well as in the subset of non-hypertensive individuals, hemodynamic measures of blood flow were partially mediating the association between genetic vulnerability to WMH and WMHV. Specifically, DBP and MAP were partially mediating the association between higher genetic risk of WMH and larger global WMH burden. DBP, MAP and RPP also acted as partial mediators in the relationship between the genetic risk of WMH and regional WMHV, especially in periventricular frontal and parietal areas. Recent population-based studies including European adults, have shown that elevated MAP and PP, are associated with larger WMHV and white matter injury (63). A potential mechanistic explanation, proposed by Kaul and Rubinstein (64), suggests that elevated PP and MAP contribute to WMH by inducing BBB dysfunction through microvascular permeability, as well as activation of proinflammatory genes, that promote an ischemic environment in the brain, facilitating amyloid-*β* deposition and white matter injury. Regarding the role of DBP, in a recent study (65) authors found that before the age of 50, DBP is strongly associated with WMH, and later on, both concurrent and past elevated SBP and DBP measurements impact WMHV, with SBP showing a greatest contribution to WMH severity. In our sample, although SBP was associated with both regional and global WMH burden, DBP appeared to play a central mediating role between participants’ genetic vulnerability and their current cerebrovascular burden. While SBP may likely reflect cumulative damage, influenced by arterial stiffening and age, DBP may be a more sensitive intermediary linking inherited vascular vulnerability to ongoing cerebrovascular changes, as supported by combined transcriptome-wide association studies with colocalization analyses that linked WMH risk to artery-specific and extracellular-matrix genes (29). These observations reinforce the link between expression levels of specific genes and WMHV, strengthening the mechanistic chain from genetic vulnerability of WMH to WMHV through arterial-related events.

To further examine the stability of the aforementioned association between the genetic risk of WMH and global WMHV, we aimed to identify genetically vulnerable groups for whom the association between the PRS_WMH_ and WMHV remained consistent, regardless of the vascular risk profile. Results showed that higher genetic risk of WMH was associated with larger WMHV in risk groups defined by older age, low educational attainment and hypercholesterolemia. A recent study identified hyperlipidemia as one of the greatest modifiable risk factors for WMH (66). These findings underscore the potential role of lipid metabolism dysfunction in the development of WMH (67). In the study sample, hypercholesterolemic individuals were older and had a higher prevalence of hypertensive individuals compared to the non-hypercholesterolemic group. Nonetheless, age and hypertension were not driving the association between WMHV and the PRS_WMH_ in hypercholesterolemic, as the correlation remained significant after adjusting for both risk factors. This observation proposes hypercholesterolemic individuals as a vulnerable population for higher cerebrovascular burden. According to the observed association in individuals with low educational attainment, a study showed a direct negative association between education and WMHV in the general population (2). This negative association could be explained by differences in socioeconomic (68) and lifestyle factors (69) between low- and high-educated individuals that contribute to the presence of WMH. Finally, the PRS_WMH_ was also associated with larger WMHV in individuals older than 55 years. Age is the main non-modifiable risk factor for WMH. These radiological features are common in healthy middle-aged individuals, with a prevalence ranging from 40 to 70% in the fifth decade of age (70). On the contrary, the PRS_WMH_ was related to larger WMHV in the non-risk group for hypertension, the main modifiable risk factor of WMH. A recent multi ancestry meta-analysis of WMH-GWAS (71) showed new *loci* significantly associated with WMHV independently of hypertension status, suggesting the relevant role of genetics explaining the presence of WMHV even in groups clinically defined as non-risk groups (i.e., normotensive individuals). Moreover, significant associations between the PRS_WMH_ and larger WMHV were also found in the non-risk group for obesity, in women, and in both groups for physical activity. It is possible that, in that case of non-obese individuals, genetic susceptibility to WMH could manifest in “relatively favorable” environments, whereas in the high-risk group other modifiable risk factors had a major impact. According to the observed association between higher genetic risk of WMH and larger WMHV in women, sex-specific vascular biology and lipid signaling could differentially impact cerebrovascular burden in women, increasing its risk for larger WMH via vascular processes (72).

Finally, our analysis evaluating the association between the PRS_WMH_ and pathological WMH levels, suggested that genetic risk of WMH correlated with the continuous WMHV but may not be a good tool to predict WMH severe cases, at least in a sample with low cerebrovascular burden and at low cardiovascular risk. The lack of association between the PRS of WMH and pathological WMH levels could be attributed to the sample size or to the dichotomisation of the variable. The study sample included a small proportion of participants having Fazekas scores greater than one (~8%). This limited number of pathological cases affected the sample size when categorizing individuals into severity groups, thereby reducing the statistical power of the analysis. Finally, SNPs included in the computation of the PRS_WMH_ were obtained from a community-based GWAS that worked with WMH as a continuous outcome (29). Therefore, the effect of these SNPs does more likely sum up to an additive genetic risk score related to continuous WMHV, rather than WMH pathological levels. Altogether these factors may collectively contribute to the lack of association with WMH severity.

Overall, this study identified mechanistic pathways leading to larger WMHV and genetically vulnerable populations that, regardless of their vascular risk profile, displayed higher genetic vulnerability for cerebrovascular burden. Our study emphasizes the suitability of the ALFA study, composed of CU individuals enriched with genetic factors for dementia, to identify mechanisms related to cerebrovascular burden in the context of AD prevention, as remarkably, WMH already relate to poorer EF in this healthy sample. Although we did not follow an hypothesis-driven approach, our data-driven multi-stage exploratory framework succeeded in identifying the biological pathways leading to higher WMHV in a cohort at higher risk of AD. The results of the study highlight the role of BP measures in the presence of WMH among CU asymptomatic individuals that display a healthier cardiovascular profile than expected from an age-matched cohort selected from the general population, as the ALFA study did not include individuals with relevant medical pathology or neurological diseases (34). Nonetheless, we should cautiously interpret these results, as they might not be generalizable to general populations, for which genetic vulnerability to cerebrovascular disease through these mechanisms can be confounded or decreased by co-occurring factors (e.g., obesity, smoking, diabetes).

In summary, our results suggest that in absence of acute WMH burden, cognitive impairment or risk for dementia, genetic information can be used to identify genetically vulnerable populations and potential biomarkers of WMH. Therefore, screening and long-term follow-up of these clinically healthy yet genetically susceptible populations are essential for effective preventive strategies. These findings can serve as the basis of future hypothesis-driven studies, specifically proposing hemodynamic monitoring to reduce cerebrovascular burden and prevent cognitive decline and dementia in genetically vulnerable populations.

Nonetheless, this study also presents some limitations. The ALFA cohort is a cognitively healthy research-volunteer cohort, which biases the selection of participants towards healthier profiles than those identified in general population-based studies. While ALFA offers a suitable cohort to examine underlying mechanisms occurring in CU individuals at preclinical stages of AD, it lacks generalizability to general populations. The replicability of the PRS and generalizability of the observations should be further explored in larger cohorts of cognitively healthy participants with similar cardiovascular profiles. Moreover, the PRS was computed based on the SNPs associated with WMH in European populations. Therefore, generalizability to non-European requires dedicated validation. Additionally, the use of the PRS in the context of this study served for identifying at-risk individuals and revealing vascular mechanisms leading to WMH, but it should not be interpreted as a clinical tool to classify cases of WMH severity. Moreover, beyond the WMH-GWAS-specific variants included in the PR_WMH_, other disease-related genetic factors may be associated with WMHV. Therefore, exploring a larger set of genetic variants or a global PRS for cerebrovascular risk, could reveal alternative pathways leading to larger WMH. Regarding group vulnerabilities, results of the stratified models should be interpreted with caution due to the limited sample size within the risk groups and the absence of a multivariate approach to comprehensively define the cardiovascular risk profile of genetically susceptible individuals to cerebrovascular burden. However, the stratified non-parametric approach allowed us to explore the potential effect modification of risk factors by identifying subgroup-specific patterns in the association between the PRS_WMH_ and WMHV, informing hypotheses about interactions that could be tested in parametric models when model assumptions are fulfilled. Additionally, our results can not inform about the complex interplay between modifiable and non-modifiable risk factors contributing to WMH severity. The present study has a cross-sectional design and does not evaluate the relative contribution of genetics to the rate of progression of WMHV. We are currently working with a smaller study sample, with available WMHV measured at two different time points, to better understand how both genetics and modifiable risk factors contribute to WMH progression and how it impacts cognitive performance over a 3-year period.

In conclusion, our study identified middle-aged and older individuals with no cognitive impairment who are genetically at risk of cerebrovascular disease, primarily through mechanisms involving cardiac function and systemic arterial pressure. These findings highlight a potential therapeutic pathway, via hemodynamic monitoring and the maintenance of stable BP, that may help reduce cerebrovascular burden and support brain health in CU asymptomatic individuals with a low cardiovascular risk profile.

## Data Availability

The data analyzed in this study is subject to the following licenses/restrictions: de-identified data supporting the findings of this study are available upon request from the corresponding author (NV-T). Requests are evaluated by the Scientific Committee at the Barcelonaβeta Brain Research Center and, if granted, data are shared and regulated by a Data Sharing Agreement. Requests to access these datasets should be directed to nvilor@barcelonabeta.org.

## Group member of the ALFA Study

Collaborators of the ALFA Study are: Federica Anastasi, Felipe Hernández-Vilamizar, Armand Gonzalez-Escalante, Annabella Beteta, Raffaele Cacciaglia, Lidia Canals, Alba Cañas, Carme Deulofeu, Maria Emilio, Marta del Campo, Carme Deulofeu, Ruth Dominguez, Maria Emilio, Sherezade Fuentes, Marina García, Gema Huesa, Jordi Huguet, Laura Iglesias, Esther Jiménez, Helena Blasco, Javier Torres, David López-Martos, Paula Marne, Tania Menchón, Paula Ortiz-Romero, Marina de Diego, José Contador-Muñana, Wiesje Pelkmans, Albina Polo, Sandra Pradas, Mahnaz Shekari, Lluís Solsona, Anna Soteras, Núria Tort-Colet and Marc Vilanova.

## Glossary

AD: Alzheimer’s disease
ALFA: ALzheimer’s and FAmilies
APOE: Apolipoprotein E
BMI: Body mass index
CAIDE: Cardiovascular Risk Factors, Aging, and Incidence of Dementia
CU: Cognitively unimpaired
CVD: Cardiovascular Disease
CSVD: Cerebral Small Vessel Disease
CVRF: Cardiovascular Risk Factors
DNA: Deoxyribonucleic acid
DBP: Diastolic blood pressure
GWAS: Genome-wide association studies
HDL: High-density lipoprotein
HR: Heart rate
LDL: Low-density lipoprotein
MAP: Mean arterial pressure
MRI: Magnetic resonance imaging
PP: Pulse pressure
PRS: Polygenic risk score
RPP: Rate-pressure product
SBP: Systolic blood pressure
SNP: Single nucleotide polymorphism
Tchol: Total cholesterol
TG: Triglycerides
TIV: Total intracranial volume
WMH: White matter hyperintensities
WMHV: White matter hyperintensities volumes

## References

[1] Garnier-Crussard A Cotton F Krolak-Salmon P Chételat G. White matter Hyperintensities in Alzheimer’s Disease: beyond vascular contribution. Alzheimers Dement. (2023) 19:3738–48. doi: 10.1002/alz.13057 37027506

[2] Habes M Erus G Toledo JB Zhang T Bryan N Launer LJ . White matter Hyperintensities and imaging patterns of brain ageing in the general population. Brain J Neurol. (2016) 139:008. doi: 10.1093/brain/aww008, 26912649 PMC5006227

[3] Hannawi Y Yanek LR Kral BG Vaidya D Becker LC Becker DM . Hypertension is associated with white matter disruption in apparently healthy middle-aged individuals. AJNR Am J Neuroradiol. (2018) 39:2243. doi: 10.3174/ajnr.A5871 30442693 PMC6368444

[4] Salvadó G Brugulat-Serrat A Sudre CH Grau-Rivera O Suárez-Calvet M Falcon C . Spatial patterns of white matter Hyperintensities associated with Alzheimer’s Disease risk factors in a cognitively healthy middle-aged cohort. Alzheimer's Res Ther. (2019) 11:1–14. 30678723 10.1186/s13195-018-0460-1 PMC6346579

[5] Rao C Zhu L Chuanqin Y Zhang S Zha Z Tong G . Association of Novel Lipid Indices with the white matter Hyperintensities in cerebral small vessel Disease: a cross-sectional study. Lipids Health Dis. (2024) 23:333. doi: 10.1186/s12944-024-02318-3 39402569 PMC11472430

[6] Marini S Merino J Montgomery BE Malik R Sudlow CL Dichgans M . Mendelian randomization study of obesity and cerebrovascular Disease. Ann Neurol. (2020) 87:516–24. doi: 10.1002/ana.25686 31975536 PMC7392199

[7] Debette S Schilling S Duperron M-G Larsson SC Markus HS. Clinical significance of magnetic resonance imaging markers of vascular brain injury: a systematic review and Meta-analysis. JAMA Neurol. (2019) 76:81–94. doi: 10.1001/jamaneurol.2018.3122, 30422209 PMC6439887

[8] Karvelas N Elahi FM. White matter Hyperintensities: complex predictor of complex outcomes. J Am Heart Assoc. (2023) 12:e030351. doi: 10.1161/JAHA.123.030351, 37349890 PMC10356075

[9] Inzitari D Simoni M Pracucci G Poggesi A Basile AM Chabriat H . Risk of rapid global functional decline in elderly patients with severe cerebral age-related white matter changes: the LADIS study. Arch Intern Med. (2007) 167:81–8. doi: 10.1001/archinte.167.1.81 17210882

[10] Wardlaw JM Allerhand M Doubal FN Valdes HM Morris Z Gow AJ . Vascular risk factors, large-artery atheroma, and brain white matter Hyperintensities. Neurology. (2014) 82:312. doi: 10.1212/WNL.0000000000000312 PMC4001185 24623838

[11] Fazekas F Chawluk JB Alavi A Hurtig HI Zimmerman RA. MR signal abnormalities at 1.5 T in Alzheimer’s Dementia and Normal aging. AJR Am J Roentgenol. (1987) 149:351–6. doi: 10.2214/ajr.149.2.351, 3496763

[12] Desmarais P Gao AF Lanctôt K Rogaeva E Ramirez J Herrmann N . White matter Hyperintensities in autopsy-confirmed frontotemporal lobar degeneration and Alzheimer’s Disease. Alzheimer's Res Ther. (2021) 13:129 34256835 10.1186/s13195-021-00869-6 PMC8278704

[13] Lee S Viqar F Zimmerman ME Narkhede A Tosto G Benzinger TLS . White matter Hyperintensities are a Core feature of Alzheimer’s Disease: evidence from the dominantly inherited Alzheimer network. Ann Neurol. (2016) 79:929–39. doi: 10.1002/ana.24647, 27016429 PMC4884146

[14] Moghekar A Kraut M Elkins W Troncoso J Zonderman AB Resnick SM . Cerebral white matter Disease is associated with Alzheimer pathology in a prospective cohort. Alzheimers Dement. (2012) 8:S71–7. doi: 10.1016/j.jalz.2012.04.006 23021624 PMC3474974

[15] Wang Y-L Chen W Cai W-J Hao H Wei X Wang Z-T . Associations of white matter Hyperintensities with cognitive decline: a longitudinal study. JAD. (2020) 73:759–68. doi: 10.3233/JAD-191005 31839612

[16] Attems J Jellinger KA. The overlap between vascular Disease and Alzheimer’s Disease--lessons from pathology. BMC Med. (2014) 12:206. doi: 10.1186/s12916-014-0206-2 25385447 PMC4226890

[17] Chua XY Ho LTY Xiang P Chew WS Lam BWS Chen CP . Preclinical and clinical evidence for the involvement of sphingosine 1-phosphate signaling in the pathophysiology of vascular cognitive Impairment. NeuroMolecular Med. (2021) 23:47–67. doi: 10.1007/s12017-020-08632-0 33180310

[18] Kalaria RN. Neuropathological diagnosis of vascular cognitive Impairment and vascular Dementia with implications for Alzheimer’s Disease. Acta Neuropathol. (2016) 131:659–85. doi: 10.1007/s00401-016-1571-z 27062261 PMC4835512

[19] Dubois B Hampel H Feldman HH Scheltens P Aisen P Andrieu S . Preclinical Alzheimer’s Disease: definition, natural history, and diagnostic criteria. Alzheimers Dement. (2016) 12:292–323. doi: 10.1016/j.jalz.2016.02.002, 27012484 PMC6417794

[20] Liang L Liu W Zhong Y Guo T Ye C Ma T. Spatial-temporal interactions between white matter Hyperintensities and multiple pathologies across the Alzheimer’s Disease continuum. Alzheimers Dement. (2025) 21:e70098. doi: 10.1002/alz.70098 40302045 PMC12040729

[21] Newton P Tchounguen J Pettigrew C Lim C Lin Z Lu H . Regional white matter Hyperintensities and Alzheimer’s Disease biomarkers among older adults with Normal cognition and mild cognitive Impairment. JAD. (2023) 92:323–39. doi: 10.3233/JAD-220846 36744337 PMC10041440

[22] Dyck CH Van Dyck CH Swanson CJ Aisen P Bateman RJ Chen C . Lecanemab in early Alzheimer’s Disease. N Engl J Med. (2023) 388:9–21. doi: 10.1056/NEJMoa2212948, 36449413

[23] Lin J Wang D Lan L Fan Y. Multiple factors involved in the pathogenesis of white matter lesions. Biomed Res Int. (2017) 2017:2050. doi: 10.1155/2017/9372050 PMC5339523 28316994

[24] Mega Vascular Cognitive Impairment and Dementia (MEGAVCID) Consortium. A genome-wide association Meta-analysis of all-cause and vascular Dementia. Alzheimers Dement. (2024) 20:5973–95. doi: 10.1002/alz.14115 39046104 PMC11497727

[25] Shade LMP Katsumata Y Abner EL Aung KZ Claas SA Qiao Q . GWAS of multiple neuropathology Endophenotypes identifies new risk loci and provides insights into the genetic risk of Dementia. Nat Genet. (2024) 56:2407–21. doi: 10.1038/s41588-024-01939-9, 39379761 PMC11549054

[26] Brickman AM Schupf N Manly JJ Stern Y Luchsinger JA Provenzano FA . APOE ε4 and risk for Alzheimer’s Disease: do regionally distributed white matter Hyperintensities play a role? Alzheimers Dement. (2014) 10:155. doi: 10.1016/j.jalz.2014.07.155, 25304991 PMC4252241

[27] Rojas S Brugulat-Serrat A Bargalló N Minguillón C Tucholka A Falcon C . Higher prevalence of cerebral white matter Hyperintensities in homozygous APOE-ɛ4 allele carriers aged 45–75: results from the ALFA study. J. Cerebral Blood Flow Metabolism: Off. J. Int. Soc. Cerebral Blood Flow Metabolism. (2017) 38:250–61. doi: 10.1177/0271678X17707397 PMC5951016 28492093

[28] Morgen K Schneider M Frölich L Tost H Plichta MM Kölsch H . Apolipoprotein E-dependent load of white matter Hyperintensities in Alzheimer’s Disease: a voxel-based lesion mapping study. Alzheimer's Res Ther. (2015) 7:1–14. doi: 10.1186/s13195-015-0111-8 25984242 PMC4432954

[29] Persyn E Hanscombe KB Howson JMM Lewis CM Traylor M Markus HS. Genome-wide association study of MRI markers of cerebral small vessel Disease in 42,310 participants. Nat Commun. (2020) 11:1–12. doi: 10.1038/s41467-020-15932-3 32358547 PMC7195435

[30] Verhaaren BFJ Debette S Bis JC Smith JA Kamran Ikram M Adams HH . Multi-ethnic genome-wide association study of cerebral white matter Hyperintensities on MRI. Circ Cardiovasc Genet. (2015) 8:398. doi: 10.1161/CIRCGENETICS.114.000858, 25663218 PMC4427240

[31] Fornage M Beecham AH. The emerging genetic landscape of cerebral white matter Hyperintensities. Neurology. (2019) 92:6936. doi: 10.1212/WNL.0000000000006936 30659142

[32] Rutten-Jacobs LCA Tozer DJ Duering M Malik R Dichgans M Markus HS . Genetic study of white matter integrity in UK biobank (N=8448) and the overlap with stroke, depression, and Dementia. Stroke. (2018) 49:1340–7. doi: 10.1161/STROKEAHA.118.020811, 29752348 PMC5976227

[33] Armstrong NJ Mather KA Sargurupremraj M Knol MJ Malik R Satizabal CL . Common genetic variation indicates separate causes for periventricular and deep white matter Hyperintensities. Stroke. (2020) 51:2111–21. doi: 10.1161/STROKEAHA.119.027544 32517579 PMC7365038

[34] Molinuevo JL Gramunt N Gispert JD Fauria K Esteller M Minguillon C . The ALFA project: a research platform to identify early pathophysiological features of Alzheimer’s Disease. Alzheimer’s Dementia Transl. Res. Clin. Interv. (2016) 2:82–92. doi: 10.1016/j.trci.2016.02.003 PMC5644283 29067295

[35] Brugulat-Serrat A Rojas S Bargalló N Conesa G Minguillón C Fauria K . Incidental findings on brain MRI of cognitively Normal first-degree descendants of patients with Alzheimer’s Disease: a cross-sectional analysis from the ALFA (Alzheimer and Families) project. BMJ Open. (2017) 7:215. doi: 10.1136/bmjopen-2016-013215, 28341686 PMC5372150

[36] Brugulat-Serrat A Salvadó G Sudre CH Grau-Rivera O Suárez-Calvet M Falcon C . Patterns of white matter Hyperintensities associated with cognition in middle-aged cognitively healthy individuals. Brain Imaging Behav. (2020) 14:2012–23. doi: 10.1007/s11682-019-00151-2 31278650 PMC7572336

[37] Elosua R Garcia M Aguilar A Molina L Covas MI Marrugat J. Validation of the Minnesota leisure time physical activity questionnaire in Spanish women. Investigators of the MARATDON group. Med Sci Sports Exerc. (2000) 32:11. doi: 10.1097/00005768-200008000-00011 10949009

[38] Kivipelto M Ngandu T Laatikainen T Winblad B Soininen H Tuomilehto J. Risk score for the prediction of Dementia risk in 20 years among middle aged people: a longitudinal, population-based study. Lancet Neurol. (2006) 5:735–41. doi: 10.1016/S1474-4422(06)70537-3, 16914401

[39] Peña-Casanova J Gramunt-Fombuena N Quiñones-Ubeda S Sánchez-Benavides G Aguilar M Badenes D . Spanish multicenter normative studies (NEURONORMA project): norms for the Rey-Osterrieth complex Figure (copy and memory), and free and cued selective reminding test. Arch. Clin. Neuropsychol. Off. J. Nat. Acad. Neuropsychol. (2009) 24:371–93. doi: 10.1093/arclin/acp041, 19661107

[40] Brugulat-Serrat A Cañas-Martínez A Canals-Gispert L Marne P Gramunt N Milà-Alomà M . Enhancing the sensitivity of memory tests: reference data for the free and cued selective reminding test and the logical memory task from cognitively healthy subjects with Normal Alzheimer’s Disease cerebrospinal fluid biomarker levels. JAD. (2021) 84:119–28. doi: 10.3233/JAD-210640, 34569957 PMC8609690

[41] WAIS-IV (2012). escala de inteligencia de Wechsler para adultos-IV: [Test Psicología].

[42] Das S Forer L Schönherr S Sidore C Locke AE Kwong A . Next-generation genotype imputation service and methods. Nat Genet. (2016) 48:1284–7. doi: 10.1038/ng.3656, 27571263 PMC5157836

[43] Vilor-Tejedor N Genius P Rodríguez-Fernández B Minguillón C Sadeghi I González-Escalante A . Genetic characterization of the ALFA study: uncovering genetic profiles in the Alzheimer’s continuum. Alzheimer’s Dementia J. (2023) 20:1703–15. doi: 10.1002/alz.13537 PMC10984507 38088508

[44] Choi SW O’Reilly PF. PRSice-2: polygenic risk score software for biobank-scale data. GigaScience. (2019) 8:giz082. doi: 10.1093/gigascience/giz082, 31307061 PMC6629542

[45] Sudre CH Cardoso MJ Bouvy WH Biessels GJ Barnes J Ourselin S. Bayesian model selection for pathological Neuroimaging data applied to white matter lesion segmentation. IEEE Trans Med Imaging. (2015) 34:9072. doi: 10.1109/TMI.2015.2419072 25850086

[46] Ni L Zhou F Qing Z Zhang X Li M Zhu B . The asymmetry of white matter Hyperintensity burden between hemispheres is associated with intracranial atherosclerotic plaque enhancement grade. Front Aging Neurosci. (2020) 12:163. doi: 10.3389/fnagi.2020.00163 32655391 PMC7324557

[47] Schmidt R Enzinger C Ropele S Schmidt H Fazekas F. Progression of cerebral white matter lesions: 6-year results of the Austrian stroke prevention study. Lancet. (2003) 361:2046–8. doi: 10.1016/s0140-6736(03)13616-1 12814718

[48] Schmidt R Schmidt H Haybaeck J Loitfelder M Weis S Cavalieri M . Heterogeneity in age-related white matter changes. Acta Neuropathol. (2011) 122:171–85. doi: 10.1007/s00401-011-0851-x, 21706175

[49] Friedewald WT Levy RI Fredrickson DS. Estimation of the concentration of low-density lipoprotein cholesterol in plasma, without use of the preparative ultracentrifuge. Clin Chem. (1972) 18:499–502. 4337382

[50] Expert Panel on Detection, Evaluation, and Treatment of High Blood Cholesterol in Adults. Executive summary of the third report of the National Cholesterol Education Program (NCEP) expert panel on detection, evaluation, and treatment of high blood cholesterol in adults (adult treatment panel III). JAMA. (2001) 285:2486–97. doi: 10.1001/jama.285.19.2486 11368702

[51] Verma AK Sun J-L Hernandez A Teerlink JR Schulte PJ Ezekowitz J . Rate pressure product and the components of heart rate and systolic blood pressure in hospitalized heart failure patients with preserved ejection fraction: insights from ASCEND-HF. Clin Cardiol. (2018) 41:945–52. doi: 10.1002/clc.22981, 29781109 PMC6103846

[52] Protogerou AD Vlachopoulos C Thomas F Zhang Y Pannier B Blacher J . Longitudinal changes in mean and pulse pressure, and all-cause mortality: data from 71,629 untreated normotensive individuals. Am J Hypertens. (2017) 30:1093–9. doi: 10.1093/ajh/hpx110 28655182

[53] Liu Q Li C Wanga V Shepherd BE. Covariate-adjusted spearman’s rank correlation with probability-scale residuals. Biometrics. (2018) 74:595–605. doi: 10.1111/biom.12812, 29131931 PMC5949238

[54] Shepherd BE Li C Liu Q. Probability-scale residuals for continuous, discrete, and censored data. Can. J. Stat. (2016) 44:463–79. doi: 10.1002/cjs.11302, 28348453 PMC5364820

[55] Tesi N van der Lee S Hulsman M Holstege H Reinders MJT. snpXplorer: a web application to explore human SNP-associations and annotate SNP-sets. Nucleic Acids Res. (2021) 49:W603–12. doi: 10.1093/nar/gkab410, 34048563 PMC8262737

[56] Wu T Hu E Xu S Chen M Guo P Dai Z . clusterProfiler 4.0: a universal enrichment tool for interpreting omics data. Innovation. (2021) 2:100141. doi: 10.1016/j.xinn.2021.100141, 34557778 PMC8454663

[57] Peterson R. Finding optimal normalizing transformations via best normalize. R J. (2021) 13:310. doi: 10.32614/RJ-2021-041

[58] Favari E Chroni A Tietge UJF Zanotti I Escolà-Gil JC Bernini F. Cholesterol efflux and reverse cholesterol transport. Handb Exp Pharmacol. (2015) 224:181–206. doi: 10.1007/978-3-319-09665-0_4 25522988

[59] Georgakis MK Malik R Anderson CD Parhofer KG Hopewell JC Dichgans M. Genetic determinants of blood lipids and cerebral small vessel Disease: role of high-density lipoprotein cholesterol. Brain J. Neurol. (2020) 143:597–610. doi: 10.1093/brain/awz413 PMC7009571 31968102

[60] Singh K Rohatgi A. Examining the paradox of high high-density lipoprotein and elevated cardiovascular risk. J Thorac Dis. (2018) 10:109–12. doi: 10.21037/jtd.2017.12.97 29600034 PMC5863140

[61] Collins R Armitage J Parish S Sleight P Peto R. Effects of cholesterol-lowering with simvastatin on stroke and other major vascular events in 20536 people with cerebrovascular Disease or other high-risk conditions. Lancet. (2004) 363:757–67. doi: 10.1016/S0140-6736(04)15690-0 15016485

[62] US Preventive Services Task Force Mangione CM Barry MJ Nicholson WK Cabana M Chelmow D . Statin use for the primary prevention of cardiovascular Disease in adults: US preventive services task force recommendation statement. JAMA. (2022) 328:746–53. doi: 10.1001/jama.2022.13044, 35997723

[63] Wartolowska KA Webb AJS. Midlife blood pressure is associated with the severity of white matter Hyperintensities: analysis of the UK biobank cohort study. Eur Heart J. (2021a) 42:750–7. doi: 10.1093/eurheartj/ehaa756, 33238300 PMC7882359

[64] Kaul M Rubinstein I. Population-based magnetic resonance imaging: earlier detection of hypertensive cerebral small vessel Disease? Hypertension. (2021) 78:540–2. doi: 10.1161/HYPERTENSIONAHA.121.17606, 34232679 PMC8284836

[65] Wartolowska KA Webb AJS. Blood pressure determinants of cerebral white matter Hyperintensities and microstructural injury: UK biobank cohort study. Hypertension. (2021b) 78:532–9. 34058855 10.1161/HYPERTENSIONAHA.121.17403 PMC8260341

[66] Jacków-Nowicka J Podgórski P Bladowska J Szcześniak D Rymaszewska J Zatońska K . The impact of common epidemiological factors on gray and white matter volumes in magnetic resonance imaging–is prevention of brain degeneration possible? Front Neurol. (2021) 12:633619. doi: 10.3389/fneur.2021.633619 34326804 PMC8315783

[67] Soljanlahti S Autti T Lauerma K Raininko R Keto P Turtola H . Familial hypercholesterolemia patients treated with statins at no increased risk for intracranial vascular lesions despite increased cholesterol burden and extracranial atherosclerosis. Stroke J. Cereb. Circ. (2005) 36:1572–74. doi: 10.1161/01.STR.0000169920.64180.fa 15933262

[68] Rodriguez FS Lampe L Gaebler M Beyer F Baber R Burkhardt R . Differences in white matter Hyperintensities in socioeconomically deprived groups: results of the population-based LIFE adult study. IPA. (2023) 36:785–98. doi: 10.1017/S104161022300025X 37039457

[69] Mulugeta A Navale SS Lumsden AL Llewellyn DJ Hyppönen E. Healthy lifestyle, genetic risk and brain health: a gene-environment interaction study in the UK biobank. Nutrients. (2022) 14:3907. doi: 10.3390/nu14193907, 36235559 PMC9570683

[70] Launer LJ. Epidemiology of white matter lesions. TMRI. (2004) 15:38. doi: 10.1097/01.rmr.0000168216.98338.8 16041288

[71] Sargurupremraj M Suzuki H Jian X Sarnowski C Evans TE Bis JC . Cerebral small vessel Disease genomics and its implications across the lifespan. Nat Commun. (2020) 11:1–18. doi: 10.1038/s41467-020-19111-2 33293549 PMC7722866

[72] Lohner V Pehlivan G Sanroma G Miloschewski A Schirmer MD Stöcker T . Relation between sex, menopause, and white matter Hyperintensities: the Rhineland study. Neurology. (2022) 99:e935–43. doi: 10.1212/WNL.0000000000200782, 35768207 PMC9502737

