# Supplementary material for: Polygenic risk for white matter hyperintensities is associated with early cerebrovascular events partly through hemodynamic measures in cognitively unimpaired middle-aged and older adults with low cardiovascular risk
Source: Front Neurol. 2026 Jan 5;16:1667424. doi: 10.3389/fneur.2025.1667424 (PMC12812530; doi:10.3389/fneur.2025.1667424)
Supplement: Supplementary file 1 [file Data_Sheet_1.pdf]

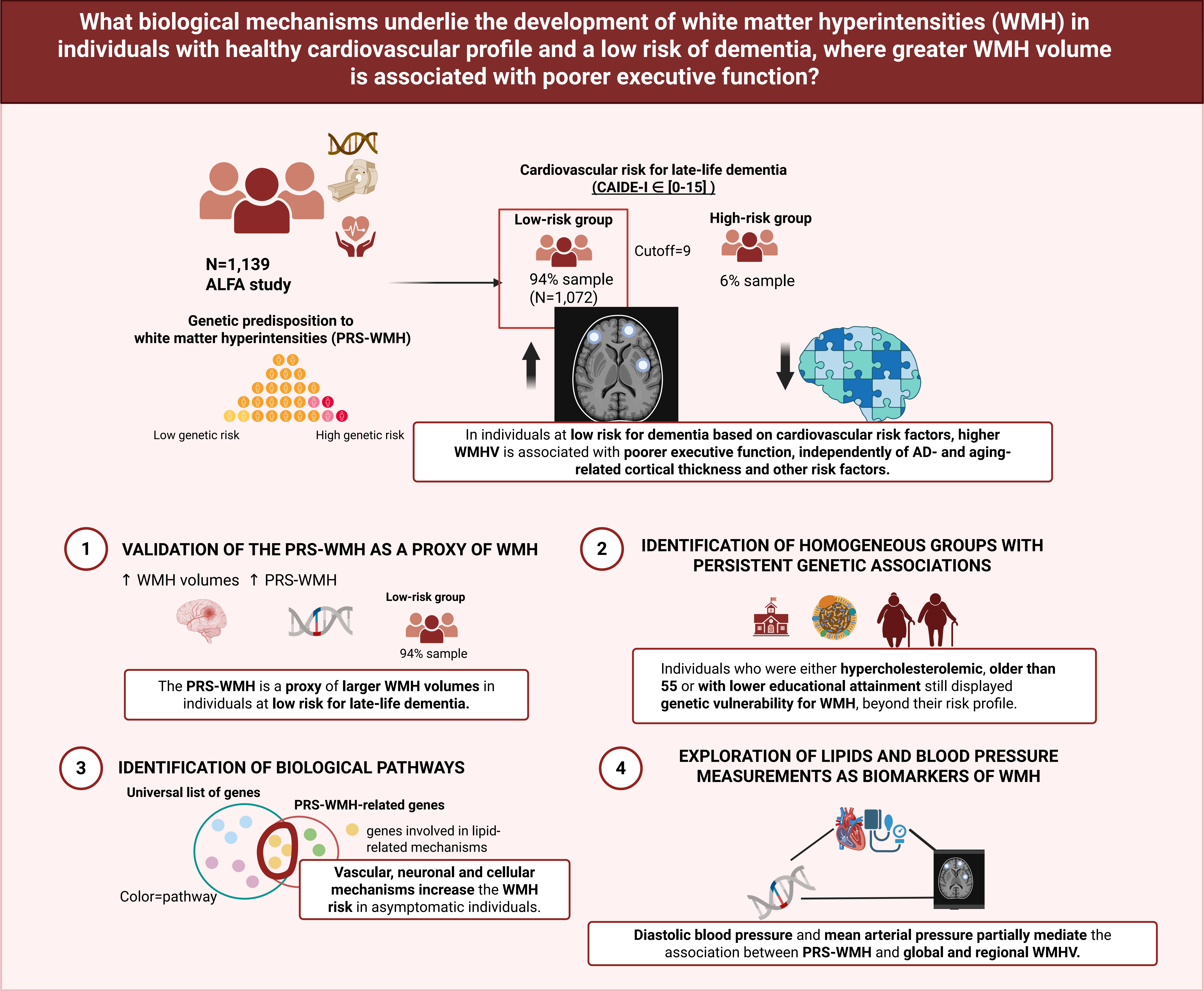

Central Illustration. Graphical abstract of the study that aimed to explore the biological mechanisms driving cerebrovascular disease in asymptomatic middle-aged individuals at low cardiovascular risk for late-life dementia.
