# Supplementary material for: Polygenic risk for white matter hyperintensities is associated with early cerebrovascular events partly through hemodynamic measures in cognitively unimpaired middle-aged and older adults with low cardiovascular risk
Source: Front Neurol. 2026 Jan 5;16:1667424. doi: 10.3389/fneur.2025.1667424 (PMC12812530; doi:10.3389/fneur.2025.1667424)
Supplement: Supplementary file 2 [file Data_Sheet_2.pdf]

Supplementary Figures

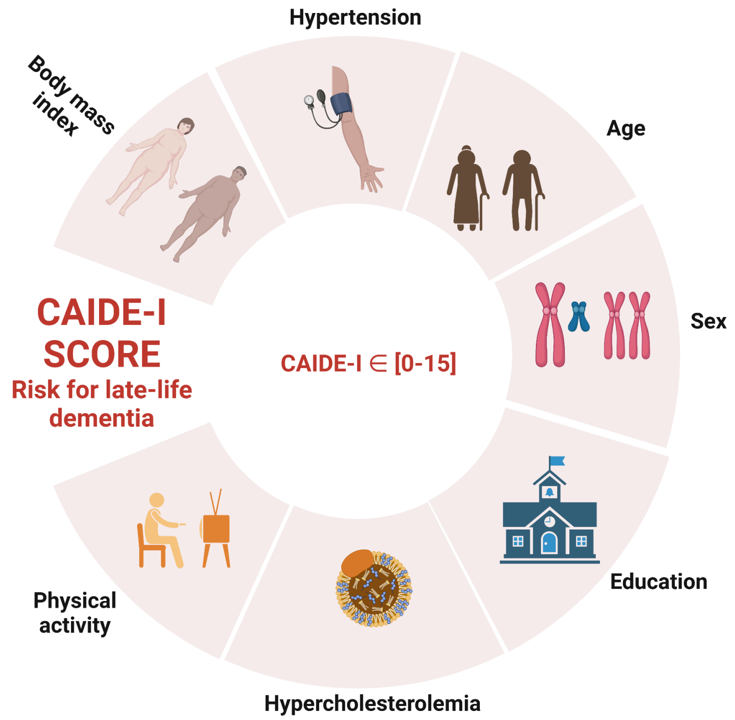

Supplementary Figure 1. Variables included in the predicted 20-years risk of dementia based on cardiovascular risk factors (CAIDE-I). *Legend: the probability of dementia late in life according to the CAIDE-I risk score categories are (i) CAIDE-I [0-5]: 1%; (ii) CAIDE-I [6-7] : 1.9%; (iii) CAIDE-I [8-9]: 4.2%; (iv) CAIDE-I [10-11]: 7.4% and CAIDE-I [12-15]: 16.4% (Kivipelto et al., 2006).*

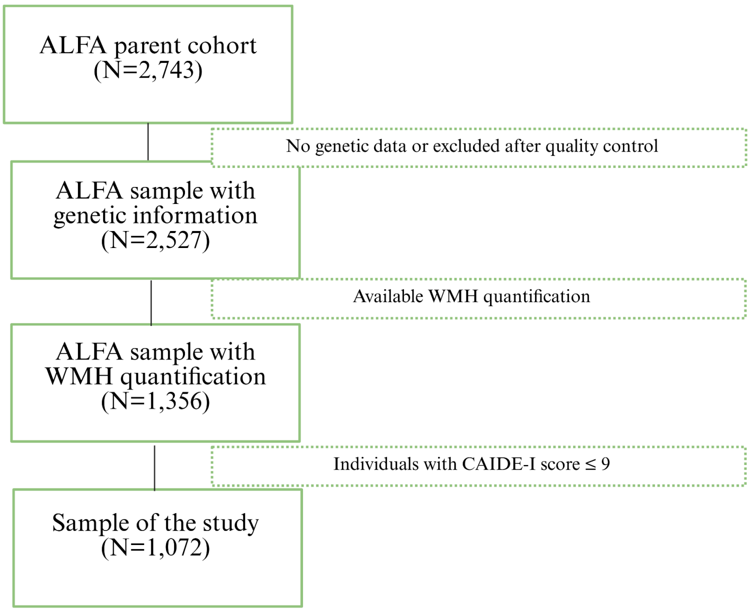

Supplementary Figure 2. Flowchart of the sample of the study. *Legend: ALFA (Alzheimer's and Families), WMH (White matter hyperintensities). All individuals had available quantification of WMHV, genetic data and predicted 20-years risk of dementia based on cardiovascular risk factors.*

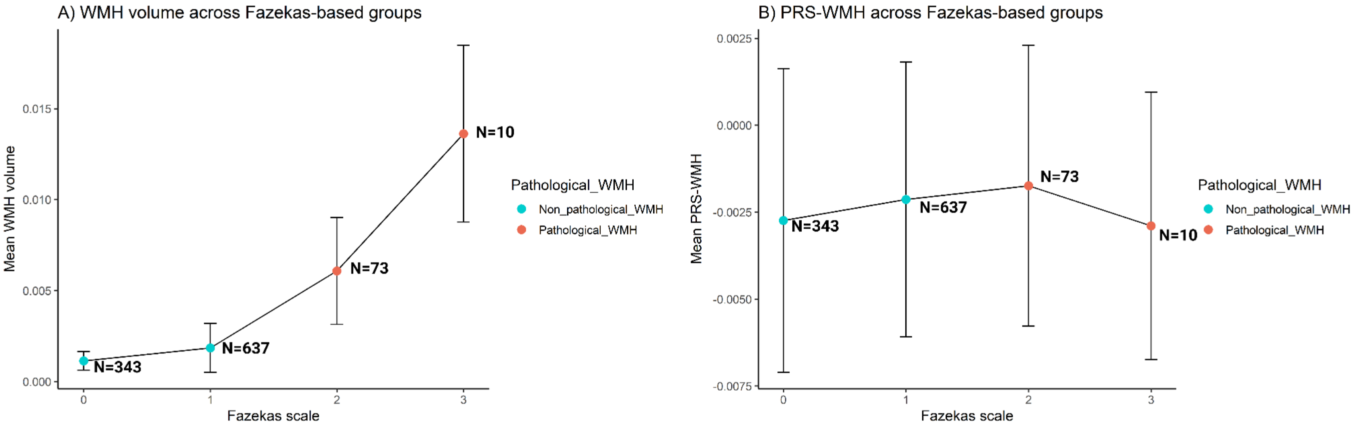

Supplementary Figure 3. Mean value and standard error of global WMHV along the WMH severity continuum based on the Fazekas scale. *Global WMHV were adjusted for TIV and expressed per 100mm³.*

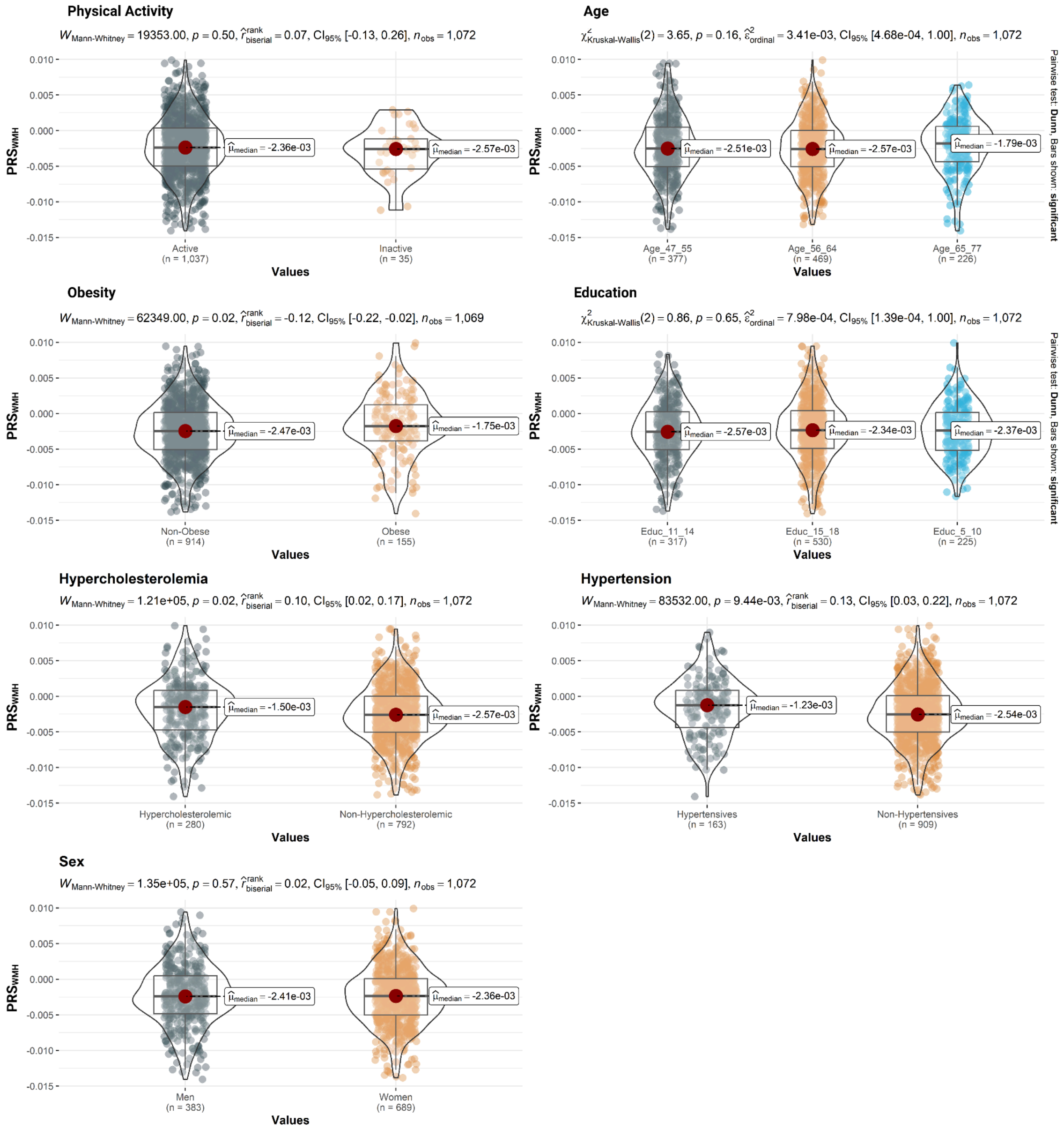

Supplementary Figure 4. Violin plots depicting the distribution of the PRS_{WMH} across different cardiovascular and lifestyle risk factors. *Footnote: each subplot compares PRS values between groups categorized by physical activity, age group, BMI classification, education level, hypercholesterolemia status, hypertension status, and sex. The red squares represent median values, and statistical comparisons were performed using either the Mann-Whitney U test or Kruskal-Wallis test, as indicated.*

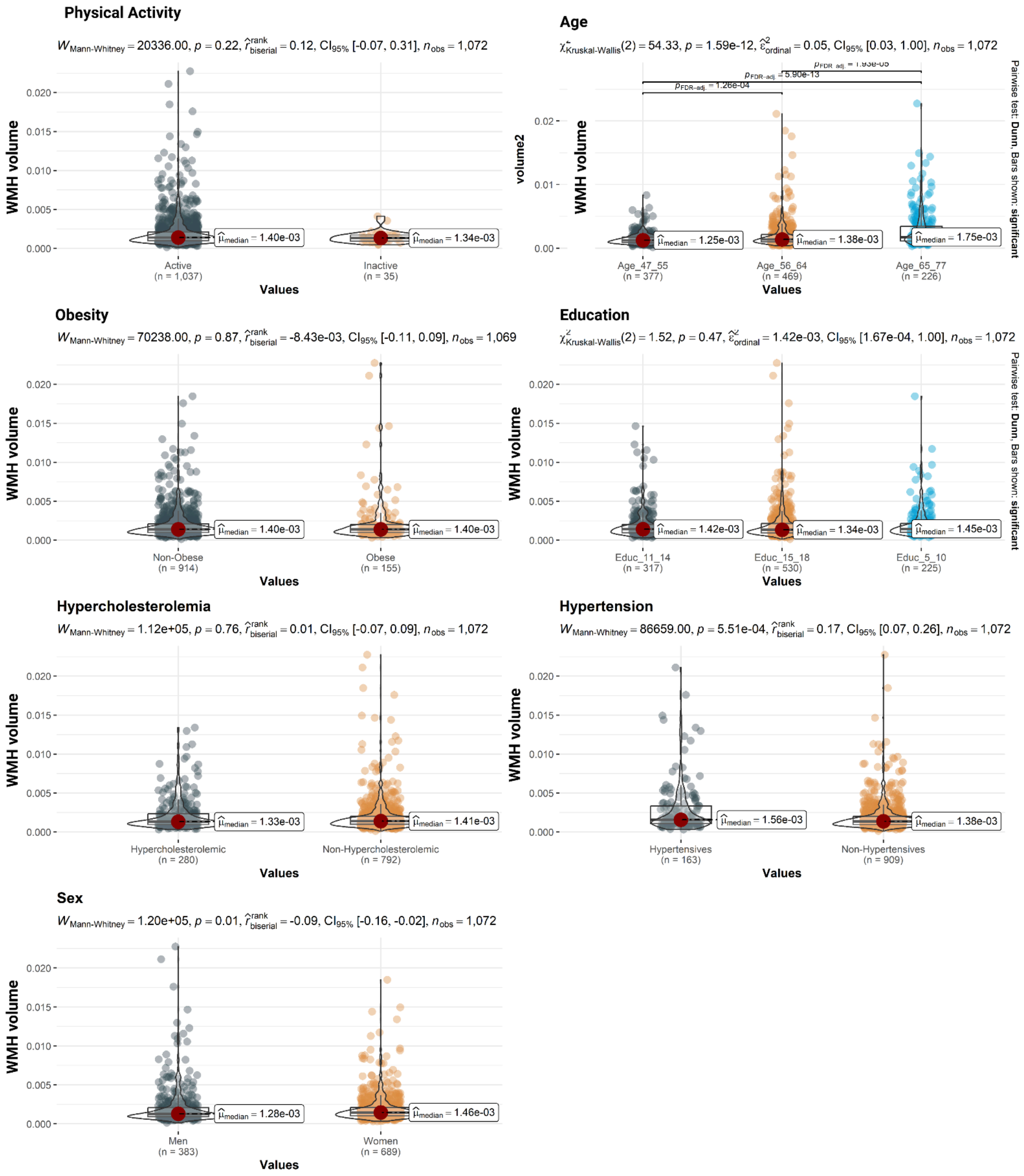

Supplementary Figure 5. Violin plots depicting the distribution of global WMH volume across different cardiovascular and lifestyle risk factors.

Footnote: each subplot compares WMH volume between groups categorized by physical activity, age group, BMI classification, education level, hypercholesterolemia status, hypertension status, and sex. The red squares represent median values, and statistical comparisons were performed using either the Mann-Whitney U test or Kruskal-Wallis test, as indicated.

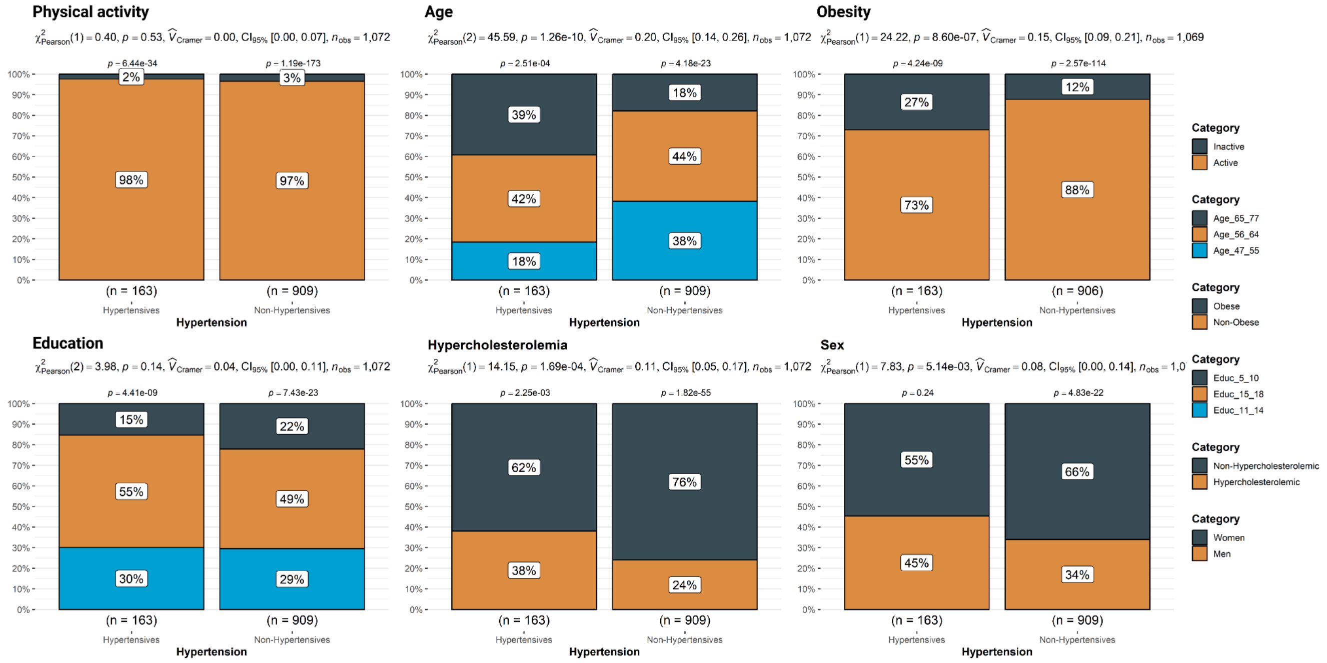

Supplementary Figure 6. Stacked bar plots illustrating the distribution of various cardiovascular and demographic risk factors stratified by hypertension status. *Footnote: each panel represents the proportion of individuals within each category for hypertensive (left bars) and non-hypertensive (right bars) groups. Chi-square tests were performed to assess associations between hypertension and each risk factor, with p-values and Cramér's V effect sizes displayed.*

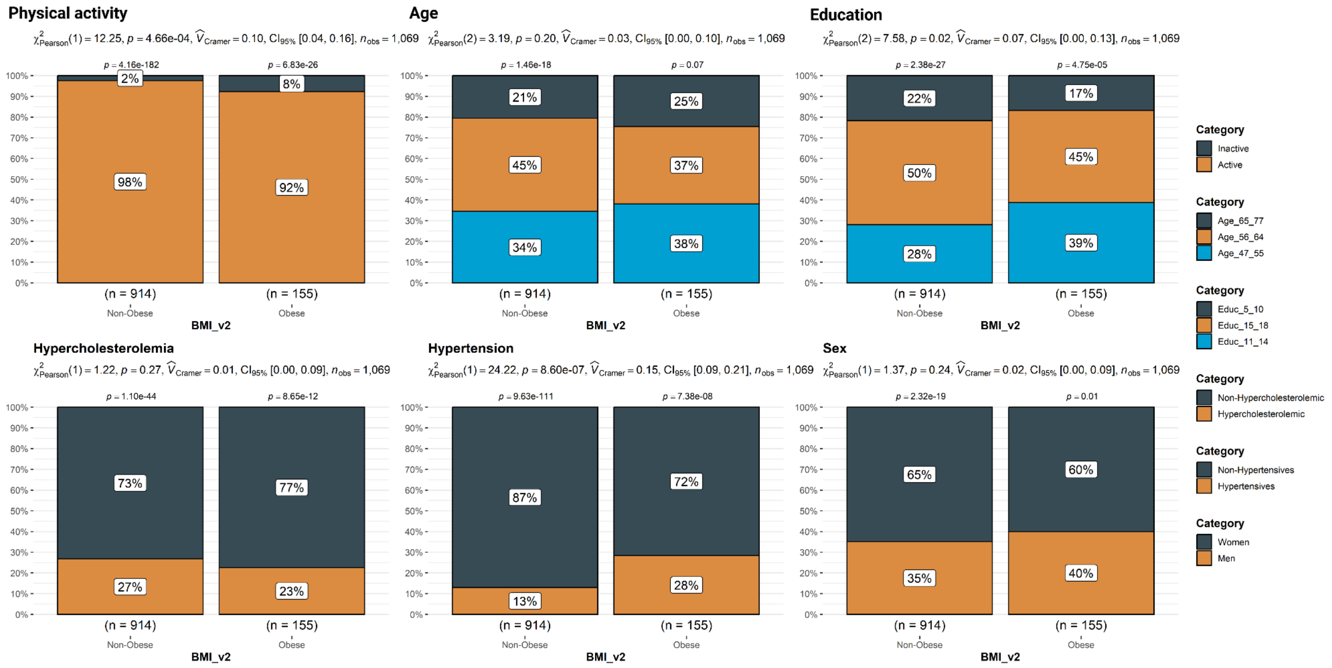

Supplementary Figure 7. Stacked bar plots illustrating the distribution of various cardiovascular and demographic risk factors stratified by BMI status (obesity). *Footnote: each panel represents the proportion of individuals within each category for non-obese (left bars) and obese (right bars) groups. Chi-square tests were performed to assess associations between hypertension and each risk factor, with p-values and Cramér's V effect sizes displayed.*

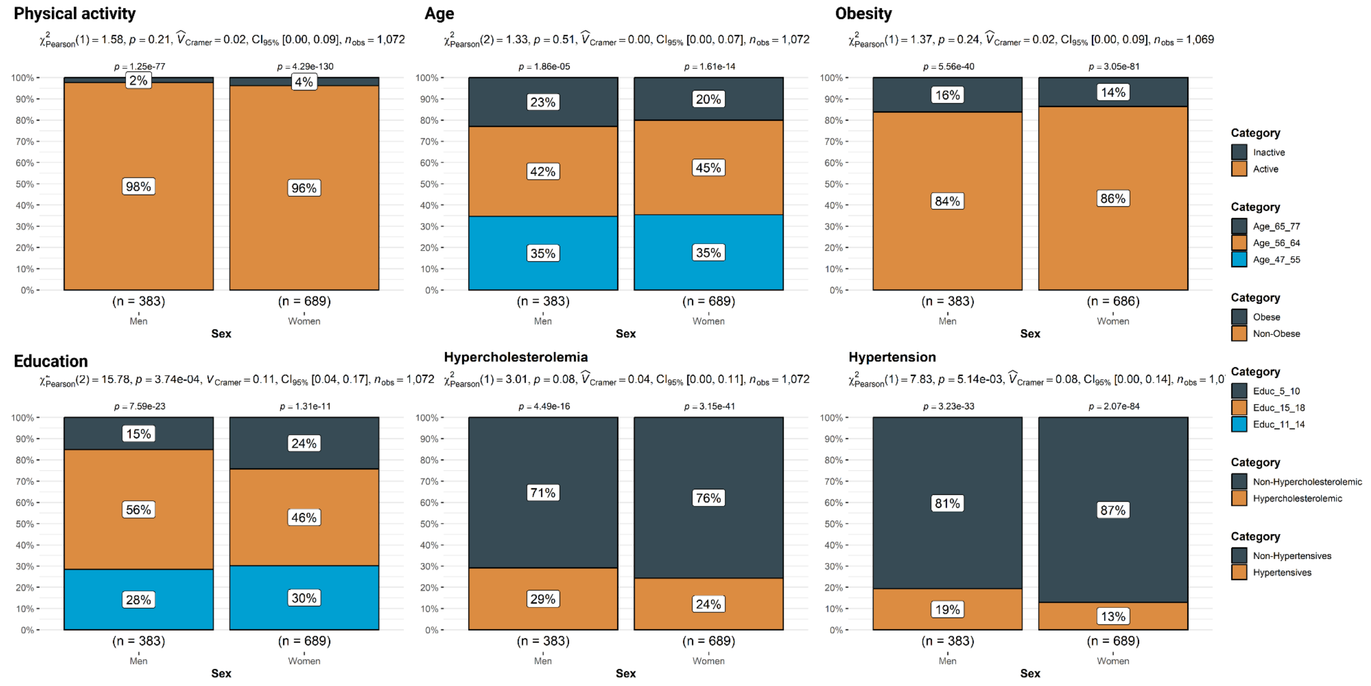

Supplementary Figure 8. Stacked bar plots illustrating the distribution of various cardiovascular and demographic risk factors stratified by sex.
Footnote: each panel represents the proportion of individuals within each category for men (left bars) and women (right bars) groups. Chi-square tests were performed to assess associations between hypertension and each risk factor, with p-values and Cramér's V effect sizes displayed.

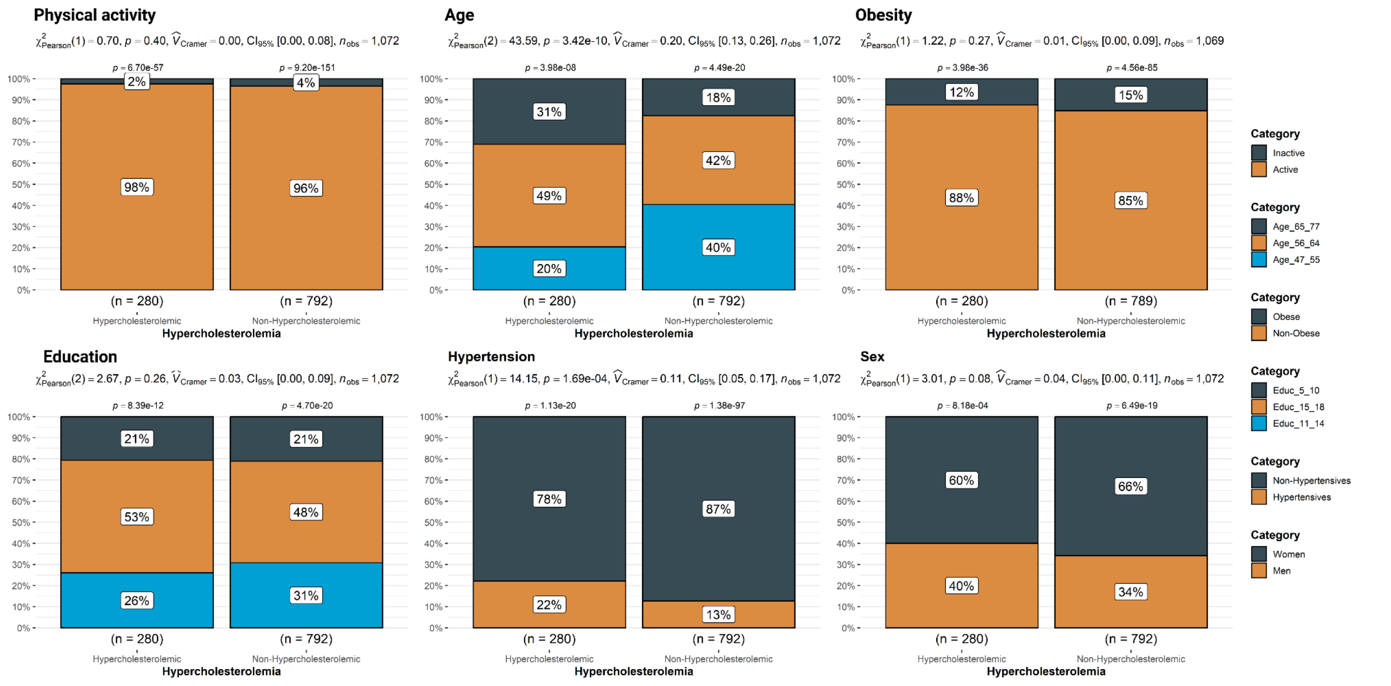

Supplementary Figure 9. Stacked bar plots illustrating the distribution of various cardiovascular and demographic risk factors stratified by hypercholesterolemia status.
Footnote: each panel represents the proportion of individuals within each category for hypercholesterolemic (left bars) and non-hypercholesterolemic (right bars) groups. Chi-square tests were performed to assess associations between hypertension and each risk factor, with p-values and Cramér's V effect sizes displayed.

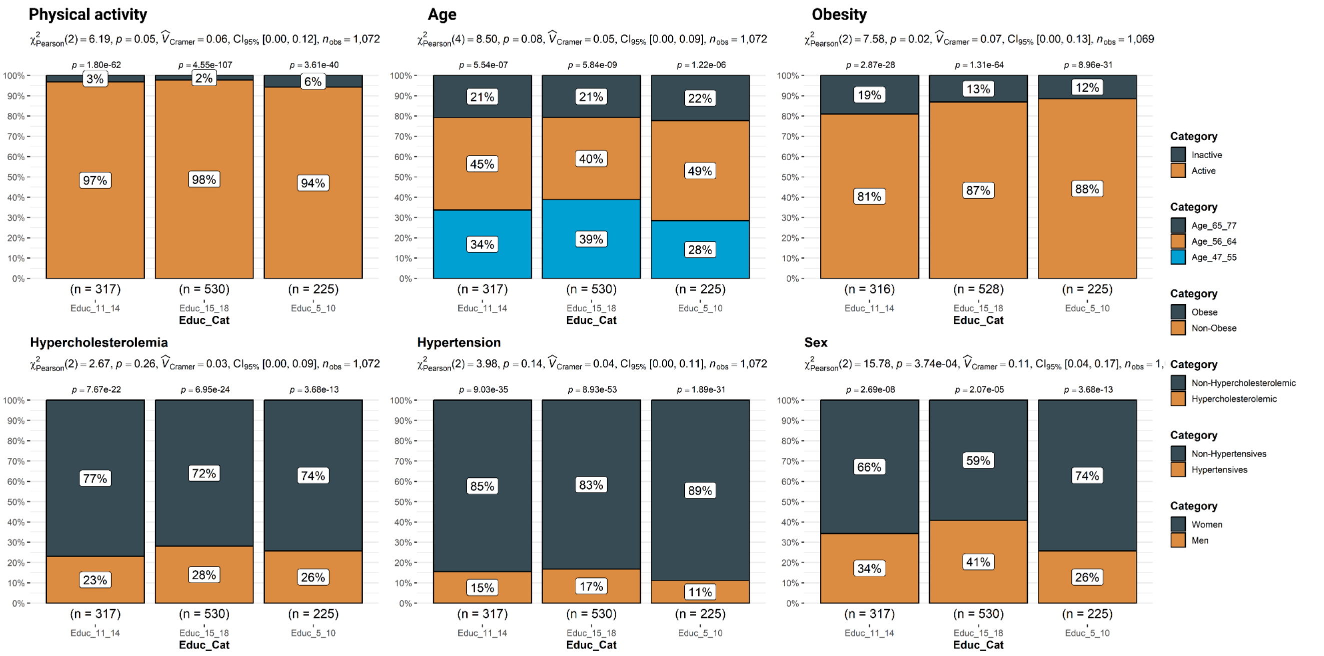

Supplementary Figure 10. Stacked bar plots illustrating the distribution of various cardiovascular and demographic risk factors stratified by education group.
Footnote: each panel represents the proportion of individuals within each category for 11-14 years education (left bars), 15-18 years of education (mid bars) and 5-10 years of education (right bars) groups. Chi-square tests were performed to assess associations between hypertension and each risk factor, with p-values and Cramér's V effect sizes displayed.

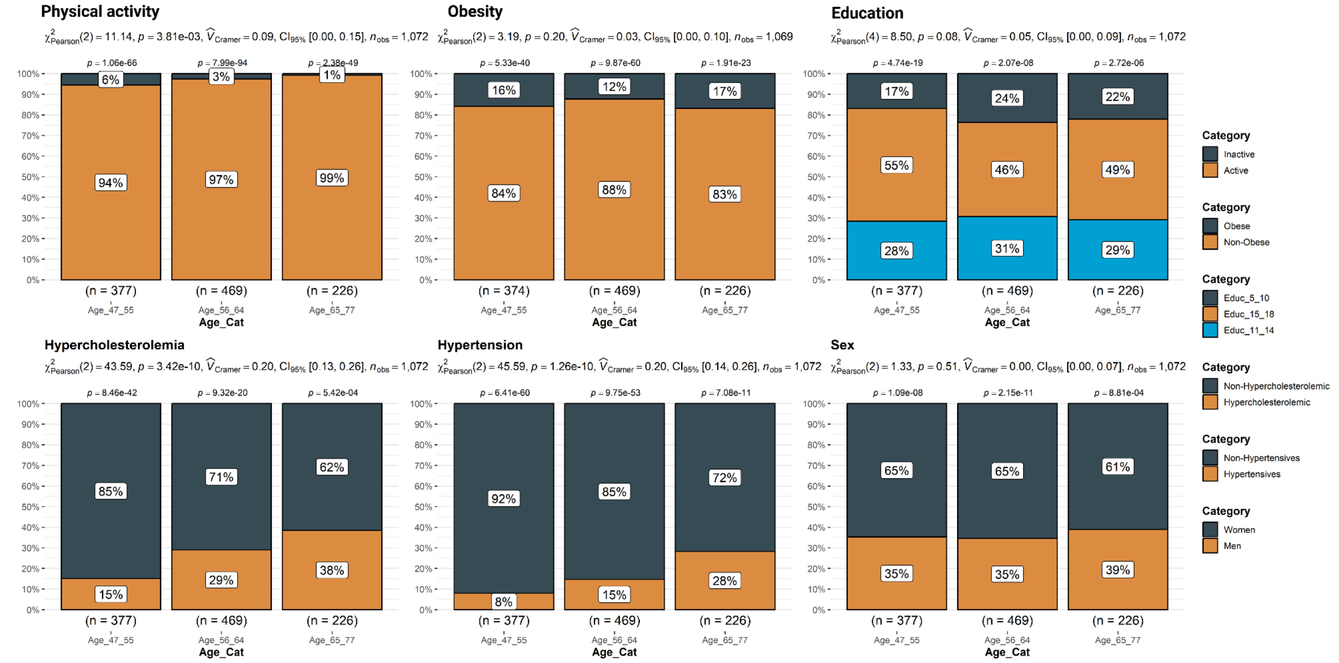

Supplementary Figure 11. Stacked bar plots illustrating the distribution of various cardiovascular and demographic risk factors stratified by age groups.
Footnote: each panel represents the proportion of individuals within each category for age 47-55 (left bars), age 56-64 (mid bars) and age 65-77 (right bars). Chi-square tests were performed to assess associations between hypertension and each risk factor, with p-values and Cramér's V effect sizes displayed.

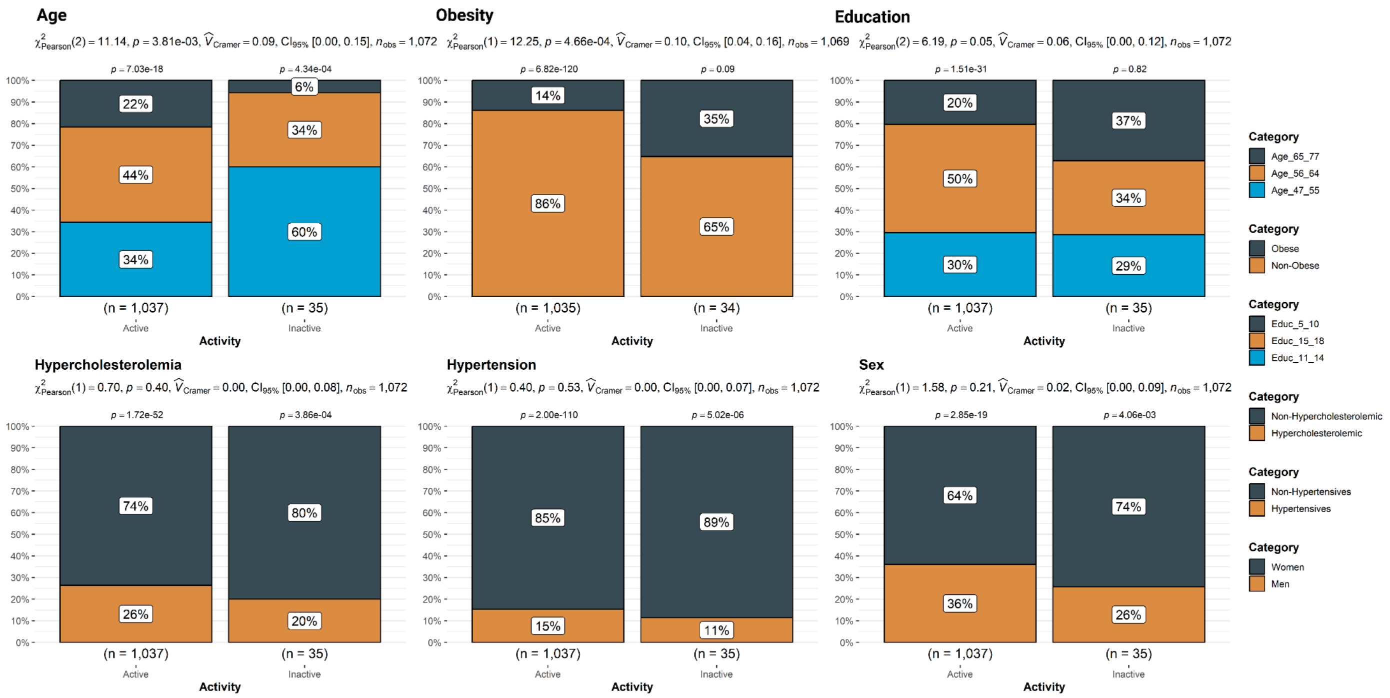

Supplementary Figure 12. Stacked bar plots illustrating the distribution of various cardiovascular and demographic risk factors stratified by physical activity status. *Footnote: each panel represents the proportion of individuals within each category for active (left bars) and inactive (right bars) groups. Chi-square tests were performed to assess associations between hypertension and each risk factor, with p-values and Cramér's V effect sizes displayed.*

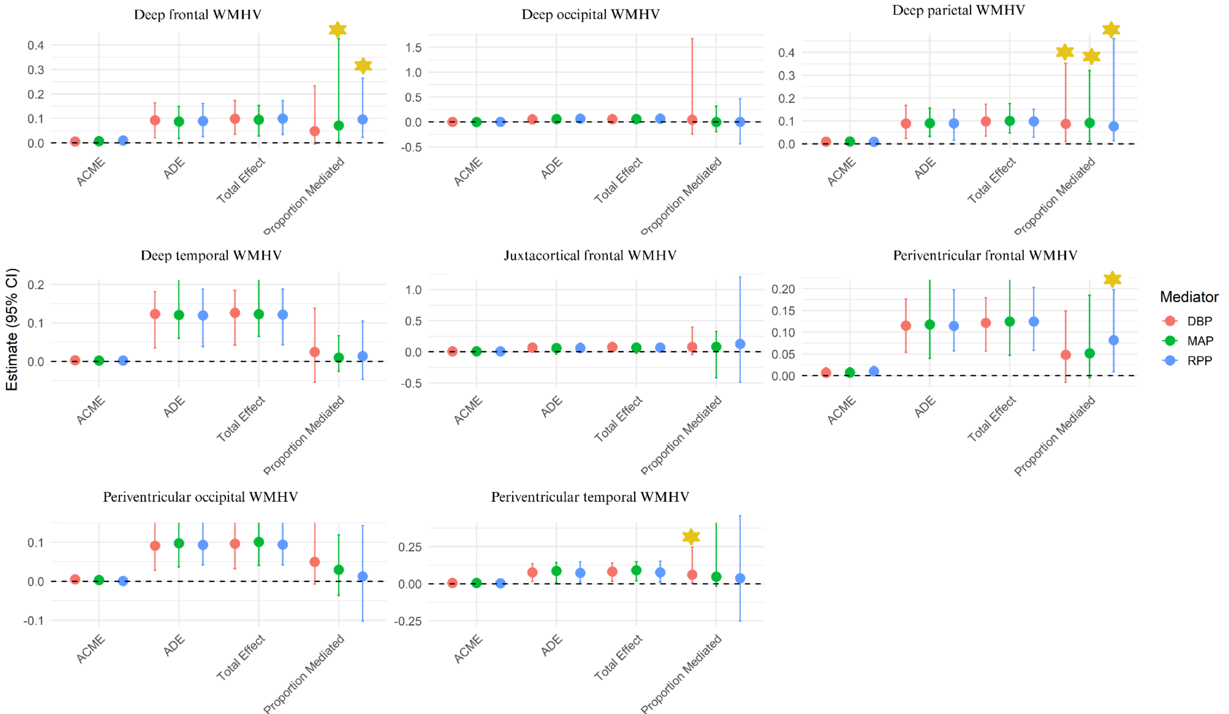

Supplementary Figure 13. Mediation analysis results exploring the mediator role of DBP, MAP and PP in the association between the PRS of WMH and WMH in non-hypertensive individuals from the study sample.
Footnote. Confidence intervals were reported (α=0.05, 95%CI). Significant mediated pathways were highlighted with a yellow star symbol.
Legend: ACME (Average Causal Mediation Effect), ADE (Average Direct Effect), DBP (Diastolic blood pressure), MAP (Mean arterial pressure), PP (Pulse pressure).
