# Supplementary material for: Polygenic risk for white matter hyperintensities is associated with early cerebrovascular events partly through hemodynamic measures in cognitively unimpaired middle-aged and older adults with low cardiovascular risk
Source: Front Neurol. 2026 Jan 5;16:1667424. doi: 10.3389/fneur.2025.1667424 (PMC12812530; doi:10.3389/fneur.2025.1667424)
Supplement: Supplementary file 3 [file Data_Sheet_3.pdf]

Supplementary Results

WMH volume is associated with poorer executive function in individuals at low risk to develop dementia late in life based on traditional cardiovascular risk factors

A total of 758 individuals had available information on cognitive function. In this study, we analysed four FCSRT outcomes corresponding to immediate free and total recall, as well as delayed free and total recall. These scores were z-scored and averaged in an episodic memory (EM) composite. Similarly, two subtests of the WAIS-IV (coding subset and visual puzzles) were z-scored and averaged into an executive functioning (EF) composite [Figure 1A]. Cognitive composites were z-scored in the sample [Figure 1B].

A. **B.**

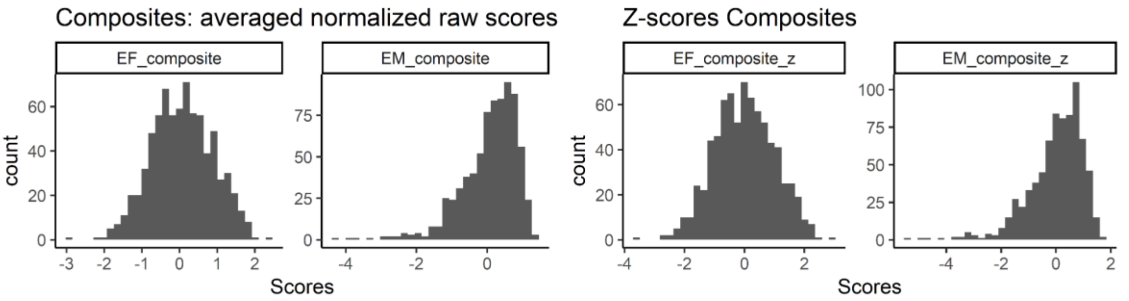

Figure 1. (A) Distribution of the averaged z-scores of the independent tests included in the executive functioning and episodic memory composite as well as (B) distribution of the z-scored composite scores.

Linear regression models were used to explore the association between WMHV and both EM and EF. WMHV was included as the main predictor, after best normalizing transformation (*bestNormalize R package)*, and base models were adjusted for age, sex and years of education. A non-significant association was found between WMHV and the EM composite. However, a significant association was found between larger WMHV and poorer EF (β = -0.0641 [-0.125, -0.003], p-value=0.0382) [Figure 2]. Sensitivity analyses, after adjusting for the potential confounding effect of hypertension (as a main risk factor for WMH), CAIDE-I score (risk for dementia based on cardiovascular risk factors), *APOE-ε*4 carriership (associated with cognition and WMHV), hippocampal volume (as a hallmark of

neurodegeneration), aging and AD signatures of Dickerson (as patterns of cortical thickness in AD and age-vulnerable regions), showed that WMHV was a significant predictor of poorer EF, independently of the effect of the aforementioned variables [Figure 2]. The full model (Model 7), which included all the variables, was the only one in which WMHV were not statistically significantly associated with EF scores.

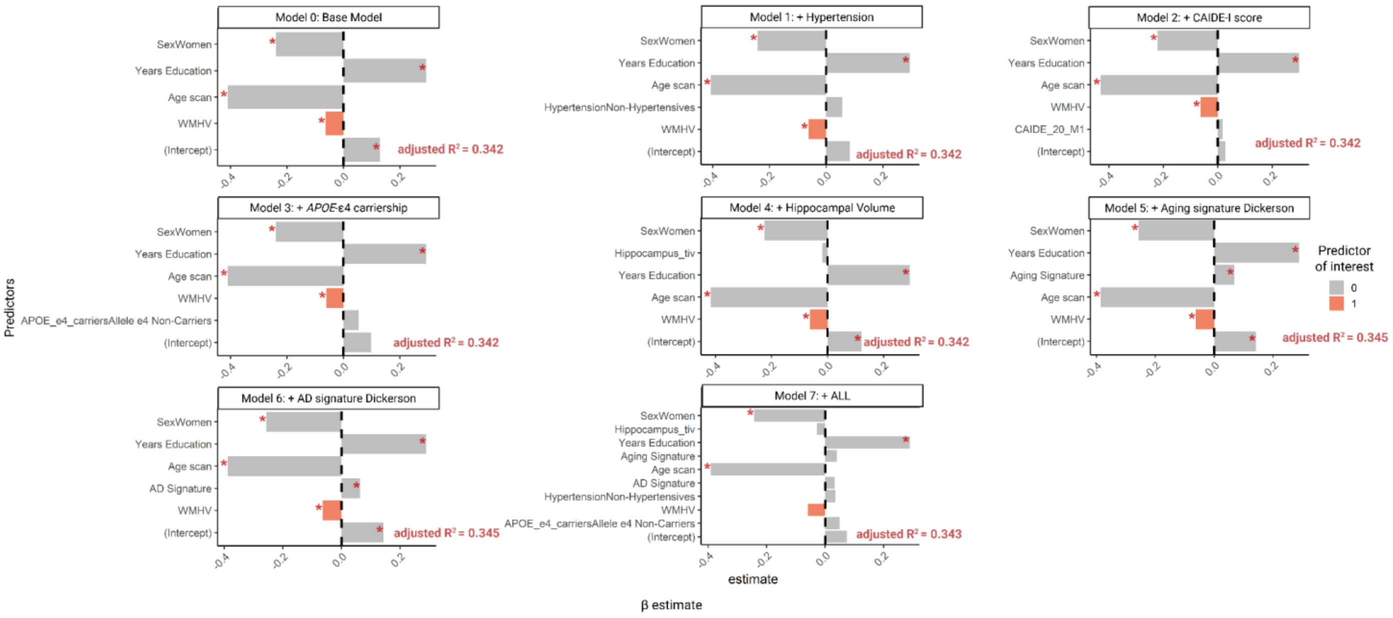

Figure 2: Results of the linear regression models exploring the association between global WMHV and executive functioning performance.
Footnote: in orange, the main predictor of interest. In gray, the rest of covariates that were added in the model. Subset of covariates [(Base Model: age, sex, years of education), (Model 1: age,sex, years of education, hypertension), (Model 2: age, sex, years of education, CAIDE-I score), (Model 3: age, sex, years of education, APOE-ε4 carriership), (Model 4: age, sex, years of education, normalized hippocampal volume), (Model 5: age, sex, years of education, aging-specific cortical thickness signature) , (Model 6: age, sex, years of education, AD-specific cortical thickness signature), (Model 7: all covariates)].

Similar results were found for the subset of non-hypertensive individuals at low risk to develop dementia late in life (n=909, 85% of the sample) [Figure 3].

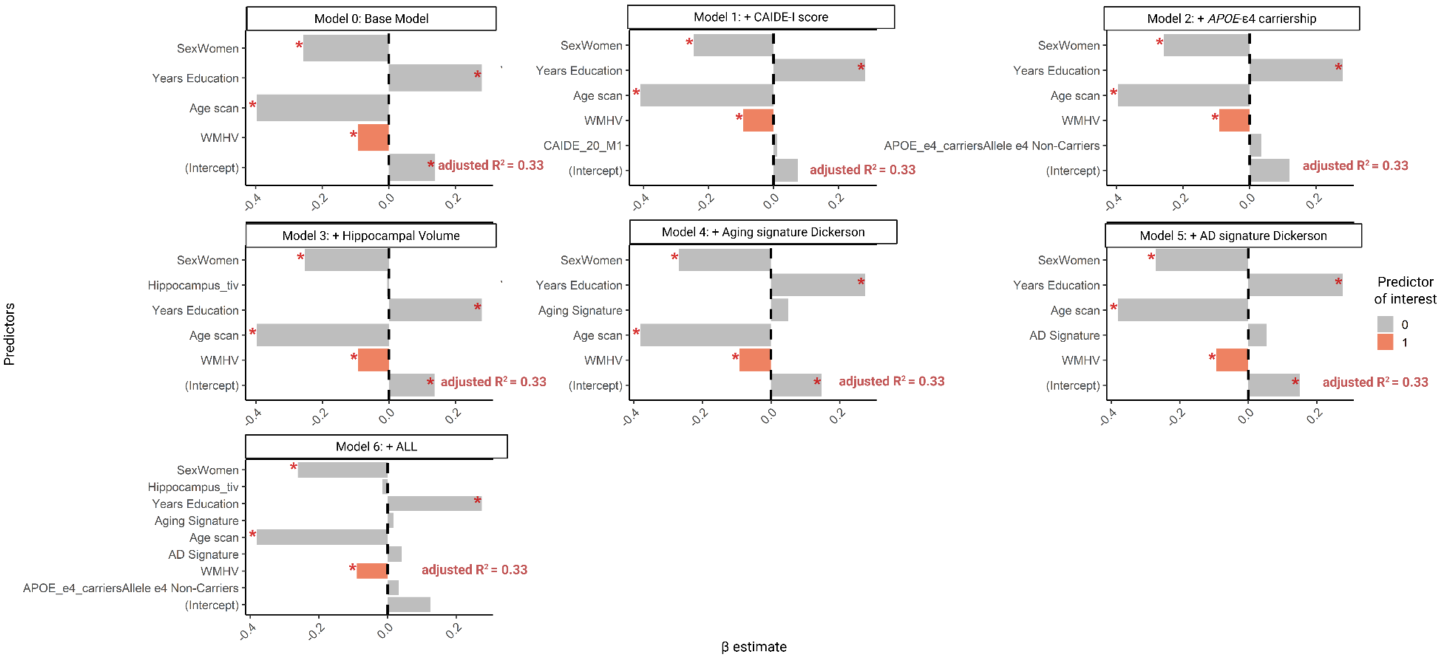

Figure 3: Results of the linear regression models exploring the association between global WMHV and executive functioning performance.
Footnote: in orange, the main predictor of interest. In gray, the rest of covariates that were added in the model. Subset of covariates [(Base Model: age, sex, years of education), (Model 1: age, sex, years of education, CAIDE-I score), (Model 2: age, sex, years of education, APOE-ε4 carriership), (Model 3: age, sex, years of education, normalized hippocampal volume), (Model 4: age, sex, years of education, aging-specific cortical thickness signature) , (Model 5: age, sex, years of education, AD-specific cortical thickness signature), (Model 6: all covariates)].

WMH volume exploration and potential relationship with the PRS-WMH

We visually explored the distribution of WMH volume (WMHV) in the ALFA sample. The histogram [Figure 4] shows the highly skewed behavior of WMHV.

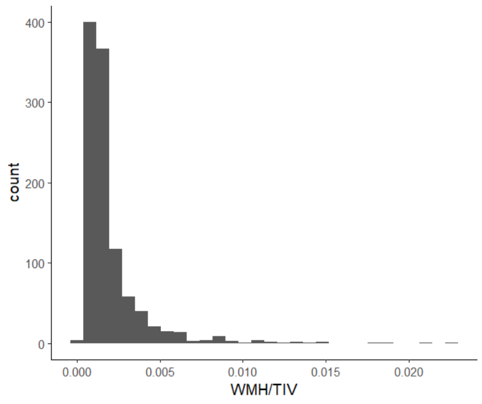

Figure 4. Histogram of WMHV distribution

Secondly, we used the *descdist* function from the *fitdistrplus* R package, to obtain descriptive parameters of the WMHV distribution assessing the skewness (measure of asymmetry, degree of deviation from a symmetric shape) and kurtosis (tails and peaks in the distribution) of the data [Table 1]. In Figure 5 skewness and kurtosis were plotted from bootstrap samples (n=100).

**Statistic**	**Value**
Min	0.0001389175
Max	0.02274563
Median	0.001400335
Mean	0.00202009
SD	0.002143368
Skewness	4.352858
Kurtosis	29.25986

Table 1: Summary statistics

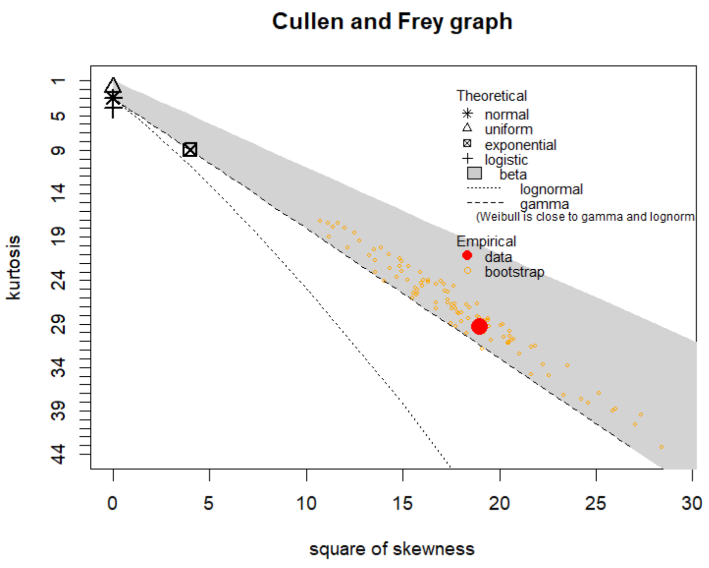

Figure 5: Skewness and kurtosis plot from bootstrap sample

The plot showed a likely gamma or beta distribution of WMHV [Figure 4]. We finally visually inspected the relationship between WMHV (rank-transformation for visualization purposes) and the PRS-WMH [Figure 6]. We can see a clear non-linear association between the PRS-WMH and WMHV in the whole sample, which would tend to a positive association until a certain point, from which it would then become negative. Nonetheless, if we explore the association in different subsets of the sample by splitting the PRS-WMH per quantile, we can see a positive association between both variables in all the subsets. Due to these observations, and the clear non-linear relationship between the two variables (a high scattering of the data points around the regression lines), we decided to explore this association using partial Spearman's rank correlation test, which is robust to distributional assumptions and explores the non-linear rank-based monotonic relationship between the variables.

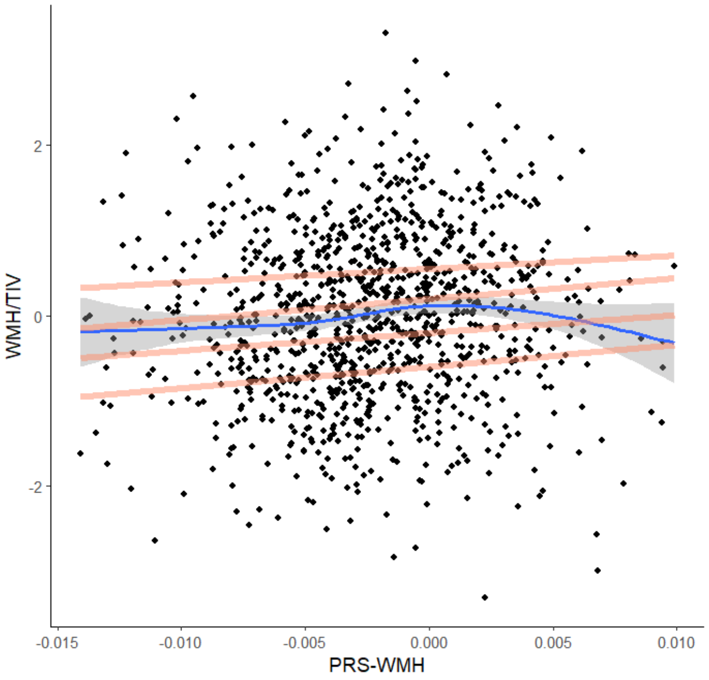

Figure 6: Relationship between WMHV and PRS-WMH. *Footnote: in blue, the non-linear relationship between PRS-WMH and WMHV, with the confidence interval indicated by the grey band. In red, the regression lines when splitting the data by quantiles of the PRS-WMH (0.25, 0.40, 0.55, 0.70).*

snpXplorer algorithm

For annotation and functional interpretation of the effect of the genetic variants included in the PRS_{WMH} in the ALFA sample, after clumping, we used the algorithm snpXplorer (Tesi et al., 2021). For the variant-gene mapping, the algorithm links the genetic variants to the most likely affected gene/s by (i) relating the variant to the gene when it is annotated to be coding by the Combined Annotation Dependent Depletion (CADD, v1.3), (ii) annotating a variant to genes based on found expression-quantitative-trait-loci (eQTL) from GTEx or (iii) mapping a variant to genes that are within a distance between 50kb and 500 kb [Figure 7]. The algorithm also reports whether the input SNPs and their likely related genes, have been previously associated with any trait in the GWAS-Catalog [Figure 8]. For this analysis, the software downloads all significant SNP-trait associations of all studies available in the GWAS-Catalog (v1.0.2, https://www.ebi.ac.uk/gwas/docs/file-downloads), which includes associations with $P < 9 \cdot 10^{-6}$.

A.

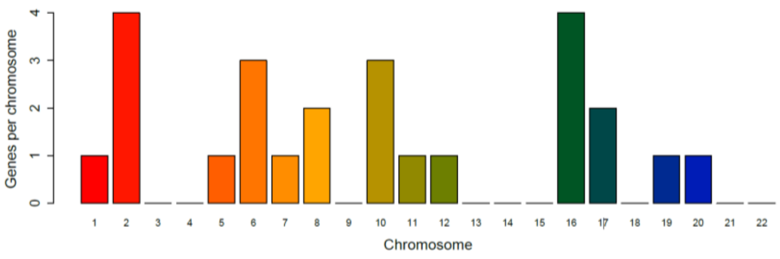

B.

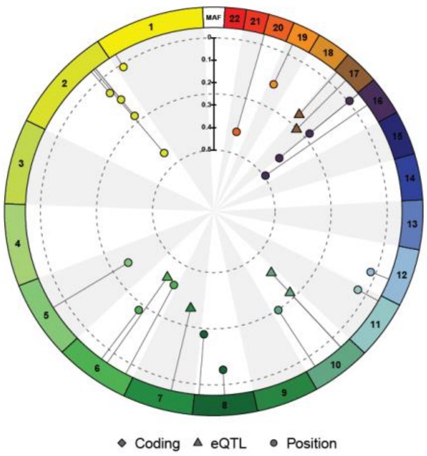

Figure 7. Distribution of the SNPs involved in the PRS-WMH across the genome. (A) Bar plot showing the number of genes identified per chromosome. (B) Circular Manhattan plot displaying the genomic positions of SNPs colored by chromosome, with distance from the center representing minor allele frequency (MAF). Different shapes indicate SNP annotations: coding variants (◇), eQTLs (▲), and positional SNPs (●).

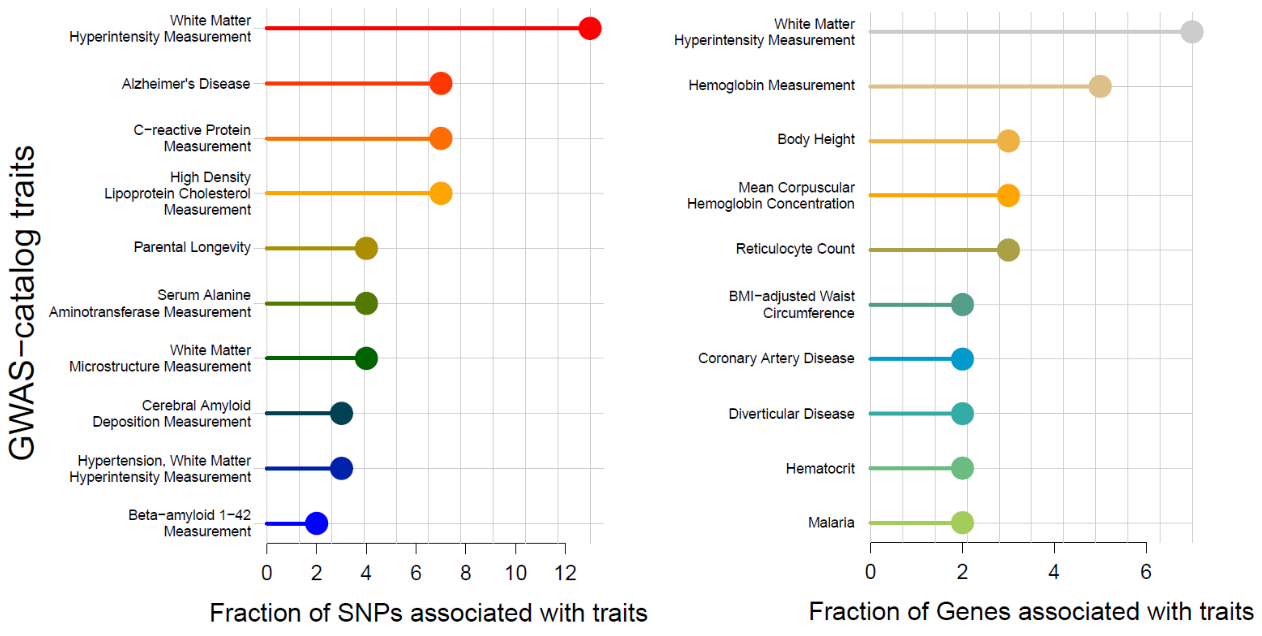

Figure 8. GWAS-Catalog traits that are associated with the input list of SNPs (left) and mapped genes (right).

Enrichment analysis approach

For the enrichment analysis, we used the one-sided version of Fisher's exact test to determine whether known biological functions were overrepresented in the gene list and calculate the probability of observing a set of genes in a particular biological pathway by chance. The annotation and consecutive enrichment analysis were performed including (i) all WMH-related SNPs from the GWAS of reference that remained after clumping with p-value$<5\cdot10^{-6}$ and (ii) all SNPs from the GWAS of reference, pre-clumping, with p-value$<5\cdot10^{-6}$ [Figure 9]. This approach gave a better interpretability of the biological pathways leading to the presence of WMH. On the one hand, by retaining the full spectrum of SNPs associated with WMH volumes, we provided a more comprehensive interpretation of the biological pathways contributing to WMH without losing relevant biological information influenced by clumping-

related limitations (e.g. allele frequencies, filtering parameters) [Figure 9]. On the other hand, by selecting SNPs after the clumping (r^2=0.1, kbp=250), we removed data redundancy and increased the statistical power of the analyses by decreasing the number of hypotheses to contrast.

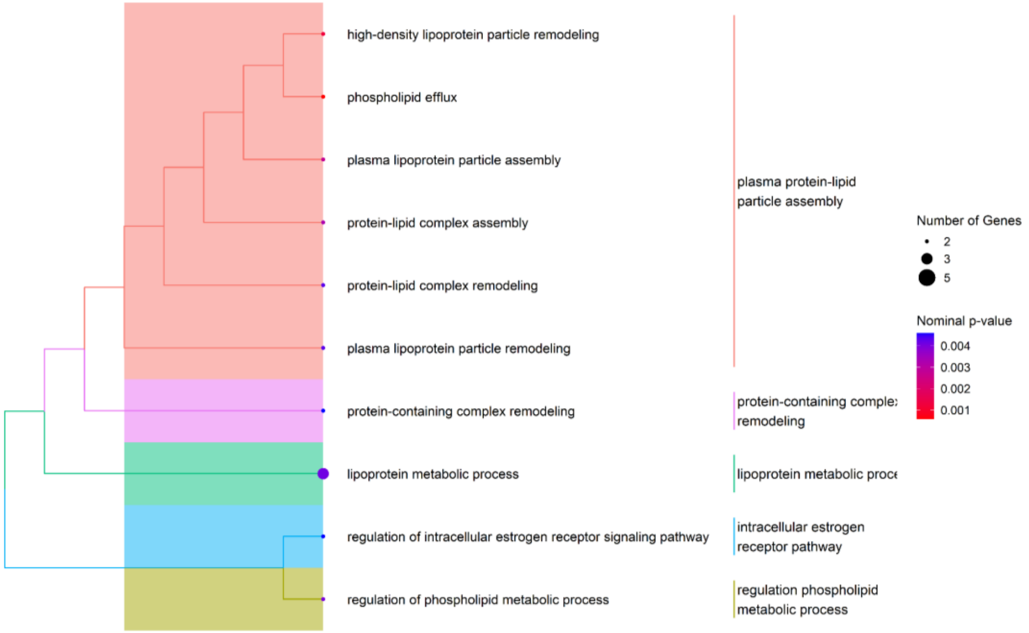

Figure 9. Results of the enrichment analysis showing the main biological processes in which genes annotated to the list of SNPs, before clumping, are involved. *Legend: One-sided version of Fisher's exact test was performed to determine whether known biological functions were overrepresented or enriched in the gene list and calculate the probability of observing a set of genes in a particular biological pathway by chance. Significant results were reported at nominal p-value <0.05 and biological mechanisms were grouped into main functions based on their similarity.*

These two scenarios gave a wider overview of the biological mechanisms underlying the presence of WMH.

Based on the enrichment analysis results we hypothesized that either lipid-related mechanisms or vascular-related factors could act as potential mediators in the link between the genetic risk of WMH (biological substrate) and WMHV, the actual structural phenotype.

Dyslipidemia as a construct based on serum lipid levels, self-reported use of medication and self-reported hypercholesterolemia

A subset of 237 individuals had available Tchol, HDL, LDL and TG measurements. Positive status for dyslipidemia was defined based on either the presence of pathological lipid levels (according to standard clinical cut-offs), self-reported use of lipid-modifying therapies or self-reported hypercholesterolemia. Conversely, participants were classified as non-dyslipidemic ("normal" lipid status) if the previous conditions were not met [Figure 10]. The group with the highest proportion of individuals in the dyslipidemic category due to pathological lipids levels, was found to be for LDL.

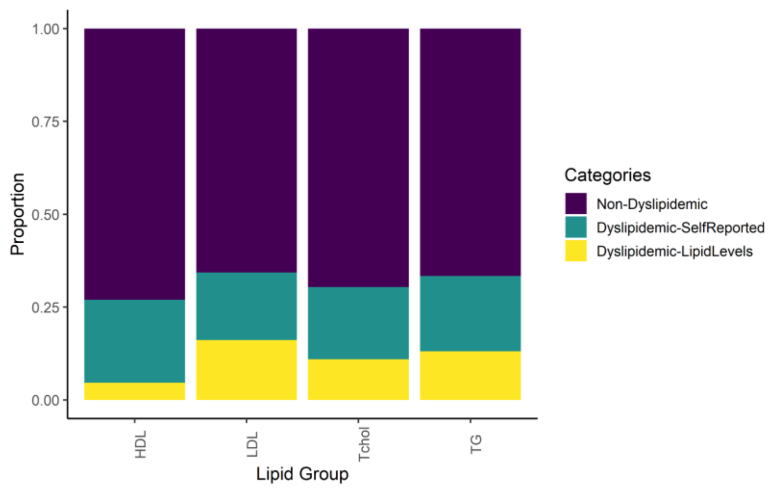

Figure10. Barplots with the characterization of the profile of participants classified as dyslipidemic (either via self-reported information or based on pathological lipid levels) and non-dyslipidemic in the sample.

Within the dyslipidemic group, we could split by "pathological" levels (levels outside the clinically normal ranges) and "non-pathological" levels (clinically normal levels). Individuals in the dyslipidemic non-pathological group were always participants who either self-reported the use of lipid-modifying drugs, hypercholesterolemia or both [Figure 11]. For all group comparisons, there were significant differences in the median value for the levels of lipids in serum between the two dyslipidemic groups [Figure 11].

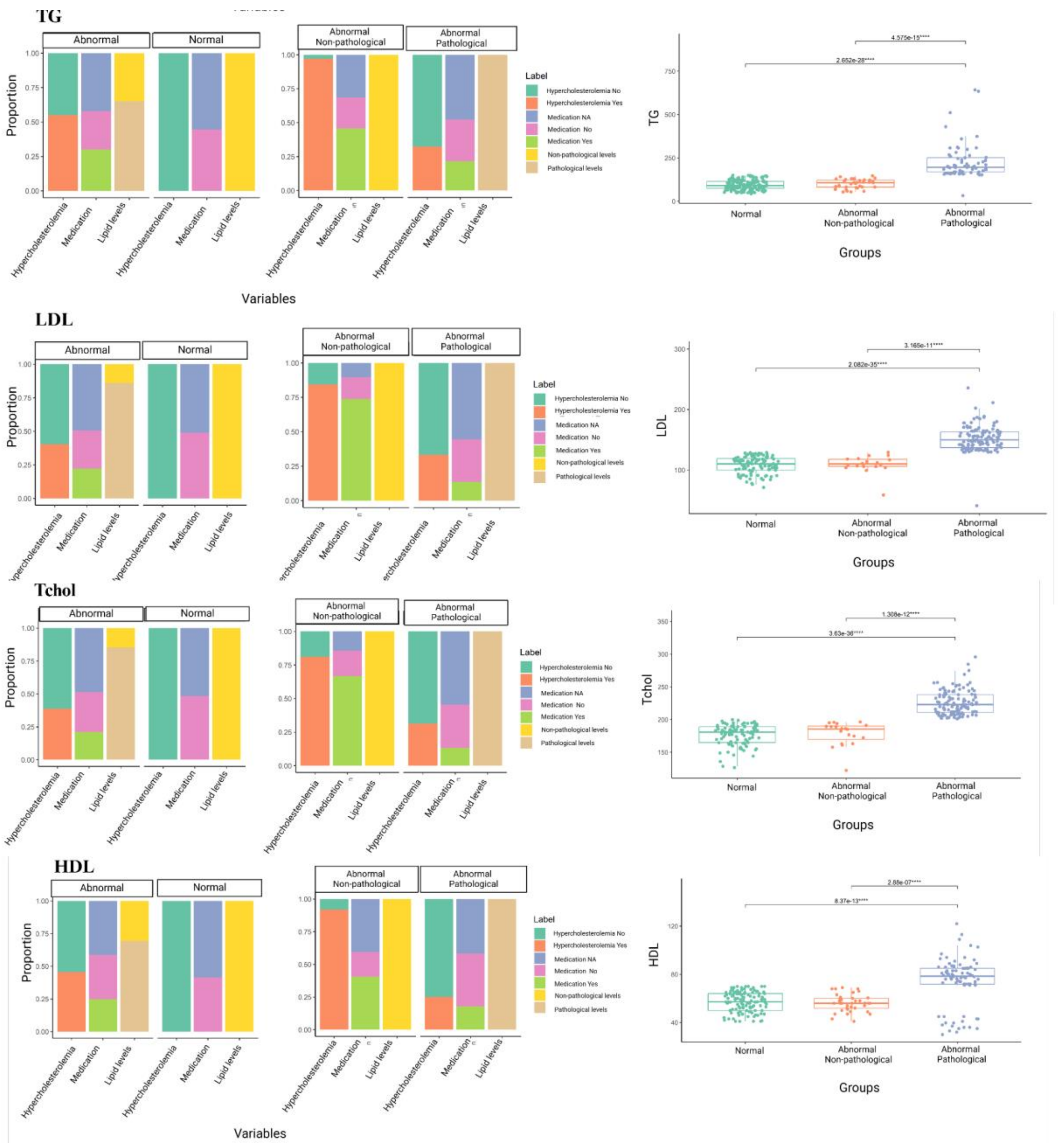

Figure 11. On the left, characterization of the profile of participants classified as dyslipidemic and non-dyslipidemic in the sample based on the levels of lipids in serum and the self-reported status for hypercholesterolemia and use of lipid-modifying therapies. On the right, raw levels of lipid levels in serum across groups. *Legend: Pairwise Wilcoxon tests were used to compare the median values of serum lipid levels between groups. Significant p-values were reported after FDR correction per lipid family.*

With this characterization in mind, we performed linear regression models to explore the relationship between dyslipidemia and WMHV. Association analyses showed that displaying dyslipidemia based on either self-reported hypercholesterolemia, use of medication or Tchol ≥ 240 mg/dl, was negatively associated associated with larger WMHV (β=-0.274 [-0.539, -0.009], p-value=0.042) [Table 2]. While no other associations reached statistical significance,

the direction of the effects indicated negative relationships between dyslipidemia status and larger WMHV. These results were contrary to the expected hypothesis we had.

Model	N	Beta	SE	P-value	Low CI	High CI	adj R2
LDL							
WMH volume~ dyslipidemia + age_scan + Sex	236	- 0.174	0.131	0.184	-0.434	0.084	0.086
WMH volume~ dyslipidemia + age_scan + Sex + APOE_e4_carriership	236	- 0.196	0.131	0.134	-0.453	0.061	0.101
TG							
WMH volume~ dyslipidemia + age_scan + Sex	237	- 0.129	0.133	0.337	-0.391	0.134	0.082
WMH volume~ dyslipidemia + age_scan + Sex + APOE_e4_carriership	237	- 0.132	0.132	0.321	-0.393	0.129	0.096
HDL							
WMH volume~ dyslipidemia + age_scan + Sex	237	- 0.166	0.141	0.240	-0.443	0.112	0.084
WMH volume~ dyslipidemia + age_scan + Sex + APOE_e4_carriership	237	- 0.163	0.139	0.245	-0.434	-0.113	0.097
TCHOL							
WMH volume~ dyslipidemia + age_scan + Sex	237	- 0.251	0.132	**0,065**	-0.517	0.015	0.092
WMH volume~ dyslipidemia + age_scan + Sex + APOE_e4_carriership	237	- 0.274	0.134	**0.042**	-0.539	-0.009	0.108

Table 2. Linear regression models assessing the relationship between dyslipidemia and WMH volume. *Footnote: WMH volume was transformed using Box-Cox transformation based on an iterative process seeking for the best fit (bestNormalize R package). Normality of the residuals and overall performance of the models was checked with the performance R package. Sensitivity analyses were performed adding APOE - e4 carriership as confounder. The reference group for dyslipidemia status refers to the non-dyslipidemic group.*

The main difference between dyslipidemic and non-dyslipidemic individuals in the sample was observed in the self-reported use of medication [Figure 12]. No other sources of potential biases (e.g. age, sex, APOE-e4 carriership) were observed.

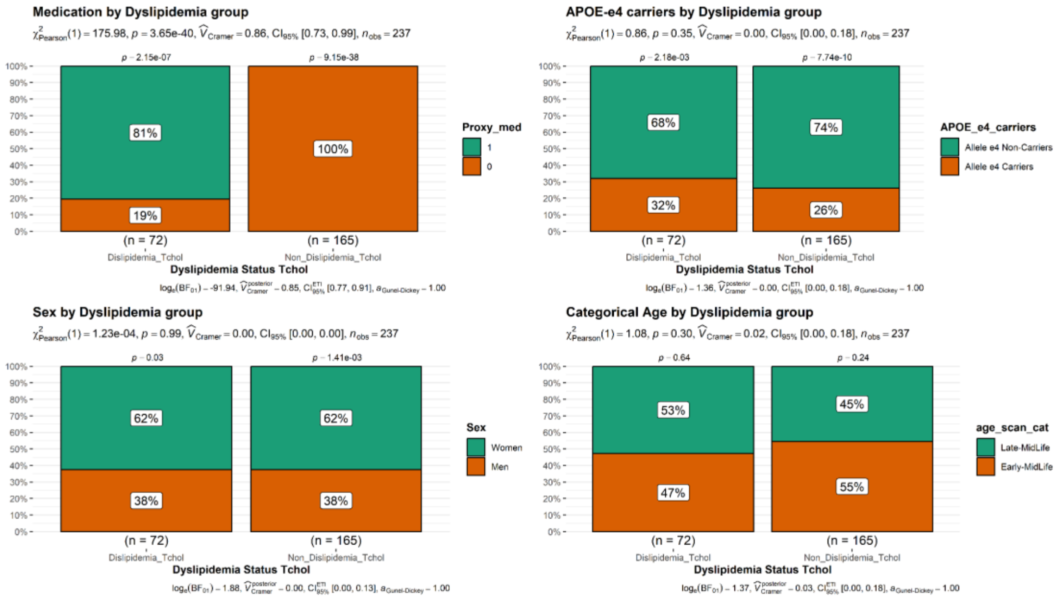

Figure 12. Bar charts displaying statistical chi square test results for categorical data. *Footnote: age was defined as a categorical variable based on the median (Early-MidLife = Age < quantile 0.5 age).*

We additionally explored the distribution of WMHV across dyslipidemic, splitting based on the self-reported information or pathological lipid levels, and non-dyslipidemic [Figure 13]. No significant differences were found, but the most extreme WMHV were observed in non-dyslipidemic individuals.

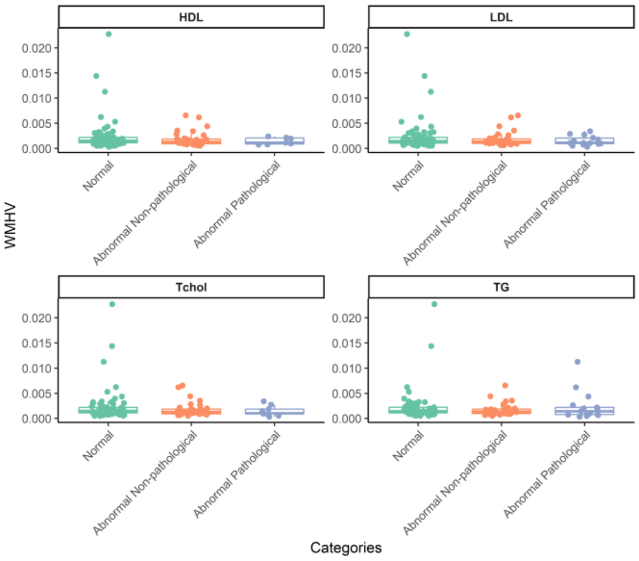

Figure 13. Boxplots displaying WMHV in non-dyslipidemic, dyslipidemic individuals based on self-reported information and dyslipidemic based on lipid levels in serum.

To better capture the effect of medication, we split by use of medication within each category of the binary dyslipidemic construct (dyslipidemic vs non-dyslipidemic) [Figure 14]. Although there were not significant differences, the highest WMHV were observed in non-dyslipidemic.

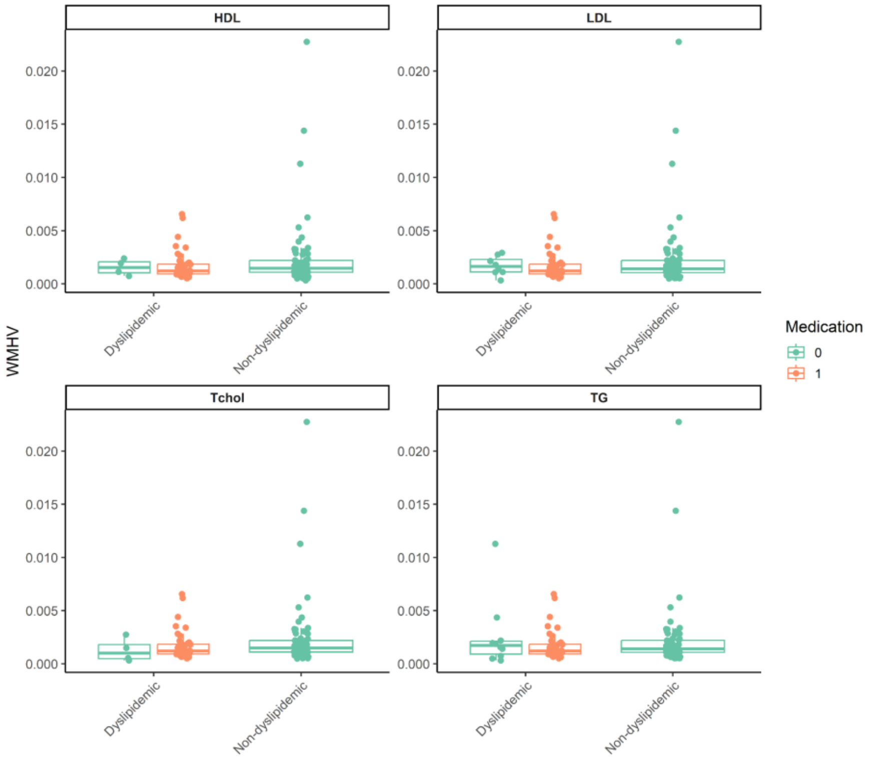

Figure 14. Boxplots displaying WMHV in non-dyslipidemic, dyslipidemic individuals based on self-reported information and dyslipidemic based on lipid levels in serum. WMHV is reported based on the use of medication within dyslipidemic groups

Overall we obtained two main insights regarding the behavior of the sample based on lipid levels in serum and WMHV when we compared across the dyslipidemic groups. The group of dyslipidemic individuals who self-reported use of medication or hypercholesterolemia displayed similar lipid levels compared to the non-dyslipidemic group of individuals, with clinically normal levels of lipids in serum. Medication could be one of the main reasons affecting these observations, as observed in Figure 11. Altogether, these observations may indicate that the use of the dyslipidemia composite would be closer to the use of the binary variable referring to the use of medication than a real biological profile based on lipid levels. The use of medication may have affected the already registered lipid levels, as observed when we split the dyslipidemic group in two subgroups, and in consequence, mask any potential association with WMHV and bias the definition of dyslipidemic profile. Therefore, we may not be able to explore the potential of lipid levels in serum as potential biomarkers of WMH.

Distribution of Blood Pressure Measurements in the sample of study

We assessed the distribution of BP measurements in the sample, displayed by genetic risk of WMH (High risk WMH: PRS_{WMH} > quantile 0.8 PRS_{WMH}) [Figure 15], and compared the median values between hypertensive and non-hypertensive individuals for all the measurements [Figure 16]. The binary variable for hypertension was defined based on the measured systolic blood pressure (SBP) (hypertensive > 140 mmHg) and self-reported hypertension or use of antihypertensive treatment.

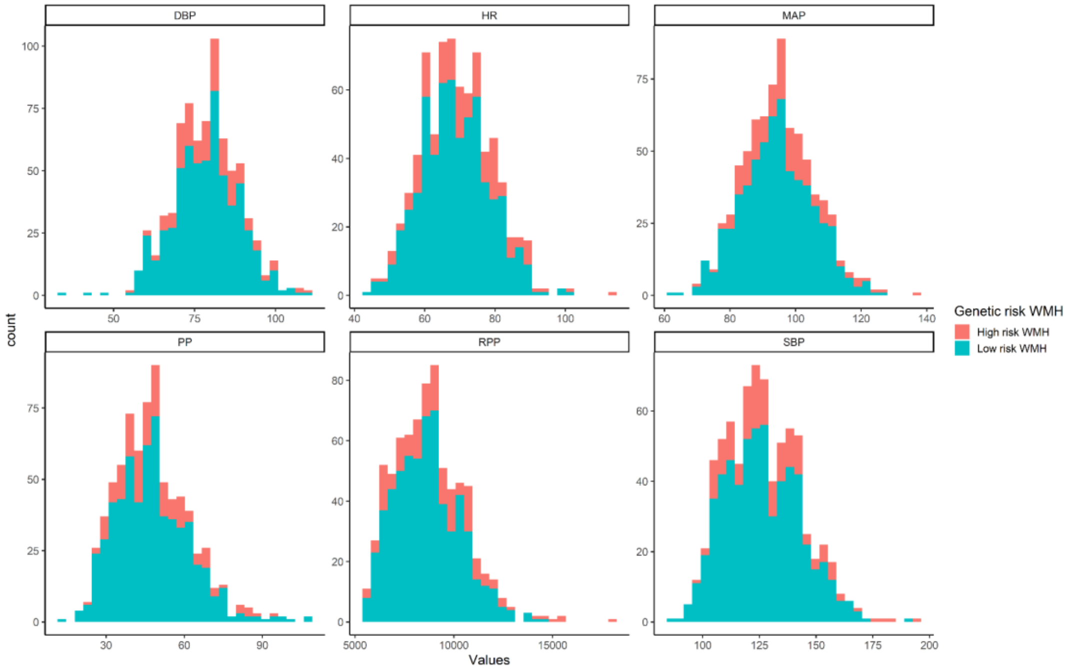

Figure 15. Histograms displaying the distribution of DBP, HR, MAP, PP, RPP and SBP in the sample. *Footnote: histograms were coloured based on the participants' genetic risk for WMH. Legend: DBP (diastolic blood pressure); HR (heart rate); MAP (mean arterial pressure); PP (pulse pressure); RPP (rate-pressure product); SBP (systolic blood pressure).*

Hypertensive individuals displayed higher DBP, MAP, PP, RPP and SBP when compared to non-hypertensive individuals in the sample [Figure 16]. Hypertensive individuals were not split into groups based on use of medication as, among individuals reporting use of medication in this subset, only 1 out of 585 was using anti hypertensive treatments.

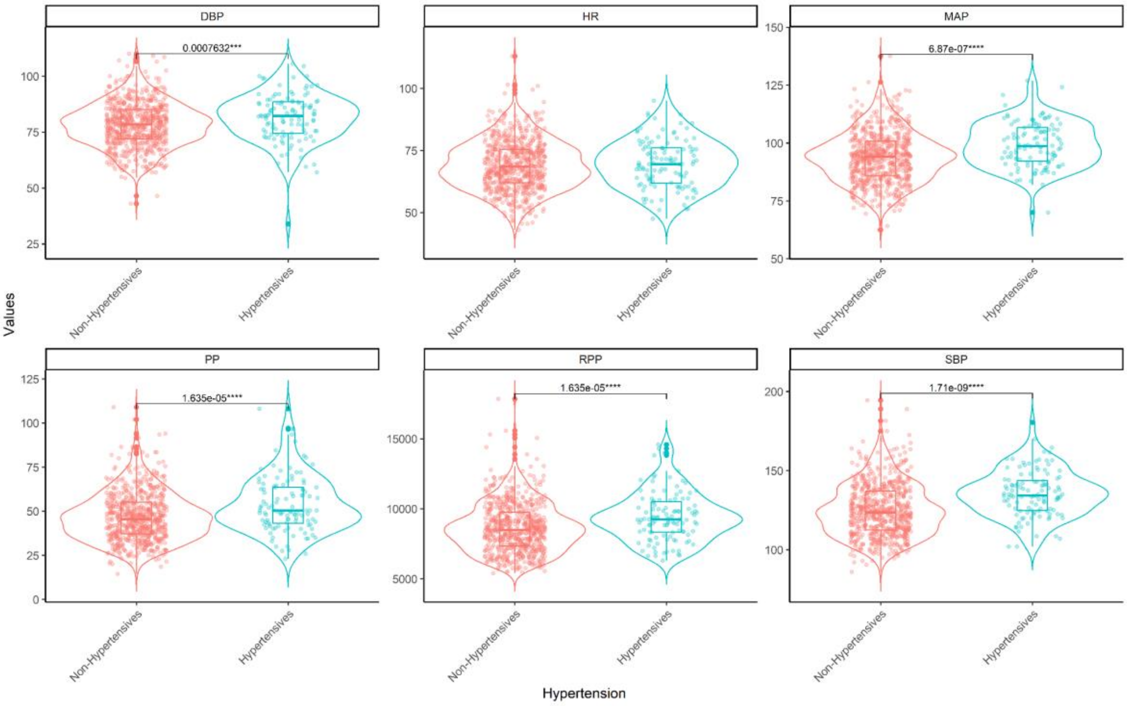

Figure 15. Violin plots showing the distribution of DBP, HR, MAP, PP, RPP and SBP in hypetensive and non-hypertensive individuals. *Footnote: the median value of all blood pressure measurements was compared between groups using a wilcoxon test (***FDR p-value <.001)*
