# Supplementary material for: Polygenic risk for white matter hyperintensities is associated with early cerebrovascular events partly through hemodynamic measures in cognitively unimpaired middle-aged and older adults with low cardiovascular risk
Source: Front Neurol. 2026 Jan 5;16:1667424. doi: 10.3389/fneur.2025.1667424 (PMC12812530; doi:10.3389/fneur.2025.1667424)
Supplement: Supplementary file 4 [file Data_Sheet_4.pdf]

Supplementary Tables

Risk factor	CAIDE-I risk score
**Age, years**	
<47	0
47-53	3
>53	4
**Education, years**	
≥10	0
7-9	2
0-6	3
**Sex**	
Women	0
Men	1
**Systolic blood pressure**	
≤140 mmHg	0
>140 mmHg	2
**BMI, kg/m2**	
≤30	0
>30	2
**Hypercholesterolemia**	
No	0
Yes	2
**Physical activity**	
Active	0
Inactive	1
**TOTAL**	**15**

Supplementary Table 1. Cardiovascular risk factors contributing to dementia included in the CAIDE-I score and their corresponding punctuation. *Legend: higher punctuation involves higher risk for dementia. Cutoffs and punctuations were derived from Kivipelto et al., 2006.*

GWAS of reference	
Disease/Condition Classification	Brain Endophenotypes
Disease/Condition Description	*White Matter Hyperintensities*
Acronym	*WMH*
N	18,381
N Cases	Continuous outcome
N Controls	Continuous outcome
Genetic Ancestry	Caucasian
Genome Assembly	GRCh37/hg19
Base GWAS-First Author	*Persyn et al., 2020*
Link Manuscript	https://pubmed.ncbi.nlm.nih.gov/32358547/
Access GWAS-Summary (LINK)	http://www.kp4cd.org/dataset_downloads/stroke
**Summary statistic of the GWAS**	
Nº of SNPs in the GWAS	9,712,6789
Nº of SNPs included in the PRS with p-value < $5{\cdot}10^{-6}$	708
Nº of SNPs included in the PRS after the clumping with p-value < $5{\cdot}10^{-6}$	25

Supplementary Table 2: Detailed information of the GWAS of reference and the summary statistics for the PRS-WMH computation. *Legend: GWAS (Genome-wide association study); SNP (single nucleotide polymorphism); N (sample size); PRS (Polygenic risk score). The optimal threshold for the p-value was determined to be $5{\cdot}10^{-6}$ as it included a sufficient number of SNPs to capture genetic variability in the sample and accounted for the highest variance in WMH volumes compared to the rest of scores.*

CHR	BP	SNP	A1	A2	BETA	SE	P	Gene
17	73888354	rs34974290	A	G	0,110	0,012	0,000	TRIM65
2	56128091	rs7596872	A	C	0,122	0,016	0,000	EFEMP1
17	43127708	rs12950988	A	G	-0,071	0,010	0,000	DCAKD
6	151018909	rs6940540	G	T	0,059	0,010	0,000	PLEKHG1
2	43103440	rs12615761	G	T	-0,073	0,013	0,000	
7	100361675	rs2293767	A	G	-0,059	0,011	0,000	ZAN
16	87237568	rs12928520	T	C	0,055	0,010	0,000	
10	105614452	rs11191829	T	G	-0,054	0,010	0,000	SH3PXD2A
16	51442679	rs1948948	T	C	-0,052	0,010	0,000	LOC102723323
16	87250519	rs28655023	G	A	-0,050	0,010	0,000	
10	105459116	rs4630220	A	G	-0,053	0,011	0,000	SH3PXD2A
2	43098013	rs7594680	C	T	-0,058	0,012	0,000	
19	45411941	rs429358	C	T	0,066	0,013	0,000	APOE
20	56559939	rs6070327	C	T	-0,049	0,010	0,000	

6	97050265	rs1855533	C	T	-0,050	0,010	0,000	FHL5
5	82857870	rs7733216	C	T	0,059	0,012	0,000	VCAN
8	11154358	rs7836248	A	G	-0,064	0,013	0,000	MTMR9
1	230821062	rs16852286	C	T	-0,091	0,019	0,000	COG2
12	46595684	rs7977873	T	C	0,078	0,017	0,000	SLC38A1
10	25192123	rs10764509	A	G	0,046	0,010	0,000	PRTFDC1
6	80191532	rs9294147	G	A	-0,055	0,012	0,000	LOC100506851
2	48759845	rs10184783	T	C	0,045	0,010	0,000	STON1
16	87593783	rs117428453	A	G	-0,161	0,035	0,000	
8	74452451	rs34207938	C	A	-0,109	0,024	0,000	STAU2
11	120212601	rs61528732	C	T	-0,100	0,022	0,000	ARHGEF12

Supplementary Table 3. Summary statistics of the SNPs included in the PRS-WMH calculation under the threshold $5 \cdot 10^{-6}$.

Term	Beta	OR	SE	P-value	Low CI	High CI
(Intercept)	-2.618	0.073	0.206	**<0.001**	-3.04	-2.232
PRS-WMH	0.086	1.090	0.119	0.471	-0.147	0.322
Age	0.707	2.028	0.127	**<0.001**	0.463	0.961
Sex:Women	-0.356	0.700	0.238	0.135	-0.821	0.115
Hypertension:Hypertensive	0.622	1.863	0.267	**0.02**	0.085	1.136

Supplementary Table 4. Results for the logistic regression model assessing the association between the PRS of WMH and WMH pathological levels.

locus	ID	snp consequence	snp consequence gene	phred	coding snp	pos hg38	code gtex	eqtl	eqtl tissue	sqtl	sqtl tissue	positional mapping	source	gene
10:105459116	rs4630220	regulatory	NA	5.852	NA	103699358	10_103699358_	CNNM2	Whole_Blood	NA	NA	Not_done	sqtl+eqtl+cadd	CNNM2
10:105614452	rs11191829	regulatory	NA	15.010	NA	103854694	10_103854694_	SLK.SH3PXD2A,STN1	Whole_Blood,Whole_Blood,Whole_Blood	NA	NA	Not_done	sqtl+eqtl+cadd	SLK,SH3PXD2A, STN1
17:43127708	rs12950988	regulatory	NA	3.757	NA	45050340	17_45050340_	DCAKD	Whole_Blood	NA	NA	Not_done	sqtl+eqtl+cadd	DCAKD
17:73888354	rs34974290	regulatory	NA	22.700	NA	75892273	17_75892273_	UNC13D, GALK1, MRPL38,	Whole_Blood,Whole_Blood,Whole_Blood,Whole_Blood	WBP2, MRPL38	Whole_Blood, Whole_Blood	Not_done	sqtl+eqtl+cadd	UNC13D,GALK1,MRPL38, WBP2
6:97050265	rs1855533	regulatory	NA	2.314	NA	96602389	6_96602389_	UFL1	Whole_Blood	NA	NA	Not_done	sqtl+eqtl+cadd	UFL1
7:100361675	rs2293767	regulatory	NA	13.180	NA	100764052	7_100764052_	MOSPD3,GIGYF1,EPHB4	Whole_Blood,Whole_Blood,Whole_Blood	NA	NA	Not_done	sqtl+eqtl+cadd	MOSPD3, GIGYF1,EPHB4
1:230821062	rs16852286	intron	COG2	0.514	NA	230685316	1_230685316_	NA	NA	NA	NA	COG2,AGT	positional	COG2
10:25192123	rs10764509	intron	PRTFDC1	1.505	NA	24903194	10_24903194_	NA	NA	NA	NA	PRTFDC1	positional	PRTFDC1

11:120212601	rs61528732	intron	ARHGEF12	4.987	NA	120341892	11_120341892_	NA	NA	NA	NA	POU2F3,TMEM136,ARHGEF12	positional	ARHGEF12
12:46595684	rs7977873	intron	SLC38A1	0.020	NA	46201901	12_46201901_	NA	NA	NA	NA	SLC38A1	positional	SLC38A1
16:51442679	rs1948948	regulatory	NA	18.190	NA	51408768	16_51408768_	NA	NA	NA	NA	SALL1	positional	SALL1
16:87237568	rs12928520	regulatory	NA	1.362	NA	87203962	16_87203962_	NA	NA	NA	NA	C16orf95	positional	C16orf95
16:87250519	rs28655023	intron,non_coding	NA	8.691	NA	87216913	16_87216913_	NA	NA	NA	NA	C16orf95,FBXO31	positional	C16orf95,FBXO31
16:87593783	rs117428453	regulatory	NA	0.352	NA	87560177	16_87560177_	NA	NA	NA	NA	ZCCHC14,JPH3	positional	ZCCHC14,JPH3
19:45411941	rs429358	downstream	TOMM40	17.400	NA	44908684	19_44908684_	NA	NA	NA	NA	PVRL2,TOMM40,APOE,APOC1	positional	TOMM40
2:43098013	rs7594680	intergenic	NA	6.262	NA	42870873	2_42870873_	NA	NA	NA	NA	MTA3,OXER1,HAAO	positional	MTA3,OXER1,HAAO
2:43103440	rs12615761	regulatory	NA	2.490	NA	42876300	2_42876300_	NA	NA	NA	NA	MTA3,OXER1,HAAO	positional	MTA3,OXER1,HAAO
2:48759845	rs10184783	intron	STON1-GTF2A1L	2.145	NA	48532706	2_48532706_	NA	NA	NA	NA	PPP1R21,STON1-GTF2A1L,STON1	positional	STON1-GTF2A1L

2:56128091	rs7596872	regulatory	NA	8.934	NA	55900956	2_55900956_	NA	NA	NA	NA	EFEMP1	positional	EFEMP1
20:56559939	rs6070327	intergenic	NA	0.042	NA	57984883	20_57984883_	NA	NA	NA	NA	C20orf85	positional	C20orf85
5:82857870	rs7733216	regulatory	NA	5.547	NA	83562051	5_83562051_	NA	NA	NA	NA	VCAN	positional	VCAN
6:151018909	rs6940540	regulatory	NA	16.530	NA	150697773	6_150697773_	NA	NA	NA	NA	PLEKHG1	positional	PLEKHG1
6:80191532	rs9294147	downstream	LCA5	1.319	NA	79481815	6_79481815_	NA	NA	NA	NA	LCA5	positional	LCA5
8:11154358	rs7836248	intron	MTMR9	0.321	NA	11296849	8_11296849_	NA	NA	NA	NA	MTMR9	positional	MTMR9
8:74452451	rs34207938	intron	STAU2	2.580	NA	73540216	8_73540216_	NA	NA	NA	NA	STAU2	positional	STAU2

Supplementary Table 5. Annotation of the genetic variants included in the PRS-WMH to their nearest genes. *Legend: we used the algorithm snpXplorer (Tesi et al., 2021) for annotation and functional interpretation of the effects of genetic variants. For the variant-gene mapping, the algorithm links the genetic variants to the most likely affected gene/s by (i) relating the variant to the gene when it is annotated to be coding by the Combined Annotation Dependent Depletion (CADD, v1.3), (ii) annotating a variant to genes based on found expression-quantitative-trait-loci (eQTL) from GTEx or (iii) mapping a variant to genes that are within a distance between 50kb and 500 kb.*

ID	Description	GeneRatio	BgRatio	p-value	FDR	q-value	geneID	Count
GO:0033700	phospholipid efflux	2/38	11/11590	0,001	0,374	0,353	APOE/APOC1	2
GO:0034375	high-density lipoprotein particle remodeling	2/38	16/11590	0,001	0,374	0,353	APOE/APOC1	2
GO:0034377	plasma lipoprotein particle assembly	2/38	22/11590	0,002	0,374	0,353	APOE/APOC1	2
GO:0045940	positive regulation of steroid metabolic process	2/38	24/11590	0,003	0,374	0,353	APOE/APOC1	2
GO:0065005	protein-lipid complex assembly	2/38	24/11590	0,003	0,374	0,353	APOE/APOC1	2
GO:0034368	protein-lipid complex remodeling	2/38	27/11590	0,003	0,374	0,353	APOE/APOC1	2
GO:0034369	plasma lipoprotein particle remodeling	2/38	27/11590	0,003	0,374	0,353	APOE/APOC1	2
GO:0034367	protein-containing complex remodeling	2/38	28/11590	0,004	0,374	0,353	APOE/APOC1	2
GO:0042157	lipoprotein metabolic process	3/38	99/11590	0,004	0,374	0,353	APOE/APOC1/NMT1	3
GO:0060999	positive regulation of dendritic spine development	2/38	30/11590	0,004	0,374	0,353	STAU2/APOE	2
GO:0033146	regulation of intracellular estrogen receptor signaling pathway	2/38	31/11590	0,005	0,374	0,353	UFL1/WBP2	2
GO:1903725	regulation of phospholipid metabolic process	2/38	31/11590	0,005	0,374	0,353	MTMR9/APOC1	2
GO:0034381	plasma lipoprotein particle clearance	2/38	34/11590	0,005	0,414	0,391	APOE/APOC1	2
GO:0071827	plasma lipoprotein particle organization	2/38	39/11590	0,007	0,439	0,414	APOE/APOC1	2
GO:0071825	protein-lipid complex subunit organization	2/38	41/11590	0,008	0,439	0,414	APOE/APOC1	2
GO:0033344	cholesterol efflux	2/38	44/11590	0,009	0,439	0,414	APOE/APOC1	2
GO:0060998	regulation of dendritic spine development	2/38	44/11590	0,009	0,439	0,414	STAU2/APOE	2
GO:0030520	intracellular estrogen receptor signaling pathway	2/38	45/11590	0,009	0,439	0,414	UFL1/WBP2	2
GO:0032371	regulation of sterol transport	2/38	45/11590	0,009	0,439	0,414	APOE/APOC1	2
GO:0032374	regulation of cholesterol transport	2/38	45/11590	0,009	0,439	0,414	APOE/APOC1	2
GO:0051055	negative regulation of lipid biosynthetic process	2/38	46/11590	0,010	0,439	0,414	APOE/APOC1	2
GO:0048167	regulation of synaptic plasticity	3/38	158/11590	0,015	0,439	0,414	STAU2/SLC38A1/APOE	3

GO:0030100	regulation of endocytosis	3/38	164/11590	0,016	0,439	0,414	APOE/APOC1/STON1	3
GO:0097006	regulation of plasma lipoprotein particle levels	2/38	60/11590	0,016	0,439	0,414	APOE/APOC1	2
GO:0032787	monocarboxylic acid metabolic process	5/38	472/11590	0,018	0,439	0,414	GALK1/SLC38A1/APOC1/ACOX1/ACBD4	5
GO:0030258	lipid modification	3/38	173/11590	0,019	0,439	0,414	MTMR9/APOC1/ACOX1	3
GO:0097061	dendritic spine organization	2/38	66/11590	0,020	0,439	0,414	STAU2/APOE	2
GO:0033143	regulation of intracellular steroid hormone receptor signaling pathway	2/38	67/11590	0,020	0,439	0,414	UFL1/WBP2	2
GO:0099175	regulation of postsynapse organization	2/38	68/11590	0,021	0,439	0,414	STAU2/APOE	2
GO:0042158	lipoprotein biosynthetic process	2/38	70/11590	0,022	0,439	0,414	APOE/NMT1	2
GO:0006641	triglyceride metabolic process	2/38	72/11590	0,023	0,439	0,414	APOE/APOC1	2
GO:0015914	phospholipid transport	2/38	73/11590	0,024	0,439	0,414	APOE/APOC1	2
GO:0060996	dendritic spine development	2/38	76/11590	0,026	0,439	0,414	STAU2/APOE	2
GO:0106027	neuron projection organization	2/38	76/11590	0,026	0,439	0,414	STAU2/APOE	2
GO:0001649	osteoblast differentiation	3/38	195/11590	0,026	0,439	0,414	UFL1/VCAN/H3-3A	3
GO:0010232	vascular transport	2/38	77/11590	0,026	0,439	0,414	SLC38A1/APOE	2
GO:0150104	transport across blood-brain barrier	2/38	77/11590	0,026	0,439	0,414	SLC38A1/APOE	2
GO:0030301	cholesterol transport	2/38	78/11590	0,027	0,439	0,414	APOE/APOC1	2
GO:0045833	negative regulation of lipid metabolic process	2/38	79/11590	0,028	0,439	0,414	APOE/APOC1	2
GO:0019218	regulation of steroid metabolic process	2/38	81/11590	0,029	0,439	0,414	APOE/APOC1	2
GO:2001251	negative regulation of chromosome organization	2/38	81/11590	0,029	0,439	0,414	STN1/H3-3A	2
GO:0062014	negative regulation of small molecule metabolic process	2/38	84/11590	0,031	0,439	0,414	APOE/APOC1	2
GO:0006707	cholesterol catabolic process	1/38	10/11590	0,032	0,439	0,414	APOE	1
GO:0008298	intracellular mRNA localization	1/38	10/11590	0,032	0,439	0,414	STAU2	1

GO:0015937	coenzyme A biosynthetic process	1/38	10/11590	0,032	0,439	0,414	DCAKD	1
GO:0016127	sterol catabolic process	1/38	10/11590	0,032	0,439	0,414	APOE	1
GO:0033148	positive regulation of intracellular estrogen receptor signaling pathway	1/38	10/11590	0,032	0,439	0,414	WBP2	1
GO:0034370	triglyceride-rich lipoprotein particle remodeling	1/38	10/11590	0,032	0,439	0,414	APOE	1
GO:0034372	very-low-density lipoprotein particle remodeling	1/38	10/11590	0,032	0,439	0,414	APOE	1
GO:0034384	high-density lipoprotein particle clearance	1/38	10/11590	0,032	0,439	0,414	APOE	1
GO:0060623	regulation of chromosome condensation	1/38	10/11590	0,032	0,439	0,414	H3-3A	1
GO:1901030	positive regulation of mitochondrial outer membrane permeabilization involved in apoptotic signaling pathway	1/38	10/11590	0,032	0,439	0,414	NMT1	1
GO:1903365	regulation of fear response	1/38	10/11590	0,032	0,439	0,414	APOE	1
GO:1903961	positive regulation of anion transmembrane transport	1/38	10/11590	0,032	0,439	0,414	SLC38A1	1
GO:0015918	sterol transport	2/38	90/11590	0,035	0,439	0,414	APOE/APOC1	2
GO:0033145	positive regulation of intracellular steroid hormone receptor signaling pathway	1/38	11/11590	0,035	0,439	0,414	WBP2	1
GO:0042159	lipoprotein catabolic process	1/38	11/11590	0,035	0,439	0,414	APOE	1
GO:0045815	epigenetic maintenance of chromatin in transcription-competent conformation	1/38	11/11590	0,035	0,439	0,414	WBP2	1
GO:0090205	positive regulation of cholesterol metabolic process	1/38	11/11590	0,035	0,439	0,414	APOE	1
GO:1900272	negative regulation of long-term synaptic potentiation	1/38	11/11590	0,035	0,439	0,414	APOE	1
GO:1902065	response to L-glutamate	1/38	11/11590	0,035	0,439	0,414	UFL1	1
GO:1902950	regulation of dendritic spine maintenance	1/38	11/11590	0,035	0,439	0,414	APOE	1
GO:0006639	acylglycerol metabolic process	2/38	93/11590	0,037	0,439	0,414	APOE/APOC1	2
GO:0006638	neutral lipid metabolic process	2/38	94/11590	0,038	0,439	0,414	APOE/APOC1	2
GO:0071901	negative regulation of protein serine/threonine kinase activity	2/38	95/11590	0,039	0,439	0,414	APOE/HEXIM 1	2

GO:0009886	post-embryonic animal morphogenesis	1/38	12/11590	0,039	0,439	0,414	EFEMP1	1
GO:0032328	alanine transport	1/38	12/11590	0,039	0,439	0,414	SLC38A1	1
GO:0032488	Cdc42 protein signal transduction	1/38	12/11590	0,039	0,439	0,414	APOE	1
GO:0033540	fatty acid beta-oxidation using acyl-CoA oxidase	1/38	12/11590	0,039	0,439	0,414	ACOX1	1
GO:0034380	high-density lipoprotein particle assembly	1/38	12/11590	0,039	0,439	0,414	APOE	1
GO:0034638	phosphatidylcholine catabolic process	1/38	12/11590	0,039	0,439	0,414	APOC1	1
GO:0050665	hydrogen peroxide biosynthetic process	1/38	12/11590	0,039	0,439	0,414	ACOX1	1
GO:0051044	positive regulation of membrane protein ectodomain proteolysis	1/38	12/11590	0,039	0,439	0,414	APOE	1
GO:0071391	cellular response to estrogen stimulus	1/38	12/11590	0,039	0,439	0,414	WBP2	1
GO:1900102	negative regulation of endoplasmic reticulum unfolded protein response	1/38	12/11590	0,039	0,439	0,414	UFL1	1
GO:1900452	regulation of long-term synaptic depression	1/38	12/11590	0,039	0,439	0,414	STAU2	1
GO:1905907	negative regulation of amyloid fibril formation	1/38	12/11590	0,039	0,439	0,414	APOE	1
GO:2001140	positive regulation of phospholipid transport	1/38	12/11590	0,039	0,439	0,414	APOE	1
GO:0016056	rhodopsin mediated signaling pathway	1/38	13/11590	0,042	0,439	0,414	NMT1	1
GO:0035641	locomotory exploration behavior	1/38	13/11590	0,042	0,439	0,414	APOE	1
GO:0055089	fatty acid homeostasis	1/38	13/11590	0,042	0,439	0,414	APOE	1
GO:0060192	negative regulation of lipase activity	1/38	13/11590	0,042	0,439	0,414	APOC1	1
GO:0098935	dendritic transport	1/38	13/11590	0,042	0,439	0,414	STAU2	1
GO:1902931	negative regulation of alcohol biosynthetic process	1/38	13/11590	0,042	0,439	0,414	APOE	1
GO:1903894	regulation of IRE1-mediated unfolded protein response	1/38	13/11590	0,042	0,439	0,414	UFL1	1
GO:2001138	regulation of phospholipid transport	1/38	13/11590	0,042	0,439	0,414	APOE	1
GO:0015748	organophosphate ester transport	2/38	101/11590	0,043	0,439	0,414	APOE/APOC1	2
GO:0030518	intracellular steroid hormone receptor signaling pathway	2/38	103/11590	0,045	0,439	0,414	UFL1/WBP2	2
GO:0032897	negative regulation of viral transcription	1/38	14/11590	0,045	0,439	0,414	HEXIM1	1

GO:0044794	positive regulation by host of viral process	1/38	14/11590	0,045	0,439	0,414	APOE	1
GO:1905906	regulation of amyloid fibril formation	1/38	14/11590	0,045	0,439	0,414	APOE	1
GO:0016042	lipid catabolic process	3/38	246/11590	0,046	0,439	0,414	APOE/APOC1/ ACOX1	3
GO:0010958	regulation of amino acid import across plasma membrane	1/38	15/11590	0,048	0,439	0,414	SLC38A1	1
GO:0015936	coenzyme A metabolic process	1/38	15/11590	0,048	0,439	0,414	DCAKD	1
GO:0032211	negative regulation of telomere maintenance via telomerase	1/38	15/11590	0,048	0,439	0,414	STN1	1
GO:0034244	negative regulation of transcription elongation by RNA polymerase II	1/38	15/11590	0,048	0,439	0,414	HEXIM1	1
GO:0034374	low-density lipoprotein particle remodeling	1/38	15/11590	0,048	0,439	0,414	APOE	1
GO:0043691	reverse cholesterol transport	1/38	15/11590	0,048	0,439	0,414	APOE	1
GO:0051004	regulation of lipoprotein lipase activity	1/38	15/11590	0,048	0,439	0,414	APOC1	1
GO:0061003	positive regulation of dendritic spine morphogenesis	1/38	15/11590	0,048	0,439	0,414	STAU2	1
GO:0061709	reticulophagy	1/38	15/11590	0,048	0,439	0,414	UFL1	1
GO:1903789	regulation of amino acid transmembrane transport	1/38	15/11590	0,048	0,439	0,414	SLC38A1	1
GO:0032368	regulation of lipid transport	2/38	110/11590	0,050	0,439	0,414	APOE/APOC1	2
GO:0045717	negative regulation of fatty acid biosynthetic process	1/38	16/11590	0,051	0,439	0,414	APOC1	1
GO:0048569	post-embryonic animal organ development	1/38	16/11590	0,051	0,439	0,414	EFEMP1	1
GO:1900221	regulation of amyloid-beta clearance	1/38	16/11590	0,051	0,439	0,414	APOE	1
GO:0010544	negative regulation of platelet activation	1/38	17/11590	0,054	0,439	0,414	APOE	1
GO:0010984	regulation of lipoprotein particle clearance	1/38	17/11590	0,054	0,439	0,414	APOC1	1
GO:0043117	positive regulation of vascular permeability	1/38	17/11590	0,054	0,439	0,414	APOE	1
GO:0061620	glycolytic process through glucose-6-phosphate	1/38	17/11590	0,054	0,439	0,414	GALK1	1
GO:1902430	negative regulation of amyloid-beta formation	1/38	17/11590	0,054	0,439	0,414	APOE	1
GO:0007266	Rho protein signal transduction	2/38	115/11590	0,054	0,439	0,414	APOE/ARHGE F12	2

GO:0008203	cholesterol metabolic process	2/38	118/11590	0,057	0,439	0,414	APOE/APOC1	2
GO:0007603	phototransduction, visible light	1/38	18/11590	0,057	0,439	0,414	NMT1	1
GO:0019400	alditol metabolic process	1/38	18/11590	0,057	0,439	0,414	GALK1	1
GO:0032785	negative regulation of DNA-templated transcription, elongation	1/38	18/11590	0,057	0,439	0,414	HEXIM1	1
GO:0044154	histone H3-K14 acetylation	1/38	18/11590	0,057	0,439	0,414	WBP2	1
GO:0045540	regulation of cholesterol biosynthetic process	1/38	18/11590	0,057	0,439	0,414	APOE	1
GO:0046475	glycerophospholipid catabolic process	1/38	18/11590	0,057	0,439	0,414	APOC1	1
GO:0046782	regulation of viral transcription	1/38	18/11590	0,057	0,439	0,414	HEXIM1	1
GO:0060252	positive regulation of glial cell proliferation	1/38	18/11590	0,057	0,439	0,414	UFL1	1
GO:0061615	glycolytic process through fructose-6-phosphate	1/38	18/11590	0,057	0,439	0,414	GALK1	1
GO:0106118	regulation of sterol biosynthetic process	1/38	18/11590	0,057	0,439	0,414	APOE	1
GO:0043401	steroid hormone mediated signaling pathway	2/38	120/11590	0,059	0,439	0,414	UFL1/WBP2	2
GO:0055088	lipid homeostasis	2/38	121/11590	0,060	0,439	0,414	APOE/ACOX1	2
GO:0036498	IRE1-mediated unfolded protein response	1/38	19/11590	0,061	0,439	0,414	UFL1	1
GO:0050995	negative regulation of lipid catabolic process	1/38	19/11590	0,061	0,439	0,414	APOC1	1
GO:0051000	positive regulation of nitric-oxide synthase activity	1/38	19/11590	0,061	0,439	0,414	APOE	1
GO:0097062	dendritic spine maintenance	1/38	19/11590	0,061	0,439	0,414	APOE	1
GO:1904357	negative regulation of telomere maintenance via telomere lengthening	1/38	19/11590	0,061	0,439	0,414	STN1	1
GO:0019216	regulation of lipid metabolic process	3/38	276/11590	0,061	0,439	0,414	MTMR9/APOE /APOC1	3
GO:0040029	epigenetic regulation of gene expression	2/38	123/11590	0,061	0,439	0,414	WBP2/H3-3A	2
GO:0032331	negative regulation of chondrocyte differentiation	1/38	20/11590	0,064	0,439	0,414	EFEMP1	1
GO:0051043	regulation of membrane protein ectodomain proteolysis	1/38	20/11590	0,064	0,439	0,414	APOE	1
GO:1901028	regulation of mitochondrial outer membrane permeabilization involved in apoptotic signaling pathway	1/38	20/11590	0,064	0,439	0,414	NMT1	1

GO:1902992	negative regulation of amyloid precursor protein catabolic process	1/38	20/11590	0,064	0,439	0,414	APOE	1
GO:1902652	secondary alcohol metabolic process	2/38	126/11590	0,064	0,439	0,414	APOE/APOC1	2
GO:0032200	telomere organization	2/38	128/11590	0,066	0,439	0,414	STN1/H3-3A	2
GO:0045834	positive regulation of lipid metabolic process	2/38	128/11590	0,066	0,439	0,414	APOE/APOC1	2
GO:0016125	sterol metabolic process	2/38	129/11590	0,067	0,439	0,414	APOE/APOC1	2
GO:1903959	regulation of anion transmembrane transport	1/38	21/11590	0,067	0,439	0,414	SLC38A1	1
GO:0099173	postsynapse organization	2/38	131/11590	0,068	0,439	0,414	STAU2/APOE	2
GO:0006541	glutamine metabolic process	1/38	22/11590	0,070	0,439	0,414	SLC38A1	1
GO:0010894	negative regulation of steroid biosynthetic process	1/38	22/11590	0,070	0,439	0,414	APOE	1
GO:0031365	N-terminal protein amino acid modification	1/38	22/11590	0,070	0,439	0,414	NMT1	1
GO:0032801	receptor catabolic process	1/38	22/11590	0,070	0,439	0,414	APOE	1
GO:0046835	carbohydrate phosphorylation	1/38	22/11590	0,070	0,439	0,414	GALK1	1
GO:0048261	negative regulation of receptor-mediated endocytosis	1/38	22/11590	0,070	0,439	0,414	APOC1	1
GO:0051957	positive regulation of amino acid transport	1/38	22/11590	0,070	0,439	0,414	SLC38A1	1
GO:0150146	cell junction disassembly	1/38	22/11590	0,070	0,439	0,414	STON1	1
GO:1905952	regulation of lipid localization	2/38	133/11590	0,070	0,439	0,414	APOE/APOC1	2
GO:0006897	endocytosis	4/38	480/11590	0,071	0,439	0,414	MTMR9/APOE/APOC1/STON1	4
GO:0010976	positive regulation of neuron projection development	2/38	134/11590	0,071	0,439	0,414	STAU2/APOE	2
GO:0006929	substrate-dependent cell migration	1/38	23/11590	0,073	0,439	0,414	STON1	1
GO:0010875	positive regulation of cholesterol efflux	1/38	23/11590	0,073	0,439	0,414	APOE	1
GO:0032369	negative regulation of lipid transport	1/38	23/11590	0,073	0,439	0,414	APOC1	1
GO:0070199	establishment of protein localization to chromosome	1/38	23/11590	0,073	0,439	0,414	WBP2	1
GO:0046486	glycerolipid metabolic process	3/38	297/11590	0,073	0,439	0,414	MTMR9/APOE/APOC1	3

GO:0048592	eye morphogenesis	2/38	136/11590	0,073	0,439	0,414	STAU2/EFEMP1	2
GO:1905475	regulation of protein localization to membrane	2/38	136/11590	0,073	0,439	0,414	NMT1/GPC5	2
GO:0006066	alcohol metabolic process	3/38	302/11590	0,076	0,439	0,414	GALK1/APOE/APOC1	3
GO:0006891	intra-Golgi vesicle-mediated transport	1/38	24/11590	0,076	0,439	0,414	COG2	1
GO:0035640	exploration behavior	1/38	24/11590	0,076	0,439	0,414	APOE	1
GO:0045939	negative regulation of steroid metabolic process	1/38	24/11590	0,076	0,439	0,414	APOE	1
GO:1900101	regulation of endoplasmic reticulum unfolded protein response	1/38	24/11590	0,076	0,439	0,414	UFL1	1
GO:0006631	fatty acid metabolic process	3/38	303/11590	0,076	0,439	0,414	APOC1/ACOX1/ACBD4	3
GO:0046890	regulation of lipid biosynthetic process	2/38	141/11590	0,078	0,439	0,414	APOE/APOC1	2
GO:0019068	virion assembly	1/38	25/11590	0,079	0,439	0,414	APOE	1
GO:0034508	centromere complex assembly	1/38	25/11590	0,079	0,439	0,414	H3-3A	1
GO:0045736	negative regulation of cyclin-dependent protein serine/threonine kinase activity	1/38	25/11590	0,079	0,439	0,414	HEXIM1	1
GO:0055090	acylglycerol homeostasis	1/38	25/11590	0,079	0,439	0,414	APOE	1
GO:0060292	long-term synaptic depression	1/38	25/11590	0,079	0,439	0,414	STAU2	1
GO:0061037	negative regulation of cartilage development	1/38	25/11590	0,079	0,439	0,414	EFEMP1	1
GO:0070328	triglyceride homeostasis	1/38	25/11590	0,079	0,439	0,414	APOE	1
GO:1901798	positive regulation of signal transduction by p53 class mediator	1/38	25/11590	0,079	0,439	0,414	HEXIM1	1
GO:1904030	negative regulation of cyclin-dependent protein kinase activity	1/38	25/11590	0,079	0,439	0,414	HEXIM1	1
GO:0000038	very long-chain fatty acid metabolic process	1/38	26/11590	0,082	0,440	0,415	ACOX1	1
GO:0002021	response to dietary excess	1/38	26/11590	0,082	0,440	0,415	APOE	1
GO:0006706	steroid catabolic process	1/38	26/11590	0,082	0,440	0,415	APOE	1
GO:0007263	nitric oxide mediated signal transduction	1/38	26/11590	0,082	0,440	0,415	APOE	1
GO:0051204	protein insertion into mitochondrial membrane	1/38	26/11590	0,082	0,440	0,415	NMT1	1

GO:0044282	small molecule catabolic process	3/38	313/11590	0,082	0,440	0,415	GALK1/APOE/ ACOX1	3
GO:0001556	oocyte maturation	1/38	27/11590	0,085	0,440	0,415	H3-3A	1
GO:0007271	synaptic transmission, cholinergic	1/38	27/11590	0,085	0,440	0,415	APOE	1
GO:0010922	positive regulation of phosphatase activity	1/38	27/11590	0,085	0,440	0,415	MTMR9	1
GO:0032205	negative regulation of telomere maintenance	1/38	27/11590	0,085	0,440	0,415	STN1	1
GO:0001941	postsynaptic membrane organization	1/38	28/11590	0,088	0,440	0,415	APOE	1
GO:0046856	phosphatidylinositol dephosphorylation	1/38	28/11590	0,088	0,440	0,415	MTMR9	1
GO:0055094	response to lipoprotein particle	1/38	28/11590	0,088	0,440	0,415	APOE	1
GO:0090181	regulation of cholesterol metabolic process	1/38	28/11590	0,088	0,440	0,415	APOE	1
GO:0090207	regulation of triglyceride metabolic process	1/38	28/11590	0,088	0,440	0,415	APOE	1
GO:0061136	regulation of proteasomal protein catabolic process	2/38	152/11590	0,088	0,440	0,415	UFL1/APOE	2
GO:0010874	regulation of cholesterol efflux	1/38	29/11590	0,091	0,440	0,415	APOE	1
GO:0032770	positive regulation of monooxygenase activity	1/38	29/11590	0,091	0,440	0,415	APOE	1
GO:0045922	negative regulation of fatty acid metabolic process	1/38	29/11590	0,091	0,440	0,415	APOC1	1
GO:0050775	positive regulation of dendrite morphogenesis	1/38	29/11590	0,091	0,440	0,415	STAU2	1
GO:0090151	establishment of protein localization to mitochondrial membrane	1/38	29/11590	0,091	0,440	0,415	NMT1	1
GO:0007616	long-term memory	1/38	30/11590	0,094	0,440	0,415	APOE	1
GO:0032228	regulation of synaptic transmission, GABAergic	1/38	30/11590	0,094	0,440	0,415	SLC38A1	1
GO:0097345	mitochondrial outer membrane permeabilization	1/38	30/11590	0,094	0,440	0,415	NMT1	1
GO:2000279	negative regulation of DNA biosynthetic process	1/38	30/11590	0,094	0,440	0,415	STN1	1
GO:0050807	regulation of synapse organization	2/38	158/11590	0,094	0,440	0,415	STAU2/APOE	2
GO:0032373	positive regulation of sterol transport	1/38	31/11590	0,097	0,440	0,415	APOE	1
GO:0032376	positive regulation of cholesterol transport	1/38	31/11590	0,097	0,440	0,415	APOE	1
GO:0071168	protein localization to chromatin	1/38	31/11590	0,097	0,440	0,415	WBP2	1

GO:0071402	cellular response to lipoprotein particle stimulus	1/38	31/11590	0,097	0,440	0,415	APOE	1
GO:1903749	positive regulation of establishment of protein localization to mitochondrion	1/38	31/11590	0,097	0,440	0,415	NMT1	1
GO:0050803	regulation of synapse structure or activity	2/38	161/11590	0,097	0,440	0,415	STAU2/APOE	2
GO:0044242	cellular lipid catabolic process	2/38	163/11590	0,100	0,440	0,415	APOC1/ACOX1	2
GO:0018149	peptide cross-linking	1/38	32/11590	0,100	0,440	0,415	EVPL	1
GO:1902110	positive regulation of mitochondrial membrane permeability involved in apoptotic process	1/38	32/11590	0,100	0,440	0,415	NMT1	1
GO:0009755	hormone-mediated signaling pathway	2/38	164/11590	0,101	0,440	0,415	UFL1/WBP2	2
GO:0045936	negative regulation of phosphate metabolic process	3/38	342/11590	0,101	0,440	0,415	APOE/APOC1/HEXIM1	3
GO:0010563	negative regulation of phosphorus metabolic process	3/38	343/11590	0,102	0,440	0,415	APOE/APOC1/HEXIM1	3
GO:0009395	phospholipid catabolic process	1/38	33/11590	0,103	0,440	0,415	APOC1	1
GO:0019934	cGMP-mediated signaling	1/38	33/11590	0,103	0,440	0,415	APOE	1
GO:0035066	positive regulation of histone acetylation	1/38	33/11590	0,103	0,440	0,415	WBP2	1
GO:0043537	negative regulation of blood vessel endothelial cell migration	1/38	33/11590	0,103	0,440	0,415	APOE	1
GO:0048048	embryonic eye morphogenesis	1/38	33/11590	0,103	0,440	0,415	EFEMP1	1
GO:0050999	regulation of nitric-oxide synthase activity	1/38	33/11590	0,103	0,440	0,415	APOE	1
GO:0060218	hematopoietic stem cell differentiation	1/38	33/11590	0,103	0,440	0,415	UFL1	1
GO:0061001	regulation of dendritic spine morphogenesis	1/38	33/11590	0,103	0,440	0,415	STAU2	1
GO:1902003	regulation of amyloid-beta formation	1/38	33/11590	0,103	0,440	0,415	APOE	1
GO:0006469	negative regulation of protein kinase activity	2/38	167/11590	0,104	0,440	0,415	APOE/HEXIM1	2
GO:0007565	female pregnancy	2/38	168/11590	0,105	0,440	0,415	SLC38A1/H3-3A	2
GO:0042554	superoxide anion generation	1/38	34/11590	0,106	0,440	0,415	SH3PXD2A	1
GO:0060251	regulation of glial cell proliferation	1/38	34/11590	0,106	0,440	0,415	UFL1	1

GO:1902686	mitochondrial outer membrane permeabilization involved in programmed cell death	1/38	34/11590	0,106	0,440	0,415	NMT1	1
GO:1903573	negative regulation of response to endoplasmic reticulum stress	1/38	34/11590	0,106	0,440	0,415	UFL1	1
GO:1990000	amyloid fibril formation	1/38	34/11590	0,106	0,440	0,415	APOE	1
GO:0001503	ossification	3/38	352/11590	0,108	0,440	0,415	UFL1/VCAN/ H3-3A	3
GO:0001662	behavioral fear response	1/38	35/11590	0,109	0,440	0,415	APOE	1
GO:0045740	positive regulation of DNA replication	1/38	35/11590	0,109	0,440	0,415	STN1	1
GO:0097242	amyloid-beta clearance	1/38	35/11590	0,109	0,440	0,415	APOE	1
GO:1902108	regulation of mitochondrial membrane permeability involved in apoptotic process	1/38	35/11590	0,109	0,440	0,415	NMT1	1
GO:0050804	modulation of chemical synaptic transmission	3/38	354/11590	0,109	0,440	0,415	STAU2/SLC38 A1/APOE	3
GO:0099177	regulation of trans-synaptic signaling	3/38	355/11590	0,110	0,440	0,415	STAU2/SLC38 A1/APOE	3
GO:0002209	behavioral defense response	1/38	36/11590	0,112	0,440	0,415	APOE	1
GO:0031424	keratinization	1/38	36/11590	0,112	0,440	0,415	EVPL	1
GO:0032570	response to progesterone	1/38	36/11590	0,112	0,440	0,415	WBP2	1
GO:0046839	phospholipid dephosphorylation	1/38	36/11590	0,112	0,440	0,415	MTMR9	1
GO:0042180	cellular ketone metabolic process	2/38	176/11590	0,113	0,440	0,415	APOC1/NMT1	2
GO:0001937	negative regulation of endothelial cell proliferation	1/38	37/11590	0,115	0,440	0,415	APOE	1
GO:0007602	phototransduction	1/38	37/11590	0,115	0,440	0,415	NMT1	1
GO:0016233	telomere capping	1/38	37/11590	0,115	0,440	0,415	STN1	1
GO:0019320	hexose catabolic process	1/38	37/11590	0,115	0,440	0,415	GALK1	1
GO:0035794	positive regulation of mitochondrial membrane permeability	1/38	37/11590	0,115	0,440	0,415	NMT1	1
GO:0044788	modulation by host of viral process	1/38	37/11590	0,115	0,440	0,415	APOE	1
GO:0045806	negative regulation of endocytosis	1/38	37/11590	0,115	0,440	0,415	APOC1	1

GO:1903050	regulation of proteolysis involved in protein catabolic process	2/38	178/11590	0,115	0,440	0,415	UFL1/APOE	2
GO:0071383	cellular response to steroid hormone stimulus	2/38	179/11590	0,116	0,440	0,415	UFL1/WBP2	2
GO:0006509	membrane protein ectodomain proteolysis	1/38	38/11590	0,117	0,440	0,415	APOE	1
GO:0006692	prostanoid metabolic process	1/38	38/11590	0,117	0,440	0,415	ACOX1	1
GO:0006693	prostaglandin metabolic process	1/38	38/11590	0,117	0,440	0,415	ACOX1	1
GO:0009584	detection of visible light	1/38	38/11590	0,117	0,440	0,415	NMT1	1
GO:0097178	ruffle assembly	1/38	38/11590	0,117	0,440	0,415	STON1	1
GO:1900271	regulation of long-term synaptic potentiation	1/38	38/11590	0,117	0,440	0,415	APOE	1
GO:1905953	negative regulation of lipid localization	1/38	38/11590	0,117	0,440	0,415	APOC1	1
GO:2000758	positive regulation of peptidyl-lysine acetylation	1/38	38/11590	0,117	0,440	0,415	WBP2	1
GO:0042304	regulation of fatty acid biosynthetic process	1/38	39/11590	0,120	0,440	0,415	APOC1	1
GO:0046365	monosaccharide catabolic process	1/38	39/11590	0,120	0,440	0,415	GALK1	1
GO:0051954	positive regulation of amine transport	1/38	39/11590	0,120	0,440	0,415	SLC38A1	1
GO:0120009	intermembrane lipid transfer	1/38	39/11590	0,120	0,440	0,415	APOE	1
GO:1902991	regulation of amyloid precursor protein catabolic process	1/38	39/11590	0,120	0,440	0,415	APOE	1
GO:0042743	hydrogen peroxide metabolic process	1/38	40/11590	0,123	0,440	0,415	ACOX1	1
GO:0051955	regulation of amino acid transport	1/38	40/11590	0,123	0,440	0,415	SLC38A1	1
GO:0033673	negative regulation of kinase activity	2/38	186/11590	0,124	0,440	0,415	APOE/HEXIM 1	2
GO:0044703	multi-organism reproductive process	2/38	186/11590	0,124	0,440	0,415	SLC38A1/H3- 3A	2
GO:0060828	regulation of canonical Wnt signaling pathway	2/38	188/11590	0,126	0,440	0,415	APOE/GPC5	2
GO:0015804	neutral amino acid transport	1/38	41/11590	0,126	0,440	0,415	SLC38A1	1
GO:0030261	chromosome condensation	1/38	41/11590	0,126	0,440	0,415	H3-3A	1
GO:0032892	positive regulation of organic acid transport	1/38	41/11590	0,126	0,440	0,415	SLC38A1	1
GO:0034205	amyloid-beta formation	1/38	41/11590	0,126	0,440	0,415	APOE	1
GO:0042596	fear response	1/38	41/11590	0,126	0,440	0,415	APOE	1

GO:1905710	positive regulation of membrane permeability	1/38	41/11590	0,126	0,440	0,415	NMT1	1
GO:0072593	reactive oxygen species metabolic process	2/38	190/11590	0,128	0,440	0,415	SH3PXD2A/A COX1	2
GO:0001504	neurotransmitter uptake	1/38	42/11590	0,129	0,440	0,415	SLC38A1	1
GO:1903747	regulation of establishment of protein localization to mitochondrion	1/38	42/11590	0,129	0,440	0,415	NMT1	1
GO:0031330	negative regulation of cellular catabolic process	2/38	192/11590	0,130	0,440	0,415	MTMR9/APO C1	2
GO:0044706	multi-multicellular organism process	2/38	192/11590	0,130	0,440	0,415	SLC38A1/H3-3A	2
GO:0045088	regulation of innate immune response	2/38	193/11590	0,132	0,440	0,415	APOE/HEXIM 1	2
GO:0032210	regulation of telomere maintenance via telomerase	1/38	43/11590	0,132	0,440	0,415	STN1	1
GO:0032330	regulation of chondrocyte differentiation	1/38	43/11590	0,132	0,440	0,415	EFEMP1	1
GO:0046503	glycerolipid catabolic process	1/38	43/11590	0,132	0,440	0,415	APOC1	1
GO:0051965	positive regulation of synapse assembly	1/38	43/11590	0,132	0,440	0,415	STAU2	1
GO:0016358	dendrite development	2/38	194/11590	0,133	0,440	0,415	STAU2/APOE	2
GO:0007281	germ cell development	2/38	195/11590	0,134	0,440	0,415	STAU2/H3-3A	2
GO:0051489	regulation of filopodium assembly	1/38	44/11590	0,135	0,440	0,415	STAU2	1
GO:1903793	positive regulation of anion transport	1/38	44/11590	0,135	0,440	0,415	SLC38A1	1
GO:0032768	regulation of monooxygenase activity	1/38	45/11590	0,138	0,440	0,415	APOE	1
GO:0043114	regulation of vascular permeability	1/38	45/11590	0,138	0,440	0,415	APOE	1
GO:0048168	regulation of neuronal synaptic plasticity	1/38	45/11590	0,138	0,440	0,415	APOE	1
GO:0089718	amino acid import across plasma membrane	1/38	45/11590	0,138	0,440	0,415	SLC38A1	1
GO:0050769	positive regulation of neurogenesis	2/38	199/11590	0,138	0,440	0,415	UFL1/STAU2	2
GO:0033044	regulation of chromosome organization	2/38	200/11590	0,139	0,440	0,415	STN1/H3-3A	2
GO:0007566	embryo implantation	1/38	46/11590	0,140	0,440	0,415	H3-3A	1
GO:0010543	regulation of platelet activation	1/38	46/11590	0,140	0,440	0,415	APOE	1
GO:0014009	glial cell proliferation	1/38	46/11590	0,140	0,440	0,415	UFL1	1
GO:0042311	vasodilation	1/38	46/11590	0,140	0,440	0,415	APOE	1

GO:0043113	receptor clustering	1/38	46/11590	0,140	0,440	0,415	APOE	1
GO:0046902	regulation of mitochondrial membrane permeability	1/38	46/11590	0,140	0,440	0,415	NMT1	1
GO:0048599	oocyte development	1/38	46/11590	0,140	0,440	0,415	H3-3A	1
GO:1901985	positive regulation of protein acetylation	1/38	46/11590	0,140	0,440	0,415	WBP2	1
GO:1903409	reactive oxygen species biosynthetic process	1/38	46/11590	0,140	0,440	0,415	ACOX1	1
GO:0031400	negative regulation of protein modification process	3/38	398/11590	0,141	0,440	0,415	UFL1/APOE/HEXIM1	3
GO:0006354	DNA-templated transcription elongation	2/38	202/11590	0,142	0,440	0,415	FHL5/HEXIM1	2
GO:0006695	cholesterol biosynthetic process	1/38	47/11590	0,143	0,440	0,415	APOE	1
GO:0009994	oocyte differentiation	1/38	47/11590	0,143	0,440	0,415	H3-3A	1
GO:0030195	negative regulation of blood coagulation	1/38	47/11590	0,143	0,440	0,415	APOE	1
GO:0033619	membrane protein proteolysis	1/38	47/11590	0,143	0,440	0,415	APOE	1
GO:0046164	alcohol catabolic process	1/38	47/11590	0,143	0,440	0,415	APOE	1
GO:0060997	dendritic spine morphogenesis	1/38	47/11590	0,143	0,440	0,415	STAU2	1
GO:1902653	secondary alcohol biosynthetic process	1/38	47/11590	0,143	0,440	0,415	APOE	1
GO:1902930	regulation of alcohol biosynthetic process	1/38	47/11590	0,143	0,440	0,415	APOE	1
GO:1904356	regulation of telomere maintenance via telomere lengthening	1/38	47/11590	0,143	0,440	0,415	STN1	1
GO:0010596	negative regulation of endothelial cell migration	1/38	48/11590	0,146	0,440	0,415	APOE	1
GO:0019083	viral transcription	1/38	48/11590	0,146	0,440	0,415	HEXIM1	1
GO:0035306	positive regulation of dephosphorylation	1/38	48/11590	0,146	0,440	0,415	MTMR9	1
GO:0048662	negative regulation of smooth muscle cell proliferation	1/38	48/11590	0,146	0,440	0,415	APOE	1
GO:0050709	negative regulation of protein secretion	1/38	48/11590	0,146	0,440	0,415	APOE	1
GO:0050994	regulation of lipid catabolic process	1/38	48/11590	0,146	0,440	0,415	APOC1	1
GO:0051932	synaptic transmission, GABAergic	1/38	48/11590	0,146	0,440	0,415	SLC38A1	1
GO:1900047	negative regulation of hemostasis	1/38	48/11590	0,146	0,440	0,415	APOE	1
GO:0043543	protein acylation	2/38	208/11590	0,148	0,440	0,415	WBP2/NMT1	2

GO:0051348	negative regulation of transferase activity	2/38	208/11590	0,148	0,440	0,415	APOE/HEXIM 1	2
GO:0009583	detection of light stimulus	1/38	49/11590	0,149	0,440	0,415	NMT1	1
GO:0043112	receptor metabolic process	1/38	49/11590	0,149	0,440	0,415	APOE	1
GO:0043407	negative regulation of MAP kinase activity	1/38	49/11590	0,149	0,440	0,415	APOE	1
GO:0050819	negative regulation of coagulation	1/38	49/11590	0,149	0,440	0,415	APOE	1
GO:0000768	syncytium formation by plasma membrane fusion	1/38	50/11590	0,152	0,440	0,415	SH3PXD2A	1
GO:0002218	activation of innate immune response	1/38	50/11590	0,152	0,440	0,415	HEXIM1	1
GO:0031529	ruffle organization	1/38	50/11590	0,152	0,440	0,415	STON1	1
GO:0033866	nucleoside bisphosphate biosynthetic process	1/38	50/11590	0,152	0,440	0,415	DCAKD	1
GO:0034030	ribonucleoside bisphosphate biosynthetic process	1/38	50/11590	0,152	0,440	0,415	DCAKD	1
GO:0034033	purine nucleoside bisphosphate biosynthetic process	1/38	50/11590	0,152	0,440	0,415	DCAKD	1
GO:0050435	amyloid-beta metabolic process	1/38	50/11590	0,152	0,440	0,415	APOE	1
GO:0051205	protein insertion into membrane	1/38	50/11590	0,152	0,440	0,415	NMT1	1
GO:0140253	cell-cell fusion	1/38	50/11590	0,152	0,440	0,415	SH3PXD2A	1
GO:0051353	positive regulation of oxidoreductase activity	1/38	51/11590	0,155	0,446	0,421	APOE	1
GO:0006898	receptor-mediated endocytosis	2/38	214/11590	0,155	0,446	0,421	APOE/APOC1	2
GO:0015850	organic hydroxy compound transport	2/38	214/11590	0,155	0,446	0,421	APOE/APOC1	2
GO:0090596	sensory organ morphogenesis	2/38	215/11590	0,156	0,447	0,421	STAU2/EFEM P1	2
GO:0042987	amyloid precursor protein catabolic process	1/38	52/11590	0,157	0,447	0,421	APOE	1
GO:0043954	cellular component maintenance	1/38	52/11590	0,157	0,447	0,421	APOE	1
GO:0048008	platelet-derived growth factor receptor signaling pathway	1/38	52/11590	0,157	0,447	0,421	STON1	1
GO:0006949	syncytium formation	1/38	53/11590	0,160	0,451	0,425	SH3PXD2A	1
GO:0048488	synaptic vesicle endocytosis	1/38	53/11590	0,160	0,451	0,425	STON1	1
GO:0140238	presynaptic endocytosis	1/38	53/11590	0,160	0,451	0,425	STON1	1
GO:0016126	sterol biosynthetic process	1/38	54/11590	0,163	0,457	0,431	APOE	1

GO:0015909	long-chain fatty acid transport	1/38	55/11590	0,166	0,464	0,437	APOE	1
GO:0006801	superoxide metabolic process	1/38	56/11590	0,168	0,466	0,440	SH3PXD2A	1
GO:0035065	regulation of histone acetylation	1/38	56/11590	0,168	0,466	0,440	WBP2	1
GO:0046847	filopodium assembly	1/38	56/11590	0,168	0,466	0,440	STAU2	1
GO:0048814	regulation of dendrite morphogenesis	1/38	56/11590	0,168	0,466	0,440	STAU2	1
GO:0007004	telomere maintenance via telomerase	1/38	57/11590	0,171	0,470	0,443	STN1	1
GO:0035418	protein localization to synapse	1/38	57/11590	0,171	0,470	0,443	STAU2	1
GO:0006278	RNA-templated DNA biosynthetic process	1/38	58/11590	0,174	0,470	0,443	STN1	1
GO:0014015	positive regulation of gliogenesis	1/38	58/11590	0,174	0,470	0,443	UFL1	1
GO:0015807	L-amino acid transport	1/38	58/11590	0,174	0,470	0,443	SLC38A1	1
GO:0043627	response to estrogen	1/38	58/11590	0,174	0,470	0,443	WBP2	1
GO:0090559	regulation of membrane permeability	1/38	58/11590	0,174	0,470	0,443	NMT1	1
GO:0060627	regulation of vesicle-mediated transport	3/38	440/11590	0,174	0,470	0,443	APOE/APOC1/ STON1	3
GO:0051962	positive regulation of nervous system development	2/38	231/11590	0,175	0,470	0,443	UFL1/STAU2	2
GO:0030522	intracellular receptor signaling pathway	2/38	232/11590	0,176	0,470	0,443	UFL1/WBP2	2
GO:0060070	canonical Wnt signaling pathway	2/38	232/11590	0,176	0,470	0,443	APOE/GPC5	2
GO:0050805	negative regulation of synaptic transmission	1/38	59/11590	0,177	0,470	0,443	STAU2	1
GO:0061035	regulation of cartilage development	1/38	59/11590	0,177	0,470	0,443	EFEMP1	1
GO:1902475	L-alpha-amino acid transmembrane transport	1/38	59/11590	0,177	0,470	0,443	SLC38A1	1
GO:0006650	glycerophospholipid metabolic process	2/38	234/11590	0,178	0,473	0,446	MTMR9/APO C1	2
GO:0008088	axo-dendritic transport	1/38	60/11590	0,179	0,473	0,446	STAU2	1
GO:0050795	regulation of behavior	1/38	60/11590	0,179	0,473	0,446	APOE	1
GO:0072657	protein localization to membrane	3/38	447/11590	0,180	0,473	0,446	APOE/NMT1/ GPC5	3
GO:0010633	negative regulation of epithelial cell migration	1/38	61/11590	0,182	0,474	0,447	APOE	1
GO:0030968	endoplasmic reticulum unfolded protein response	1/38	61/11590	0,182	0,474	0,447	UFL1	1

GO:0046470	phosphatidylcholine metabolic process	1/38	61/11590	0,182	0,474	0,447	APOC1	1
GO:0050810	regulation of steroid biosynthetic process	1/38	61/11590	0,182	0,474	0,447	APOE	1
GO:0006334	nucleosome assembly	1/38	62/11590	0,185	0,476	0,449	H3-3A	1
GO:0009064	glutamine family amino acid metabolic process	1/38	62/11590	0,185	0,476	0,449	SLC38A1	1
GO:0010822	positive regulation of mitochondrion organization	1/38	62/11590	0,185	0,476	0,449	NMT1	1
GO:0036465	synaptic vesicle recycling	1/38	62/11590	0,185	0,476	0,449	STON1	1
GO:0051056	regulation of small GTPase mediated signal transduction	2/38	240/11590	0,186	0,477	0,450	APOE/ARHGE F12	2
GO:0010921	regulation of phosphatase activity	1/38	63/11590	0,187	0,477	0,450	MTMR9	1
GO:2000756	regulation of peptidyl-lysine acetylation	1/38	63/11590	0,187	0,477	0,450	WBP2	1
GO:0030111	regulation of Wnt signaling pathway	2/38	243/11590	0,189	0,477	0,450	APOE/GPC5	2
GO:0010833	telomere maintenance via telomere lengthening	1/38	64/11590	0,190	0,477	0,450	STN1	1
GO:0031507	heterochromatin formation	1/38	64/11590	0,190	0,477	0,450	H3-3A	1
GO:0003018	vascular process in circulatory system	2/38	244/11590	0,190	0,477	0,450	SLC38A1/APO E	2
GO:0006497	protein lipidation	1/38	65/11590	0,193	0,477	0,450	NMT1	1
GO:0006635	fatty acid beta-oxidation	1/38	65/11590	0,193	0,477	0,450	ACOX1	1
GO:0048844	artery morphogenesis	1/38	65/11590	0,193	0,477	0,450	APOE	1
GO:1901615	organic hydroxy compound metabolic process	3/38	463/11590	0,193	0,477	0,450	GALK1/APOE/ APOC1	3
GO:0015849	organic acid transport	2/38	248/11590	0,195	0,477	0,450	SLC38A1/APO E	2
GO:0008652	cellular amino acid biosynthetic process	1/38	66/11590	0,195	0,477	0,450	SLC38A1	1
GO:0019915	lipid storage	1/38	66/11590	0,195	0,477	0,450	APOE	1
GO:0030193	regulation of blood coagulation	1/38	66/11590	0,195	0,477	0,450	APOE	1
GO:0031397	negative regulation of protein ubiquitination	1/38	66/11590	0,195	0,477	0,450	UFL1	1
GO:0032088	negative regulation of NF-kappaB transcription factor activity	1/38	66/11590	0,195	0,477	0,450	UFL1	1

GO:0043966	histone H3 acetylation	1/38	66/11590	0,195	0,477	0,450	WBP2	1
GO:0061045	negative regulation of wound healing	1/38	66/11590	0,195	0,477	0,450	APOE	1
GO:1901616	organic hydroxy compound catabolic process	1/38	66/11590	0,195	0,477	0,450	APOE	1
GO:1905897	regulation of response to endoplasmic reticulum stress	1/38	66/11590	0,195	0,477	0,450	UFL1	1
GO:0046394	carboxylic acid biosynthetic process	2/38	250/11590	0,197	0,479	0,452	SLC38A1/APOC1	2
GO:0032890	regulation of organic acid transport	1/38	67/11590	0,198	0,479	0,452	SLC38A1	1
GO:0042982	amyloid precursor protein metabolic process	1/38	67/11590	0,198	0,479	0,452	APOE	1
GO:0061912	selective autophagy	1/38	67/11590	0,198	0,479	0,452	UFL1	1
GO:0044283	small molecule biosynthetic process	3/38	470/11590	0,199	0,480	0,453	SLC38A1/APOE/APOC1	3
GO:0016053	organic acid biosynthetic process	2/38	252/11590	0,200	0,481	0,453	SLC38A1/APOC1	2
GO:0051851	modulation by host of symbiont process	1/38	68/11590	0,201	0,481	0,453	APOE	1
GO:1900046	regulation of hemostasis	1/38	68/11590	0,201	0,481	0,453	APOE	1
GO:0034243	regulation of transcription elongation by RNA polymerase II	1/38	69/11590	0,203	0,484	0,456	HEXIM1	1
GO:0046889	positive regulation of lipid biosynthetic process	1/38	69/11590	0,203	0,484	0,456	APOE	1
GO:0050818	regulation of coagulation	1/38	69/11590	0,203	0,484	0,456	APOE	1
GO:0010507	negative regulation of autophagy	1/38	70/11590	0,206	0,484	0,457	MTMR9	1
GO:0042632	cholesterol homeostasis	1/38	70/11590	0,206	0,484	0,457	APOE	1
GO:0045104	intermediate filament cytoskeleton organization	1/38	70/11590	0,206	0,484	0,457	EVPL	1
GO:0048477	oogenesis	1/38	70/11590	0,206	0,484	0,457	H3-3A	1
GO:0001933	negative regulation of protein phosphorylation	2/38	259/11590	0,208	0,484	0,457	APOE/HEXIM1	2
GO:0009895	negative regulation of catabolic process	2/38	259/11590	0,208	0,484	0,457	MTMR9/APOC1	2
GO:0045103	intermediate filament-based process	1/38	71/11590	0,209	0,484	0,457	EVPL	1
GO:0055092	sterol homeostasis	1/38	71/11590	0,209	0,484	0,457	APOE	1

GO:0070828	heterochromatin organization	1/38	71/11590	0,209	0,484	0,457	H3-3A	1
GO:0098869	cellular oxidant detoxification	1/38	71/11590	0,209	0,484	0,457	APOE	1
GO:0010770	positive regulation of cell morphogenesis involved in differentiation	1/38	72/11590	0,211	0,486	0,458	STAU2	1
GO:0045814	negative regulation of gene expression, epigenetic	1/38	72/11590	0,211	0,486	0,458	H3-3A	1
GO:0051963	regulation of synapse assembly	1/38	72/11590	0,211	0,486	0,458	STAU2	1
GO:1901983	regulation of protein acetylation	1/38	72/11590	0,211	0,486	0,458	WBP2	1
GO:0033555	multicellular organismal response to stress	1/38	73/11590	0,214	0,489	0,462	APOE	1
GO:1903321	negative regulation of protein modification by small protein conjugation or removal	1/38	73/11590	0,214	0,489	0,462	UFL1	1
GO:0032370	positive regulation of lipid transport	1/38	74/11590	0,216	0,492	0,464	APOE	1
GO:0035023	regulation of Rho protein signal transduction	1/38	74/11590	0,216	0,492	0,464	APOE	1
GO:0060291	long-term synaptic potentiation	1/38	74/11590	0,216	0,492	0,464	APOE	1
GO:0007276	gamete generation	3/38	491/11590	0,217	0,492	0,464	STAU2/H3-3A/ACOX1	3
GO:0006338	chromatin remodeling	2/38	268/11590	0,219	0,495	0,467	WBP2/H3-3A	2
GO:0008202	steroid metabolic process	2/38	268/11590	0,219	0,495	0,467	APOE/APOC1	2
GO:0010720	positive regulation of cell development	2/38	269/11590	0,220	0,496	0,467	UFL1/STAU2	2
GO:0006096	glycolytic process	1/38	76/11590	0,222	0,496	0,467	GALK1	1
GO:0006757	ATP generation from ADP	1/38	76/11590	0,222	0,496	0,467	GALK1	1
GO:0062012	regulation of small molecule metabolic process	2/38	271/11590	0,223	0,496	0,467	APOE/APOC1	2
GO:0019217	regulation of fatty acid metabolic process	1/38	77/11590	0,224	0,496	0,467	APOC1	1
GO:0019935	cyclic-nucleotide-mediated signaling	1/38	77/11590	0,224	0,496	0,467	APOE	1
GO:0032204	regulation of telomere maintenance	1/38	77/11590	0,224	0,496	0,467	STN1	1
GO:0048041	focal adhesion assembly	1/38	77/11590	0,224	0,496	0,467	STON1	1
GO:1903035	negative regulation of response to wounding	1/38	77/11590	0,224	0,496	0,467	APOE	1
GO:0003333	amino acid transmembrane transport	1/38	78/11590	0,227	0,497	0,469	SLC38A1	1
GO:0034620	cellular response to unfolded protein	1/38	78/11590	0,227	0,497	0,469	UFL1	1

GO:0060191	regulation of lipase activity	1/38	78/11590	0,227	0,497	0,469	APOC1	1
GO:0022412	cellular process involved in reproduction in multicellular organism	2/38	275/11590	0,228	0,497	0,469	STAU2/H3-3A	2
GO:0010506	regulation of autophagy	2/38	276/11590	0,229	0,497	0,469	UFL1/MTMR9	2
GO:0015908	fatty acid transport	1/38	79/11590	0,229	0,497	0,469	APOE	1
GO:0030516	regulation of axon extension	1/38	79/11590	0,229	0,497	0,469	APOE	1
GO:0032092	positive regulation of protein binding	1/38	79/11590	0,229	0,497	0,469	APOE	1
GO:0044070	regulation of anion transport	1/38	79/11590	0,229	0,497	0,469	SLC38A1	1
GO:0009791	post-embryonic development	1/38	80/11590	0,232	0,500	0,472	EFEMP1	1
GO:0048525	negative regulation of viral process	1/38	80/11590	0,232	0,500	0,472	HEXIM1	1
GO:0009062	fatty acid catabolic process	1/38	81/11590	0,234	0,501	0,473	ACOX1	1
GO:0034502	protein localization to chromosome	1/38	81/11590	0,234	0,501	0,473	WBP2	1
GO:0045807	positive regulation of endocytosis	1/38	81/11590	0,234	0,501	0,473	APOE	1
GO:1905477	positive regulation of protein localization to membrane	1/38	81/11590	0,234	0,501	0,473	NMT1	1
GO:0046031	ADP metabolic process	1/38	82/11590	0,237	0,503	0,474	GALK1	1
GO:1901890	positive regulation of cell junction assembly	1/38	82/11590	0,237	0,503	0,474	STAU2	1
GO:1902414	protein localization to cell junction	1/38	82/11590	0,237	0,503	0,474	STAU2	1
GO:0000079	regulation of cyclin-dependent protein serine/threonine kinase activity	1/38	83/11590	0,239	0,503	0,474	HEXIM1	1
GO:0030316	osteoclast differentiation	1/38	83/11590	0,239	0,503	0,474	SH3PXD2A	1
GO:0050773	regulation of dendrite development	1/38	83/11590	0,239	0,503	0,474	STAU2	1
GO:0090263	positive regulation of canonical Wnt signaling pathway	1/38	83/11590	0,239	0,503	0,474	GPC5	1
GO:0002831	regulation of response to biotic stimulus	2/38	285/11590	0,240	0,503	0,474	APOE/HEXIM1	2
GO:0007265	Ras protein signal transduction	2/38	285/11590	0,240	0,503	0,474	APOE/ARHGEF12	2
GO:0032784	regulation of DNA-templated transcription elongation	1/38	84/11590	0,242	0,503	0,475	HEXIM1	1
GO:0051341	regulation of oxidoreductase activity	1/38	84/11590	0,242	0,503	0,475	APOE	1

GO:1904029	regulation of cyclin-dependent protein kinase activity	1/38	84/11590	0,242	0,503	0,475	HEXIM1	1
GO:1990748	cellular detoxification	1/38	84/11590	0,242	0,503	0,475	APOE	1
GO:0006165	nucleoside diphosphate phosphorylation	1/38	85/11590	0,244	0,504	0,475	GALK1	1
GO:0043535	regulation of blood vessel endothelial cell migration	1/38	85/11590	0,244	0,504	0,475	APOE	1
GO:0051952	regulation of amine transport	1/38	85/11590	0,244	0,504	0,475	SLC38A1	1
GO:0010639	negative regulation of organelle organization	2/38	289/11590	0,245	0,504	0,475	STN1/H3-3A	2
GO:0042176	regulation of protein catabolic process	2/38	289/11590	0,245	0,504	0,475	UFL1/APOE	2
GO:0006644	phospholipid metabolic process	2/38	290/11590	0,246	0,505	0,476	MTMR9/APOC1	2
GO:0031058	positive regulation of histone modification	1/38	86/11590	0,247	0,505	0,476	WBP2	1
GO:0048259	regulation of receptor-mediated endocytosis	1/38	86/11590	0,247	0,505	0,476	APOC1	1
GO:0007044	cell-substrate junction assembly	1/38	87/11590	0,249	0,506	0,477	STON1	1
GO:0046939	nucleotide phosphorylation	1/38	87/11590	0,249	0,506	0,477	GALK1	1
GO:0007006	mitochondrial membrane organization	1/38	88/11590	0,252	0,506	0,477	NMT1	1
GO:0010769	regulation of cell morphogenesis involved in differentiation	1/38	88/11590	0,252	0,506	0,477	STAU2	1
GO:0014013	regulation of gliogenesis	1/38	88/11590	0,252	0,506	0,477	UFL1	1
GO:1901796	regulation of signal transduction by p53 class mediator	1/38	88/11590	0,252	0,506	0,477	HEXIM1	1
GO:0050727	regulation of inflammatory response	2/38	295/11590	0,252	0,506	0,477	UFL1/APOE	2
GO:0006368	transcription elongation by RNA polymerase II promoter	1/38	89/11590	0,254	0,506	0,477	HEXIM1	1
GO:0008637	apoptotic mitochondrial changes	1/38	89/11590	0,254	0,506	0,477	NMT1	1
GO:0051224	negative regulation of protein transport	1/38	89/11590	0,254	0,506	0,477	APOE	1
GO:0060840	artery development	1/38	89/11590	0,254	0,506	0,477	APOE	1
GO:0042326	negative regulation of phosphorylation	2/38	297/11590	0,254	0,506	0,477	APOE/HEXIM1	2
GO:0033559	unsaturated fatty acid metabolic process	1/38	90/11590	0,257	0,506	0,477	ACOX1	1
GO:0048545	response to steroid hormone	2/38	299/11590	0,257	0,506	0,477	UFL1/WBP2	2

GO:0031346	positive regulation of cell projection organization	2/38	300/11590	0,258	0,506	0,477	STAU2/APOE	2
GO:0009135	purine nucleoside diphosphate metabolic process	1/38	91/11590	0,259	0,506	0,477	GALK1	1
GO:0009179	purine ribonucleoside diphosphate metabolic process	1/38	91/11590	0,259	0,506	0,477	GALK1	1
GO:0019080	viral gene expression	1/38	91/11590	0,259	0,506	0,477	HEXIM1	1
GO:0033865	nucleoside bisphosphate metabolic process	1/38	91/11590	0,259	0,506	0,477	DCAKD	1
GO:0033875	ribonucleoside bisphosphate metabolic process	1/38	91/11590	0,259	0,506	0,477	DCAKD	1
GO:0034032	purine nucleoside bisphosphate metabolic process	1/38	91/11590	0,259	0,506	0,477	DCAKD	1
GO:0097237	cellular response to toxic substance	1/38	91/11590	0,259	0,506	0,477	APOE	1
GO:1905954	positive regulation of lipid localization	1/38	91/11590	0,259	0,506	0,477	APOE	1
GO:0015837	amine transport	1/38	92/11590	0,262	0,508	0,480	SLC38A1	1
GO:0006690	icosanoid metabolic process	1/38	93/11590	0,264	0,508	0,480	ACOX1	1
GO:0007173	epidermal growth factor receptor signaling pathway	1/38	93/11590	0,264	0,508	0,480	EFEMP1	1
GO:0019395	fatty acid oxidation	1/38	93/11590	0,264	0,508	0,480	ACOX1	1
GO:0034728	nucleosome organization	1/38	93/11590	0,264	0,508	0,480	H3-3A	1
GO:0150115	cell-substrate junction organization	1/38	93/11590	0,264	0,508	0,480	STON1	1
GO:1904950	negative regulation of establishment of protein localization	1/38	93/11590	0,264	0,508	0,480	APOE	1
GO:0035967	cellular response to topologically incorrect protein	1/38	94/11590	0,267	0,511	0,482	UFL1	1
GO:0061387	regulation of extent of cell growth	1/38	94/11590	0,267	0,511	0,482	APOE	1
GO:0051702	biological process involved in interaction with symbiont	1/38	95/11590	0,269	0,515	0,485	APOE	1
GO:0002062	chondrocyte differentiation	1/38	96/11590	0,271	0,516	0,487	EFEMP1	1
GO:0043200	response to amino acid	1/38	96/11590	0,271	0,516	0,487	UFL1	1
GO:0006869	lipid transport	2/38	312/11590	0,273	0,516	0,487	APOE/APOC1	2
GO:0019751	polyol metabolic process	1/38	97/11590	0,274	0,516	0,487	GALK1	1

GO:0072655	establishment of protein localization to mitochondrion	1/38	97/11590	0,274	0,516	0,487	NMT1	1
GO:0031331	positive regulation of cellular catabolic process	2/38	313/11590	0,274	0,516	0,487	UFL1/APOE	2
GO:0050767	regulation of neurogenesis	2/38	313/11590	0,274	0,516	0,487	UFL1/STAU2	2
GO:0071900	regulation of protein serine/threonine kinase activity	2/38	313/11590	0,274	0,516	0,487	APOE/HEXIM 1	2
GO:0007338	single fertilization	1/38	98/11590	0,276	0,517	0,488	H3-3A	1
GO:0034440	lipid oxidation	1/38	98/11590	0,276	0,517	0,488	ACOX1	1
GO:0071482	cellular response to light stimulus	1/38	98/11590	0,276	0,517	0,488	NMT1	1
GO:0001654	eye development	2/38	316/11590	0,278	0,519	0,489	STAU2/EFEM P1	2
GO:0007613	memory	1/38	99/11590	0,279	0,519	0,489	APOE	1
GO:0009185	ribonucleoside diphosphate metabolic process	1/38	99/11590	0,279	0,519	0,489	GALK1	1
GO:0006090	pyruvate metabolic process	1/38	100/11590	0,281	0,520	0,491	GALK1	1
GO:0072329	monocarboxylic acid catabolic process	1/38	100/11590	0,281	0,520	0,491	ACOX1	1
GO:0090090	negative regulation of canonical Wnt signaling pathway	1/38	100/11590	0,281	0,520	0,491	APOE	1
GO:0150063	visual system development	2/38	319/11590	0,281	0,520	0,491	STAU2/EFEM P1	2
GO:0001701	in utero embryonic development	2/38	321/11590	0,284	0,524	0,494	SH3PXD2A/N MT1	2
GO:0070585	protein localization to mitochondrion	1/38	102/11590	0,286	0,526	0,496	NMT1	1
GO:0048880	sensory system development	2/38	324/11590	0,287	0,529	0,498	STAU2/EFEM P1	2
GO:0035303	regulation of dephosphorylation	1/38	103/11590	0,288	0,529	0,499	MTMR9	1
GO:0043254	regulation of protein-containing complex assembly	2/38	325/11590	0,289	0,529	0,499	APOE/H3-3A	2
GO:0007286	spermatid development	1/38	104/11590	0,290	0,530	0,500	H3-3A	1
GO:0009581	detection of external stimulus	1/38	104/11590	0,290	0,530	0,500	NMT1	1
GO:0030099	myeloid cell differentiation	2/38	328/11590	0,292	0,531	0,501	UFL1/SH3PXD 2A	2

GO:0098754	detoxification	1/38	105/11590	0,293	0,531	0,501	APOE	1
GO:2000278	regulation of DNA biosynthetic process	1/38	105/11590	0,293	0,531	0,501	STN1	1
GO:0002244	hematopoietic progenitor cell differentiation	1/38	106/11590	0,295	0,532	0,501	UFL1	1
GO:0009582	detection of abiotic stimulus	1/38	106/11590	0,295	0,532	0,501	NMT1	1
GO:0038127	ERBB signaling pathway	1/38	106/11590	0,295	0,532	0,501	EFEMP1	1
GO:0048675	axon extension	1/38	106/11590	0,295	0,532	0,501	APOE	1
GO:0050808	synapse organization	2/38	331/11590	0,296	0,532	0,502	STAU2/APOE	2
GO:0010565	regulation of cellular ketone metabolic process	1/38	107/11590	0,297	0,534	0,503	APOC1	1
GO:0051051	negative regulation of transport	2/38	335/11590	0,301	0,537	0,507	APOE/APOC1	2
GO:0001101	response to acid chemical	1/38	109/11590	0,302	0,537	0,507	UFL1	1
GO:0032434	regulation of proteasomal ubiquitin-dependent protein catabolic process	1/38	109/11590	0,302	0,537	0,507	UFL1	1
GO:0048515	spermatid differentiation	1/38	109/11590	0,302	0,537	0,507	H3-3A	1
GO:0006986	response to unfolded protein	1/38	110/11590	0,304	0,537	0,507	UFL1	1
GO:0009132	nucleoside diphosphate metabolic process	1/38	110/11590	0,304	0,537	0,507	GALK1	1
GO:0030216	keratinocyte differentiation	1/38	110/11590	0,304	0,537	0,507	EVPL	1
GO:0030218	erythrocyte differentiation	1/38	110/11590	0,304	0,537	0,507	UFL1	1
GO:0043534	blood vessel endothelial cell migration	1/38	110/11590	0,304	0,537	0,507	APOE	1
GO:0034329	cell junction assembly	2/38	339/11590	0,306	0,538	0,507	STAU2/STON1	2
GO:0007030	Golgi organization	1/38	111/11590	0,307	0,538	0,507	COG2	1
GO:0030177	positive regulation of Wnt signaling pathway	1/38	111/11590	0,307	0,538	0,507	GPC5	1
GO:0045089	positive regulation of innate immune response	1/38	111/11590	0,307	0,538	0,507	HEXIM1	1
GO:0006275	regulation of DNA replication	1/38	112/11590	0,309	0,540	0,509	STN1	1
GO:0051053	negative regulation of DNA metabolic process	1/38	112/11590	0,309	0,540	0,509	STN1	1
GO:0016055	Wnt signaling pathway	2/38	343/11590	0,311	0,541	0,510	APOE/GPC5	2
GO:0010508	positive regulation of autophagy	1/38	113/11590	0,311	0,541	0,510	UFL1	1
GO:0001558	regulation of cell growth	2/38	344/11590	0,312	0,541	0,510	APOE/H3-3A	2

GO:0198738	cell-cell signaling by wnt	2/38	344/11590	0,312	0,541	0,510	APOE/GPC5	2
GO:0006865	amino acid transport	1/38	115/11590	0,316	0,545	0,514	SLC38A1	1
GO:0006997	nucleus organization	1/38	115/11590	0,316	0,545	0,514	H3-3A	1
GO:0007292	female gamete generation	1/38	115/11590	0,316	0,545	0,514	H3-3A	1
GO:0010876	lipid localization	2/38	348/11590	0,317	0,546	0,515	APOE/APOC1	2
GO:0009150	purine ribonucleotide metabolic process	2/38	349/11590	0,318	0,547	0,516	GALK1/DCAKD	2
GO:0010977	negative regulation of neuron projection development	1/38	117/11590	0,320	0,548	0,517	APOE	1
GO:1903008	organelle disassembly	1/38	117/11590	0,320	0,548	0,517	UFL1	1
GO:0006979	response to oxidative stress	2/38	352/11590	0,322	0,548	0,517	STAU2/APOE	2
GO:0030168	platelet activation	1/38	118/11590	0,323	0,548	0,517	APOE	1
GO:0034101	erythrocyte homeostasis	1/38	118/11590	0,323	0,548	0,517	UFL1	1
GO:1903825	organic acid transmembrane transport	1/38	118/11590	0,323	0,548	0,517	SLC38A1	1
GO:1905039	carboxylic acid transmembrane transport	1/38	118/11590	0,323	0,548	0,517	SLC38A1	1
GO:0046165	alcohol biosynthetic process	1/38	119/11590	0,325	0,548	0,517	APOE	1
GO:0046434	organophosphate catabolic process	1/38	119/11590	0,325	0,548	0,517	APOC1	1
GO:0048813	dendrite morphogenesis	1/38	119/11590	0,325	0,548	0,517	STAU2	1
GO:0061041	regulation of wound healing	1/38	119/11590	0,325	0,548	0,517	APOE	1
GO:0001936	regulation of endothelial cell proliferation	1/38	120/11590	0,327	0,550	0,518	APOE	1
GO:0072594	establishment of protein localization to organelle	2/38	357/11590	0,328	0,550	0,518	WBP2/NMT1	2
GO:0000723	telomere maintenance	1/38	121/11590	0,329	0,550	0,518	STN1	1
GO:0008277	regulation of G protein-coupled receptor signaling pathway	1/38	121/11590	0,329	0,550	0,518	NMT1	1
GO:0030178	negative regulation of Wnt signaling pathway	1/38	121/11590	0,329	0,550	0,518	APOE	1
GO:1903531	negative regulation of secretion by cell	1/38	121/11590	0,329	0,550	0,518	APOE	1
GO:0007283	spermatogenesis	2/38	360/11590	0,331	0,552	0,520	H3-3A/ACOX1	2
GO:0050728	negative regulation of inflammatory response	1/38	122/11590	0,332	0,552	0,520	APOE	1

GO:0062013	positive regulation of small molecule metabolic process	1/38	123/11590	0,334	0,553	0,522	APOE	1
GO:0016032	viral process	2/38	362/11590	0,334	0,553	0,522	APOE/HEXIM1	2
GO:0009566	fertilization	1/38	124/11590	0,336	0,555	0,524	H3-3A	1
GO:0050680	negative regulation of epithelial cell proliferation	1/38	124/11590	0,336	0,555	0,524	APOE	1
GO:0009259	ribonucleotide metabolic process	2/38	365/11590	0,337	0,556	0,524	GALK1/DCAKD	2
GO:0008037	cell recognition	1/38	125/11590	0,338	0,556	0,524	VCAN	1
GO:0010821	regulation of mitochondrion organization	1/38	125/11590	0,338	0,556	0,524	NMT1	1
GO:0006633	fatty acid biosynthetic process	1/38	126/11590	0,340	0,558	0,526	APOC1	1
GO:0035264	multicellular organism growth	1/38	126/11590	0,340	0,558	0,526	H3-3A	1
GO:0006163	purine nucleotide metabolic process	2/38	371/11590	0,345	0,560	0,528	GALK1/DCAKD	2
GO:0010975	regulation of neuron projection development	2/38	371/11590	0,345	0,560	0,528	STAU2/APOE	2
GO:0010970	transport along microtubule	1/38	128/11590	0,345	0,560	0,528	STAU2	1
GO:0035966	response to topologically incorrect protein	1/38	128/11590	0,345	0,560	0,528	UFL1	1
GO:0046488	phosphatidylinositol metabolic process	1/38	128/11590	0,345	0,560	0,528	MTMR9	1
GO:0051960	regulation of nervous system development	2/38	372/11590	0,346	0,560	0,528	UFL1/STAU2	2
GO:0008584	male gonad development	1/38	129/11590	0,347	0,560	0,528	H3-3A	1
GO:0019693	ribose phosphate metabolic process	2/38	373/11590	0,347	0,560	0,528	GALK1/DCAKD	2
GO:0048232	male gamete generation	2/38	373/11590	0,347	0,560	0,528	H3-3A/ACOX1	2
GO:0046546	development of primary male sexual characteristics	1/38	130/11590	0,349	0,562	0,530	H3-3A	1
GO:0042060	wound healing	2/38	375/11590	0,349	0,562	0,530	APOE/EVPL	2
GO:0007416	synapse assembly	1/38	131/11590	0,351	0,562	0,530	STAU2	1
GO:1902904	negative regulation of supramolecular fiber organization	1/38	131/11590	0,351	0,562	0,530	APOE	1
GO:2000058	regulation of ubiquitin-dependent protein catabolic process	1/38	131/11590	0,351	0,562	0,530	UFL1	1

GO:0043524	negative regulation of neuron apoptotic process	1/38	132/11590	0,353	0,563	0,531	APOE	1
GO:0048660	regulation of smooth muscle cell proliferation	1/38	132/11590	0,353	0,563	0,531	APOE	1
GO:0050806	positive regulation of synaptic transmission	1/38	132/11590	0,353	0,563	0,531	APOE	1
GO:0016570	histone modification	2/38	381/11590	0,357	0,566	0,534	UFL1/WBP2	2
GO:0001935	endothelial cell proliferation	1/38	134/11590	0,358	0,566	0,534	APOE	1
GO:0016052	carbohydrate catabolic process	1/38	134/11590	0,358	0,566	0,534	GALK1	1
GO:0030307	positive regulation of cell growth	1/38	135/11590	0,360	0,566	0,534	H3-3A	1
GO:0035150	regulation of tube size	1/38	135/11590	0,360	0,566	0,534	APOE	1
GO:0035296	regulation of tube diameter	1/38	135/11590	0,360	0,566	0,534	APOE	1
GO:0043433	negative regulation of DNA-binding transcription factor activity	1/38	135/11590	0,360	0,566	0,534	UFL1	1
GO:0097746	blood vessel diameter maintenance	1/38	135/11590	0,360	0,566	0,534	APOE	1
GO:0048659	smooth muscle cell proliferation	1/38	136/11590	0,362	0,568	0,536	APOE	1
GO:0010498	proteasomal protein catabolic process	2/38	386/11590	0,363	0,569	0,536	UFL1/APOE	2
GO:0043409	negative regulation of MAPK cascade	1/38	137/11590	0,364	0,570	0,537	APOE	1
GO:0031503	protein-containing complex localization	1/38	138/11590	0,366	0,572	0,540	STAU2	1
GO:0051048	negative regulation of secretion	1/38	139/11590	0,368	0,574	0,541	APOE	1
GO:0090316	positive regulation of intracellular protein transport	1/38	139/11590	0,368	0,574	0,541	NMT1	1
GO:0120031	plasma membrane bounded cell projection assembly	2/38	393/11590	0,371	0,577	0,544	STAU2/STON 1	2
GO:0050792	regulation of viral process	1/38	141/11590	0,372	0,577	0,545	HEXIM1	1
GO:1904064	positive regulation of cation transmembrane transport	1/38	141/11590	0,372	0,577	0,545	SLC38A1	1
GO:0072521	purine-containing compound metabolic process	2/38	395/11590	0,373	0,578	0,545	GALK1/DCAK D	2
GO:0009896	positive regulation of catabolic process	2/38	397/11590	0,376	0,578	0,546	UFL1/APOE	2
GO:0022411	cellular component disassembly	2/38	397/11590	0,376	0,578	0,546	UFL1/STON1	2
GO:0006839	mitochondrial transport	1/38	143/11590	0,377	0,578	0,546	NMT1	1
GO:0031056	regulation of histone modification	1/38	143/11590	0,377	0,578	0,546	WBP2	1

GO:0048469	cell maturation	1/38	143/11590	0,377	0,578	0,546	H3-3A	1
GO:0002833	positive regulation of response to biotic stimulus	1/38	144/11590	0,379	0,580	0,547	HEXIM1	1
GO:1990138	neuron projection extension	1/38	144/11590	0,379	0,580	0,547	APOE	1
GO:0030031	cell projection assembly	2/38	401/11590	0,381	0,581	0,548	STAU2/STON 1	2
GO:0072331	signal transduction by p53 class mediator	1/38	145/11590	0,381	0,581	0,548	HEXIM1	1
GO:0006091	generation of precursor metabolites and energy	2/38	402/11590	0,382	0,581	0,548	GALK1/ACOX 1	2
GO:0007264	small GTPase mediated signal transduction	2/38	402/11590	0,382	0,581	0,548	APOE/ARHGE F12	2
GO:1903034	regulation of response to wounding	1/38	146/11590	0,383	0,582	0,548	APOE	1
GO:1903828	negative regulation of protein localization	1/38	147/11590	0,385	0,584	0,551	APOE	1
GO:0002262	myeloid cell homeostasis	1/38	148/11590	0,387	0,585	0,552	UFL1	1
GO:0046661	male sex differentiation	1/38	148/11590	0,387	0,585	0,552	H3-3A	1
GO:0016049	cell growth	2/38	407/11590	0,388	0,585	0,552	APOE/H3-3A	2
GO:0016573	histone acetylation	1/38	150/11590	0,391	0,588	0,554	WBP2	1
GO:0034249	negative regulation of cellular amide metabolic process	1/38	150/11590	0,391	0,588	0,554	APOE	1
GO:0099111	microtubule-based transport	1/38	150/11590	0,391	0,588	0,554	STAU2	1
GO:0006694	steroid biosynthetic process	1/38	151/11590	0,393	0,589	0,555	APOE	1
GO:0010594	regulation of endothelial cell migration	1/38	151/11590	0,393	0,589	0,555	APOE	1
GO:0051223	regulation of protein transport	2/38	412/11590	0,393	0,589	0,555	APOE/NMT1	2
GO:0099504	synaptic vesicle cycle	1/38	152/11590	0,395	0,589	0,556	STON1	1
GO:1902905	positive regulation of supramolecular fiber organization	1/38	152/11590	0,395	0,589	0,556	APOE	1
GO:0034767	positive regulation of ion transmembrane transport	1/38	153/11590	0,397	0,591	0,558	SLC38A1	1
GO:0065004	protein-DNA complex assembly	1/38	154/11590	0,399	0,593	0,560	H3-3A	1
GO:0018393	internal peptidyl-lysine acetylation	1/38	155/11590	0,401	0,594	0,560	WBP2	1

GO:0031345	negative regulation of cell projection organization	1/38	155/11590	0,401	0,594	0,560	APOE	1
GO:0051099	positive regulation of binding	1/38	155/11590	0,401	0,594	0,560	APOE	1
GO:0006475	internal protein amino acid acetylation	1/38	157/11590	0,405	0,597	0,563	WBP2	1
GO:0071478	cellular response to radiation	1/38	157/11590	0,405	0,597	0,563	NMT1	1
GO:0008361	regulation of cell size	1/38	158/11590	0,407	0,597	0,563	APOE	1
GO:0030705	cytoskeleton-dependent intracellular transport	1/38	158/11590	0,407	0,597	0,563	STAU2	1
GO:0120032	regulation of plasma membrane bounded cell projection assembly	1/38	158/11590	0,407	0,597	0,563	STAU2	1
GO:0006914	autophagy	2/38	424/11590	0,407	0,597	0,563	UFL1/MTMR9	2
GO:0061919	process utilizing autophagic mechanism	2/38	424/11590	0,407	0,597	0,563	UFL1/MTMR9	2
GO:0046578	regulation of Ras protein signal transduction	1/38	159/11590	0,409	0,597	0,563	APOE	1
GO:0060491	regulation of cell projection assembly	1/38	159/11590	0,409	0,597	0,563	STAU2	1
GO:0009913	epidermal cell differentiation	1/38	160/11590	0,411	0,598	0,564	EVPL	1
GO:0031396	regulation of protein ubiquitination	1/38	160/11590	0,411	0,598	0,564	UFL1	1
GO:1901888	regulation of cell junction assembly	1/38	161/11590	0,413	0,600	0,566	STAU2	1
GO:0071897	DNA biosynthetic process	1/38	162/11590	0,415	0,602	0,568	STN1	1
GO:0018394	peptidyl-lysine acetylation	1/38	163/11590	0,417	0,603	0,569	WBP2	1
GO:0070201	regulation of establishment of protein localization	2/38	433/11590	0,418	0,603	0,569	APOE/NMT1	2
GO:0043405	regulation of MAP kinase activity	1/38	164/11590	0,419	0,603	0,569	APOE	1
GO:0045732	positive regulation of protein catabolic process	1/38	164/11590	0,419	0,603	0,569	APOE	1
GO:0070085	glycosylation	1/38	164/11590	0,419	0,603	0,569	COG2	1
GO:0060284	regulation of cell development	2/38	434/11590	0,419	0,603	0,569	UFL1/STAU2	2
GO:0098739	import across plasma membrane	1/38	165/11590	0,421	0,603	0,569	SLC38A1	1
GO:1901605	alpha-amino acid metabolic process	1/38	165/11590	0,421	0,603	0,569	SLC38A1	1
GO:0007601	visual perception	1/38	166/11590	0,423	0,605	0,571	EFEMP1	1
GO:0006403	RNA localization	1/38	167/11590	0,424	0,606	0,572	STAU2	1
GO:0072330	monocarboxylic acid biosynthetic process	1/38	167/11590	0,424	0,606	0,572	APOC1	1

GO:0050953	sensory perception of light stimulus	1/38	168/11590	0,426	0,608	0,574	EFEMP1	1
GO:0007626	locomotory behavior	1/38	169/11590	0,428	0,609	0,574	APOE	1
GO:0099003	vesicle-mediated transport in synapse	1/38	169/11590	0,428	0,609	0,574	STON1	1
GO:1905114	cell surface receptor signaling pathway involved in cell-cell signaling	2/38	443/11590	0,429	0,609	0,575	APOE/GPC5	2
GO:0000302	response to reactive oxygen species	1/38	170/11590	0,430	0,609	0,575	APOE	1
GO:0043393	regulation of protein binding	1/38	170/11590	0,430	0,609	0,575	APOE	1
GO:0051216	cartilage development	1/38	172/11590	0,434	0,614	0,579	EFEMP1	1
GO:0046034	ATP metabolic process	1/38	173/11590	0,436	0,615	0,580	GALK1	1
GO:0001501	skeletal system development	2/38	450/11590	0,437	0,616	0,581	VCAN/EFEMP1	2
GO:0032388	positive regulation of intracellular transport	1/38	174/11590	0,438	0,616	0,581	NMT1	1
GO:0009152	purine ribonucleotide biosynthetic process	1/38	175/11590	0,440	0,618	0,583	DCAKD	1
GO:0007423	sensory organ development	2/38	453/11590	0,441	0,619	0,584	STAU2/EFEMP1	2
GO:0051276	chromosome organization	2/38	455/11590	0,443	0,621	0,585	STN1/H3-3A	2
GO:0050821	protein stabilization	1/38	177/11590	0,443	0,621	0,585	MTMR9	1
GO:0050866	negative regulation of cell activation	1/38	178/11590	0,445	0,621	0,586	APOE	1
GO:1901654	response to ketone	1/38	178/11590	0,445	0,621	0,586	WBP2	1
GO:0006836	neurotransmitter transport	1/38	179/11590	0,447	0,622	0,587	SLC38A1	1
GO:0070374	positive regulation of ERK1 and ERK2 cascade	1/38	179/11590	0,447	0,622	0,587	APOE	1
GO:0009117	nucleotide metabolic process	2/38	461/11590	0,450	0,624	0,588	GALK1/DCAKD	2
GO:0048667	cell morphogenesis involved in neuron differentiation	2/38	461/11590	0,450	0,624	0,588	STAU2/APOE	2
GO:0002573	myeloid leukocyte differentiation	1/38	181/11590	0,451	0,624	0,588	SH3PXD2A	1
GO:1901215	negative regulation of neuron death	1/38	181/11590	0,451	0,624	0,588	APOE	1
GO:0006325	chromatin organization	2/38	466/11590	0,455	0,629	0,594	WBP2/H3-3A	2
GO:0006753	nucleoside phosphate metabolic process	2/38	467/11590	0,456	0,629	0,594	GALK1/DCAKD	2

GO:0001505	regulation of neurotransmitter levels	1/38	185/11590	0,458	0,629	0,594	SLC38A1	1
GO:0006473	protein acetylation	1/38	185/11590	0,458	0,629	0,594	WBP2	1
GO:0071824	protein-DNA complex subunit organization	1/38	185/11590	0,458	0,629	0,594	H3-3A	1
GO:0009260	ribonucleotide biosynthetic process	1/38	186/11590	0,460	0,630	0,594	DCAKD	1
GO:0051651	maintenance of location in cell	1/38	186/11590	0,460	0,630	0,594	APOE	1
GO:0033002	muscle cell proliferation	1/38	187/11590	0,462	0,632	0,596	APOE	1
GO:0006814	sodium ion transport	1/38	189/11590	0,465	0,635	0,599	SLC38A1	1
GO:0043523	regulation of neuron apoptotic process	1/38	189/11590	0,465	0,635	0,599	APOE	1
GO:0048588	developmental cell growth	1/38	191/11590	0,469	0,639	0,602	APOE	1
GO:0009205	purine ribonucleoside triphosphate metabolic process	1/38	192/11590	0,470	0,639	0,602	GALK1	1
GO:0006164	purine nucleotide biosynthetic process	1/38	193/11590	0,472	0,639	0,602	DCAKD	1
GO:0046390	ribose phosphate biosynthetic process	1/38	193/11590	0,472	0,639	0,602	DCAKD	1
GO:0046395	carboxylic acid catabolic process	1/38	193/11590	0,472	0,639	0,602	ACOX1	1
GO:0009636	response to toxic substance	1/38	194/11590	0,474	0,639	0,602	APOE	1
GO:0019318	hexose metabolic process	1/38	194/11590	0,474	0,639	0,602	GALK1	1
GO:0033157	regulation of intracellular protein transport	1/38	194/11590	0,474	0,639	0,602	NMT1	1
GO:0090150	establishment of protein localization to membrane	1/38	195/11590	0,476	0,639	0,602	NMT1	1
GO:0009144	purine nucleoside triphosphate metabolic process	1/38	196/11590	0,478	0,639	0,602	GALK1	1
GO:0060560	developmental growth involved in morphogenesis	1/38	196/11590	0,478	0,639	0,602	APOE	1
GO:0008406	gonad development	1/38	197/11590	0,479	0,639	0,602	H3-3A	1
GO:0009199	ribonucleoside triphosphate metabolic process	1/38	197/11590	0,479	0,639	0,602	GALK1	1
GO:0016054	organic acid catabolic process	1/38	197/11590	0,479	0,639	0,602	ACOX1	1
GO:0071695	anatomical structure maturation	1/38	197/11590	0,479	0,639	0,602	H3-3A	1
GO:0071407	cellular response to organic cyclic compound	2/38	489/11590	0,481	0,639	0,602	UFL1/WBP2	2
GO:0034764	positive regulation of transmembrane transport	1/38	198/11590	0,481	0,639	0,602	SLC38A1	1

GO:0043122	regulation of I-kappaB kinase/NF-kappaB signaling	1/38	198/11590	0,481	0,639	0,602	UFL1	1
GO:0043542	endothelial cell migration	1/38	198/11590	0,481	0,639	0,602	APOE	1
GO:1903320	regulation of protein modification by small protein conjugation or removal	1/38	198/11590	0,481	0,639	0,602	UFL1	1
GO:0031348	negative regulation of defense response	1/38	199/11590	0,483	0,639	0,603	APOE	1
GO:0072522	purine-containing compound biosynthetic process	1/38	199/11590	0,483	0,639	0,603	DCAKD	1
GO:0007596	blood coagulation	1/38	200/11590	0,484	0,641	0,604	APOE	1
GO:0009611	response to wounding	2/38	493/11590	0,485	0,641	0,604	APOE/EVPL	2
GO:0045137	development of primary sexual characteristics	1/38	201/11590	0,486	0,641	0,605	H3-3A	1
GO:0098656	anion transmembrane transport	1/38	202/11590	0,488	0,643	0,606	SLC38A1	1
GO:0034976	response to endoplasmic reticulum stress	1/38	203/11590	0,490	0,643	0,607	UFL1	1
GO:0050817	coagulation	1/38	203/11590	0,490	0,643	0,607	APOE	1
GO:0048863	stem cell differentiation	1/38	204/11590	0,491	0,645	0,608	UFL1	1
GO:0007599	hemostasis	1/38	205/11590	0,493	0,646	0,609	APOE	1
GO:0005996	monosaccharide metabolic process	1/38	206/11590	0,495	0,646	0,610	GALK1	1
GO:0007160	cell-matrix adhesion	1/38	206/11590	0,495	0,646	0,610	STON1	1
GO:0010632	regulation of epithelial cell migration	1/38	207/11590	0,496	0,648	0,611	APOE	1
GO:0009141	nucleoside triphosphate metabolic process	1/38	209/11590	0,500	0,651	0,614	GALK1	1
GO:0050708	regulation of protein secretion	1/38	210/11590	0,501	0,651	0,614	APOE	1
GO:0098657	import into cell	1/38	210/11590	0,501	0,651	0,614	SLC38A1	1
GO:1901617	organic hydroxy compound biosynthetic process	1/38	210/11590	0,501	0,651	0,614	APOE	1
GO:0045927	positive regulation of growth	1/38	214/11590	0,508	0,659	0,621	H3-3A	1
GO:0043588	skin development	1/38	217/11590	0,513	0,663	0,625	EVPL	1
GO:0051606	detection of stimulus	1/38	217/11590	0,513	0,663	0,625	NMT1	1
GO:0046942	carboxylic acid transport	1/38	220/11590	0,518	0,669	0,631	SLC38A1	1
GO:0051402	neuron apoptotic process	1/38	223/11590	0,523	0,674	0,636	APOE	1
GO:0007249	I-kappaB kinase/NF-kappaB signaling	1/38	224/11590	0,524	0,675	0,637	UFL1	1

GO:0048562	embryonic organ morphogenesis	1/38	228/11590	0,531	0,682	0,644	EFEMP1	1
GO:0048193	Golgi vesicle transport	1/38	229/11590	0,532	0,683	0,645	COG2	1
GO:0006260	DNA replication	1/38	231/11590	0,535	0,685	0,646	STN1	1
GO:0006520	cellular amino acid metabolic process	1/38	231/11590	0,535	0,685	0,646	SLC38A1	1
GO:0034599	cellular response to oxidative stress	1/38	231/11590	0,535	0,685	0,646	STAU2	1
GO:0007611	learning or memory	1/38	233/11590	0,538	0,686	0,647	APOE	1
GO:0009165	nucleotide biosynthetic process	1/38	233/11590	0,538	0,686	0,647	DCAKD	1
GO:0061448	connective tissue development	1/38	233/11590	0,538	0,686	0,647	EFEMP1	1
GO:1901293	nucleoside phosphate biosynthetic process	1/38	234/11590	0,540	0,687	0,648	DCAKD	1
GO:0006874	cellular calcium ion homeostasis	1/38	235/11590	0,541	0,688	0,649	APOE	1
GO:0031349	positive regulation of defense response	1/38	237/11590	0,545	0,691	0,652	HEXIM1	1
GO:0016236	macroautophagy	1/38	238/11590	0,546	0,692	0,653	UFL1	1
GO:0007548	sex differentiation	1/38	239/11590	0,548	0,692	0,653	H3-3A	1
GO:0021700	developmental maturation	1/38	239/11590	0,548	0,692	0,653	H3-3A	1
GO:0043270	positive regulation of ion transport	1/38	243/11590	0,554	0,698	0,658	SLC38A1	1
GO:0070372	regulation of ERK1 and ERK2 cascade	1/38	243/11590	0,554	0,698	0,658	APOE	1
GO:0044262	cellular carbohydrate metabolic process	1/38	244/11590	0,555	0,699	0,659	GALK1	1
GO:0048872	homeostasis of number of cells	1/38	245/11590	0,557	0,700	0,660	UFL1	1
GO:0030336	negative regulation of cell migration	1/38	247/11590	0,560	0,703	0,663	APOE	1
GO:0022604	regulation of cell morphogenesis	1/38	252/11590	0,567	0,710	0,670	STAU2	1
GO:0051054	positive regulation of DNA metabolic process	1/38	252/11590	0,567	0,710	0,670	STN1	1
GO:0055074	calcium ion homeostasis	1/38	256/11590	0,573	0,717	0,676	APOE	1
GO:0007018	microtubule-based movement	1/38	257/11590	0,574	0,717	0,676	STAU2	1
GO:0044403	biological process involved in symbiotic interaction	1/38	257/11590	0,574	0,717	0,676	APOE	1
GO:0009416	response to light stimulus	1/38	259/11590	0,577	0,717	0,677	NMT1	1
GO:0051222	positive regulation of protein transport	1/38	259/11590	0,577	0,717	0,677	NMT1	1
GO:2000146	negative regulation of cell motility	1/38	259/11590	0,577	0,717	0,677	APOE	1
GO:0048608	reproductive structure development	1/38	260/11590	0,578	0,718	0,677	H3-3A	1

GO:0050890	cognition	1/38	262/11590	0,581	0,719	0,678	APOE	1
GO:0061458	reproductive system development	1/38	262/11590	0,581	0,719	0,678	H3-3A	1
GO:0072503	cellular divalent inorganic cation homeostasis	1/38	262/11590	0,581	0,719	0,678	APOE	1
GO:0008544	epidermis development	1/38	263/11590	0,583	0,720	0,679	EVPL	1
GO:0031647	regulation of protein stability	1/38	264/11590	0,584	0,720	0,679	MTMR9	1
GO:0070371	ERK1 and ERK2 cascade	1/38	264/11590	0,584	0,720	0,679	APOE	1
GO:0042063	gliogenesis	1/38	266/11590	0,587	0,722	0,681	UFL1	1
GO:0016311	dephosphorylation	1/38	269/11590	0,591	0,726	0,684	MTMR9	1
GO:0019932	second-messenger-mediated signaling	1/38	269/11590	0,591	0,726	0,684	APOE	1
GO:0048638	regulation of developmental growth	1/38	271/11590	0,594	0,727	0,685	APOE	1
GO:0010631	epithelial cell migration	1/38	272/11590	0,595	0,727	0,685	APOE	1
GO:0019058	viral life cycle	1/38	272/11590	0,595	0,727	0,685	APOE	1
GO:1904951	positive regulation of establishment of protein localization	1/38	272/11590	0,595	0,727	0,685	NMT1	1
GO:0002253	activation of immune response	1/38	273/11590	0,596	0,727	0,685	HEXIM1	1
GO:0051235	maintenance of location	1/38	274/11590	0,598	0,727	0,685	APOE	1
GO:0051346	negative regulation of hydrolase activity	1/38	274/11590	0,598	0,727	0,685	APOC1	1
GO:0090132	epithelium migration	1/38	274/11590	0,598	0,727	0,685	APOE	1
GO:0015711	organic anion transport	1/38	276/11590	0,600	0,729	0,688	SLC38A1	1
GO:1901214	regulation of neuron death	1/38	278/11590	0,603	0,731	0,690	APOE	1
GO:0032386	regulation of intracellular transport	1/38	279/11590	0,604	0,731	0,690	NMT1	1
GO:0090130	tissue migration	1/38	279/11590	0,604	0,731	0,690	APOE	1
GO:0062197	cellular response to chemical stress	1/38	282/11590	0,608	0,735	0,693	STAU2	1
GO:0009306	protein secretion	1/38	283/11590	0,610	0,735	0,693	APOE	1
GO:0043010	camera-type eye development	1/38	283/11590	0,610	0,735	0,693	EFEMP1	1
GO:0035592	establishment of protein localization to extracellular region	1/38	284/11590	0,611	0,736	0,694	APOE	1
GO:0071214	cellular response to abiotic stimulus	1/38	287/11590	0,615	0,739	0,696	NMT1	1
GO:0104004	cellular response to environmental stimulus	1/38	287/11590	0,615	0,739	0,696	NMT1	1

GO:0040013	negative regulation of locomotion	1/38	288/11590	0,616	0,739	0,697	APOE	1
GO:0071692	protein localization to extracellular region	1/38	289/11590	0,618	0,740	0,698	APOE	1
GO:0072507	divalent inorganic cation homeostasis	1/38	290/11590	0,619	0,740	0,698	APOE	1
GO:0032956	regulation of actin cytoskeleton organization	1/38	291/11590	0,620	0,741	0,699	STAU2	1
GO:1904062	regulation of cation transmembrane transport	1/38	293/11590	0,623	0,743	0,701	SLC38A1	1
GO:0032535	regulation of cellular component size	1/38	297/11590	0,628	0,748	0,706	APOE	1
GO:0045862	positive regulation of proteolysis	1/38	303/11590	0,635	0,756	0,713	APOE	1
GO:0042692	muscle cell differentiation	1/38	307/11590	0,640	0,760	0,717	H3-3A	1
GO:1902903	regulation of supramolecular fiber organization	1/38	307/11590	0,640	0,760	0,717	APOE	1
GO:0050678	regulation of epithelial cell proliferation	1/38	309/11590	0,642	0,762	0,719	APOE	1
GO:0045786	negative regulation of cell cycle	1/38	316/11590	0,651	0,771	0,727	HEXIM1	1
GO:0051098	regulation of binding	1/38	317/11590	0,652	0,771	0,727	APOE	1
GO:0070997	neuron death	1/38	317/11590	0,652	0,771	0,727	APOE	1
GO:0031589	cell-substrate adhesion	1/38	318/11590	0,653	0,771	0,727	STON1	1
GO:0018108	peptidyl-tyrosine phosphorylation	1/38	325/11590	0,661	0,780	0,735	EFEMP1	1
GO:0018212	peptidyl-tyrosine modification	1/38	327/11590	0,664	0,781	0,736	EFEMP1	1
GO:0032970	regulation of actin filament-based process	1/38	327/11590	0,664	0,781	0,736	STAU2	1
GO:0018205	peptidyl-lysine modification	1/38	332/11590	0,669	0,786	0,741	WBP2	1
GO:0006875	cellular metal ion homeostasis	1/38	333/11590	0,670	0,786	0,741	APOE	1
GO:0032102	negative regulation of response to external stimulus	1/38	333/11590	0,670	0,786	0,741	APOE	1
GO:0043161	proteasome-mediated ubiquitin-dependent protein catabolic process	1/38	335/11590	0,673	0,787	0,743	UFL1	1
GO:0050878	regulation of body fluid levels	1/38	341/11590	0,679	0,794	0,749	APOE	1
GO:0051090	regulation of DNA-binding transcription factor activity	1/38	357/11590	0,696	0,813	0,767	UFL1	1
GO:0034248	regulation of cellular amide metabolic process	1/38	358/11590	0,697	0,813	0,767	APOE	1
GO:0048568	embryonic organ development	1/38	361/11590	0,700	0,816	0,769	EFEMP1	1
GO:0007409	axonogenesis	1/38	365/11590	0,704	0,820	0,773	APOE	1

GO:0050673	epithelial cell proliferation	1/38	369/11590	0,708	0,823	0,776	APOE	1
GO:0009314	response to radiation	1/38	371/11590	0,710	0,825	0,778	NMT1	1
GO:0032103	positive regulation of response to external stimulus	1/38	374/11590	0,713	0,827	0,780	HEXIM1	1
GO:0001667	ameboidal-type cell migration	1/38	379/11590	0,718	0,832	0,784	APOE	1
GO:0034765	regulation of ion transmembrane transport	1/38	382/11590	0,721	0,834	0,786	SLC38A1	1
GO:0006820	anion transport	1/38	386/11590	0,725	0,837	0,790	SLC38A1	1
GO:1903829	positive regulation of protein localization	1/38	391/11590	0,729	0,842	0,794	NMT1	1
GO:0030003	cellular cation homeostasis	1/38	392/11590	0,730	0,842	0,794	APOE	1
GO:0007005	mitochondrion organization	1/38	394/11590	0,732	0,843	0,795	NMT1	1
GO:1901361	organic cyclic compound catabolic process	1/38	397/11590	0,735	0,845	0,797	APOE	1
GO:0031667	response to nutrient levels	1/38	403/11590	0,740	0,850	0,802	APOE	1
GO:0061564	axon development	1/38	404/11590	0,741	0,850	0,802	APOE	1
GO:0006873	cellular ion homeostasis	1/38	406/11590	0,743	0,851	0,803	APOE	1
GO:0044089	positive regulation of cellular component biogenesis	1/38	407/11590	0,744	0,851	0,803	STAU2	1
GO:0043410	positive regulation of MAPK cascade	1/38	413/11590	0,749	0,856	0,807	APOE	1
GO:0010256	endomembrane system organization	1/38	414/11590	0,750	0,856	0,807	COG2	1
GO:1902532	negative regulation of intracellular signal transduction	1/38	415/11590	0,750	0,856	0,807	APOE	1
GO:0055065	metal ion homeostasis	1/38	420/11590	0,755	0,860	0,811	APOE	1
GO:0010638	positive regulation of organelle organization	1/38	423/11590	0,757	0,862	0,813	NMT1	1
GO:0090066	regulation of anatomical structure size	1/38	425/11590	0,759	0,863	0,814	APOE	1
GO:0009991	response to extracellular stimulus	1/38	429/11590	0,762	0,865	0,816	APOE	1
GO:0051052	regulation of DNA metabolic process	1/38	430/11590	0,763	0,865	0,816	STN1	1
GO:0051493	regulation of cytoskeleton organization	1/38	438/11590	0,769	0,871	0,822	STAU2	1
GO:0050778	positive regulation of immune response	1/38	441/11590	0,772	0,873	0,823	HEXIM1	1
GO:0006281	DNA repair	1/38	451/11590	0,779	0,881	0,831	UFL1	1
GO:0008015	blood circulation	1/38	452/11590	0,780	0,881	0,831	APOE	1

GO:0034762	regulation of transmembrane transport	1/38	454/11590	0,781	0,881	0,831	SLC38A1	1
GO:0051345	positive regulation of hydrolase activity	1/38	461/11590	0,787	0,886	0,836	MTMR9	1
GO:0007600	sensory perception	1/38	464/11590	0,789	0,888	0,837	EFEMP1	1
GO:0090407	organophosphate biosynthetic process	1/38	474/11590	0,796	0,894	0,843	DCAKD	1
GO:0006511	ubiquitin-dependent protein catabolic process	1/38	475/11590	0,797	0,894	0,843	UFL1	1
GO:0044057	regulation of system process	1/38	479/11590	0,799	0,894	0,843	APOE	1
GO:0005975	carbohydrate metabolic process	1/38	480/11590	0,800	0,894	0,843	GALK1	1
GO:0051347	positive regulation of transferase activity	1/38	480/11590	0,800	0,894	0,843	APOE	1
GO:1903530	regulation of secretion by cell	1/38	480/11590	0,800	0,894	0,843	APOE	1
GO:0007507	heart development	1/38	481/11590	0,801	0,894	0,843	HEXIM1	1
GO:0055080	cation homeostasis	1/38	483/11590	0,802	0,894	0,843	APOE	1
GO:0019941	modification-dependent protein catabolic process	1/38	485/11590	0,803	0,895	0,844	UFL1	1
GO:0048598	embryonic morphogenesis	1/38	490/11590	0,807	0,897	0,846	EFEMP1	1
GO:1901137	carbohydrate derivative biosynthetic process	1/38	491/11590	0,807	0,897	0,846	DCAKD	1
GO:0043632	modification-dependent macromolecule catabolic process	1/38	492/11590	0,808	0,897	0,846	UFL1	1
GO:0098771	inorganic ion homeostasis	1/38	496/11590	0,811	0,899	0,848	APOE	1
GO:0001525	angiogenesis	0/38	464/11590	1,000	1,000	0,943		0
GO:0001764	neuron migration	0/38	142/11590	1,000	1,000	0,943		0
GO:0001890	placenta development	0/38	132/11590	1,000	1,000	0,943		0
GO:0001892	embryonic placenta development	0/38	78/11590	1,000	1,000	0,943		0
GO:0001906	cell killing	0/38	150/11590	1,000	1,000	0,943		0
GO:0001909	leukocyte mediated cytotoxicity	0/38	112/11590	1,000	1,000	0,943		0
GO:0002181	cytoplasmic translation	0/38	125/11590	1,000	1,000	0,943		0
GO:0002228	natural killer cell mediated immunity	0/38	60/11590	1,000	1,000	0,943		0
GO:0002250	adaptive immune response	0/38	389/11590	1,000	1,000	0,943		0
GO:0002263	cell activation involved in immune response	0/38	246/11590	1,000	1,000	0,943		0
GO:0002274	myeloid leukocyte activation	0/38	198/11590	1,000	1,000	0,943		0

GO:0002275	myeloid cell activation involved in immune response	0/38	86/11590	1,000	1,000	0,943	0
GO:0002279	mast cell activation involved in immune response	0/38	49/11590	1,000	1,000	0,943	0
GO:0002285	lymphocyte activation involved in immune response	0/38	165/11590	1,000	1,000	0,943	0
GO:0002323	natural killer cell activation involved in immune response	0/38	24/11590	1,000	1,000	0,943	0
GO:0002366	leukocyte activation involved in immune response	0/38	242/11590	1,000	1,000	0,943	0
GO:0002443	leukocyte mediated immunity	0/38	336/11590	1,000	1,000	0,943	0
GO:0002444	myeloid leukocyte mediated immunity	0/38	94/11590	1,000	1,000	0,943	0
GO:0002448	mast cell mediated immunity	0/38	49/11590	1,000	1,000	0,943	0
GO:0002449	lymphocyte mediated immunity	0/38	253/11590	1,000	1,000	0,943	0
GO:0002460	adaptive immune response based on somatic recombination of immune receptors built from immunoglobulin superfamily domains	0/38	261/11590	1,000	1,000	0,943	0
GO:0002467	germinal center formation	0/38	11/11590	1,000	1,000	0,943	0
GO:0002544	chronic inflammatory response	0/38	18/11590	1,000	1,000	0,943	0
GO:0002694	regulation of leukocyte activation	0/38	492/11590	1,000	1,000	0,943	0
GO:0002697	regulation of immune effector process	0/38	319/11590	1,000	1,000	0,943	0
GO:0002699	positive regulation of immune effector process	0/38	224/11590	1,000	1,000	0,943	0
GO:0002703	regulation of leukocyte mediated immunity	0/38	203/11590	1,000	1,000	0,943	0
GO:0002886	regulation of myeloid leukocyte mediated immunity	0/38	56/11590	1,000	1,000	0,943	0
GO:0006352	DNA-templated transcription initiation	0/38	115/11590	1,000	1,000	0,943	0
GO:0006367	transcription initiation at RNA polymerase II promoter	0/38	80/11590	1,000	1,000	0,943	0
GO:0006417	regulation of translation	0/38	307/11590	1,000	1,000	0,943	0
GO:0006887	exocytosis	0/38	288/11590	1,000	1,000	0,943	0
GO:0006909	phagocytosis	0/38	211/11590	1,000	1,000	0,943	0
GO:0007043	cell-cell junction assembly	0/38	124/11590	1,000	1,000	0,943	0

GO:0007163	establishment or maintenance of cell polarity	0/38	196/11590	1,000	1,000	0,943	0
GO:0007339	binding of sperm to zona pellucida	0/38	26/11590	1,000	1,000	0,943	0
GO:0009116	nucleoside metabolic process	0/38	44/11590	1,000	1,000	0,943	0
GO:0009119	ribonucleoside metabolic process	0/38	28/11590	1,000	1,000	0,943	0
GO:0009163	nucleoside biosynthetic process	0/38	11/11590	1,000	1,000	0,943	0
GO:0009615	response to virus	0/38	329/11590	1,000	1,000	0,943	0
GO:0009988	cell-cell recognition	0/38	47/11590	1,000	1,000	0,943	0
GO:0010608	post-transcriptional regulation of gene expression	0/38	393/11590	1,000	1,000	0,943	0
GO:0010810	regulation of cell-substrate adhesion	0/38	194/11590	1,000	1,000	0,943	0
GO:0010811	positive regulation of cell-substrate adhesion	0/38	113/11590	1,000	1,000	0,943	0
GO:0017148	negative regulation of translation	0/38	131/11590	1,000	1,000	0,943	0
GO:0017157	regulation of exocytosis	0/38	177/11590	1,000	1,000	0,943	0
GO:0030010	establishment of cell polarity	0/38	125/11590	1,000	1,000	0,943	0
GO:0030101	natural killer cell activation	0/38	78/11590	1,000	1,000	0,943	0
GO:0032418	lysosome localization	0/38	70/11590	1,000	1,000	0,943	0
GO:0032543	mitochondrial translation	0/38	88/11590	1,000	1,000	0,943	0
GO:0033003	regulation of mast cell activation	0/38	41/11590	1,000	1,000	0,943	0
GO:0033006	regulation of mast cell activation involved in immune response	0/38	32/11590	1,000	1,000	0,943	0
GO:0034404	nucleobase-containing small molecule biosynthetic process	0/38	11/11590	1,000	1,000	0,943	0
GO:0034446	substrate adhesion-dependent cell spreading	0/38	98/11590	1,000	1,000	0,943	0
GO:0035036	sperm-egg recognition	0/38	30/11590	1,000	1,000	0,943	0
GO:0042267	natural killer cell mediated cytotoxicity	0/38	59/11590	1,000	1,000	0,943	0
GO:0042278	purine nucleoside metabolic process	0/38	20/11590	1,000	1,000	0,943	0
GO:0043094	cellular metabolic compound salvage	0/38	22/11590	1,000	1,000	0,943	0
GO:0043101	purine-containing compound salvage	0/38	12/11590	1,000	1,000	0,943	0
GO:0043297	apical junction assembly	0/38	58/11590	1,000	1,000	0,943	0

GO:0043299	leukocyte degranulation	0/38	69/11590	1,000	1,000	0,943	0
GO:0043300	regulation of leukocyte degranulation	0/38	45/11590	1,000	1,000	0,943	0
GO:0043303	mast cell degranulation	0/38	47/11590	1,000	1,000	0,943	0
GO:0043304	regulation of mast cell degranulation	0/38	30/11590	1,000	1,000	0,943	0
GO:0044782	cilium organization	0/38	236/11590	1,000	1,000	0,943	0
GO:0045055	regulated exocytosis	0/38	195/11590	1,000	1,000	0,943	0
GO:0045216	cell-cell junction organization	0/38	170/11590	1,000	1,000	0,943	0
GO:0045576	mast cell activation	0/38	60/11590	1,000	1,000	0,943	0
GO:0045785	positive regulation of cell adhesion	0/38	420/11590	1,000	1,000	0,943	0
GO:0045921	positive regulation of exocytosis	0/38	76/11590	1,000	1,000	0,943	0
GO:0046128	purine ribonucleoside metabolic process	0/38	17/11590	1,000	1,000	0,943	0
GO:0048015	phosphatidylinositol-mediated signaling	0/38	151/11590	1,000	1,000	0,943	0
GO:0048017	inositol lipid-mediated signaling	0/38	154/11590	1,000	1,000	0,943	0
GO:0051047	positive regulation of secretion	0/38	271/11590	1,000	1,000	0,943	0
GO:0051607	defense response to virus	0/38	239/11590	1,000	1,000	0,943	0
GO:0051640	organelle localization	0/38	450/11590	1,000	1,000	0,943	0
GO:0051656	establishment of organelle localization	0/38	347/11590	1,000	1,000	0,943	0
GO:0051972	regulation of telomerase activity	0/38	38/11590	1,000	1,000	0,943	0
GO:0060271	cilium assembly	0/38	225/11590	1,000	1,000	0,943	0
GO:0060674	placenta blood vessel development	0/38	28/11590	1,000	1,000	0,943	0
GO:0060711	labyrinthine layer development	0/38	43/11590	1,000	1,000	0,943	0
GO:0060716	labyrinthine layer blood vessel development	0/38	18/11590	1,000	1,000	0,943	0
GO:0090162	establishment of epithelial cell polarity	0/38	28/11590	1,000	1,000	0,943	0
GO:0140053	mitochondrial gene expression	0/38	113/11590	1,000	1,000	0,943	0
GO:0140546	defense response to symbiont	0/38	240/11590	1,000	1,000	0,943	0
GO:1900024	regulation of substrate adhesion-dependent cell spreading	0/38	56/11590	1,000	1,000	0,943	0
GO:1900026	positive regulation of substrate adhesion-dependent cell spreading	0/38	41/11590	1,000	1,000	0,943	0

GO:1901657	glycosyl compound metabolic process	0/38	62/11590	1,000	1,000	0,943	0
GO:1901659	glycosyl compound biosynthetic process	0/38	12/11590	1,000	1,000	0,943	0
GO:1903305	regulation of regulated secretory pathway	0/38	121/11590	1,000	1,000	0,943	0
GO:1903307	positive regulation of regulated secretory pathway	0/38	44/11590	1,000	1,000	0,943	0
GO:1903532	positive regulation of secretion by cell	0/38	248/11590	1,000	1,000	0,943	0
GO:1990849	vacuolar localization	0/38	70/11590	1,000	1,000	0,943	0
GO:2000112	regulation of cellular macromolecule biosynthetic process	0/38	371/11590	1,000	1,000	0,943	0
GO:2000113	negative regulation of cellular macromolecule biosynthetic process	0/38	147/11590	1,000	1,000	0,943	0
GO:2000765	regulation of cytoplasmic translation	0/38	20/11590	1,000	1,000	0,943	0

Supplementary Table 6. Enrichment analysis results for the SNPs-related genes of the PRS-WMH before the clumping.
Legend: GeneRatio: ratio of input genes that are annotated in a term (GeneRatio=k/n, where k: overlap of the SNPs-related gene IDs with the specific gene set related to a biological pathway; n: size of the overlap of the SNPs-related gene IDs with all the members of the collection of gene sets); BgRatio: ratio of all genes that are annotated in a term (BgRatio=count/setSize, where count: number of genes that belong to a given gene-set; setSize: total number of genes in the collection of gene sets); P-value: probability of seeing at least X number of genes out of the total n SNPs-related gene IDs in the list annotated to a particular GO term, given the proportion of genes in the whole genome that are annotated to that GO term; FDR: p-value after false discovery rate correction; Q-value: proportion of false positives incurred when the test is significant (FDR correction among the significant results); Counts: number of genes annotated to the GO term.

Model	ρ	P-value	Low CI	High CI	adj P-value
SBP| periv_basal.ganglia ~ age_scan+Sex	0.092	**0.013**	0.019	0.164	**0.03**
SBP| deep_basal.ganglia~ age_scan+Sex	0.079	**0.034**	0.006	0.151	0.064
SBP| juxtacort_basal.ganglia~ age_scan+Sex	0.072	**0.047**	0.001	0.143	0.0705
SBP| periv_frontal~ age_scan+Sex	0.105	**0.004**	0.034	0.175	**0.03**
SBP| periv_parietal~ age_scan+Sex	0.093	**0.012**	0.02	0.164	**0.03**
SBP| periv_occipital~ age_scan+Sex	0.023	0.534	-0.049	0.095	0.613
SBP| periv_temporal~ age_scan+Sex	0.076	**0.042**	0.003	0.148	0.07
SBP| deep_frontal~ age_scan+Sex	0.097	**0.008**	0.026	0.167	**0.03**
SBP| deep_parietal~ age_scan+Sex	0.091	**0.013**	0.02	0.161	**0.03**
SBP| deep_occipital~ age_scan+Sex	0.011	0.774	-0.062	0.083	0.774
SBP| deep_temporal~ age_scan+Sex	0.035	0.351	-0.038	0.107	0.479
SBP| juxtacort_frontal~ age_scan+Sex	0.09	**0.014**	0.019	0.161	**0.03**
SBP| juxtacort_parietal~ age_scan+Sex	0.101	**0.006**	0.029	0.171	**0.03**
SBP| juxtacort_occipital~ age_scan+Sex	-0.021	0.572	-0.092	0.051	0.613
SBP| juxtacort_temporal~ age_scan+Sex	0.029	0.426	-0.042	0.1	0.533
DBP| periv_basal.ganglia~ age_scan+Sex	0.095	**0.01**	0.022	0.166	**0.03**

DBP| deep_basal.ganglia~ age_scan+Sex	0.03	0.407	-0.041	0.102	0.436
DBP| juxtacort_basal.ganglia~ age_scan+Sex	0.023	0.536	-0.049	0.095	0.536
DBP| periv_frontal~ age_scan+Sex	0.115	**0.002**	0.044	0.186	**0.01**
DBP| periv_parietal~ age_scan+Sex	0.066	0.069	-0.005	0.137	0.115
DBP| periv_occipital~ age_scan+Sex	0.106	**0.005**	0.033	0.178	**0.019**
DBP| periv_temporal~ age_scan+Sex	0.088	**0.015**	0.017	0.159	**0.03**
DBP| deep_frontal~ age_scan+Sex	0.09	**0.016**	0.017	0.162	**0.03**
DBP| deep_parietal~ age_scan+Sex	0.12	**0.001**	0.049	0.189	**0.01**
DBP| deep_occipital~ age_scan+Sex	0.056	0.132	-0.017	0.127	0.165
DBP| deep_temporal~ age_scan+Sex	0.065	0.077	-0.007	0.137	0.115
DBP| juxtacort_frontal~ age_scan+Sex	0.092	**0.012**	0.021	0.163	**0.03**
DBP| juxtacort_parietal~ age_scan+Sex	0.114	**0.002**	0.043	0.183	**0.01**
DBP| juxtacort_occipital~ age_scan+Sex	0.057	0.112	-0.013	0.127	0.153
DBP| juxtacort_temporal~ age_scan+Sex	0.053	0.156	-0.02	0.125	0.18
PP| periv_basal.ganglia~ age_scan+Sex	0.04	0.273	-0.032	0.112	0.409
PP| deep_basal.ganglia~ age_scan+Sex	0.077	**0.033**	0.006	0.148	0.307
PP| juxtacort_basal.ganglia~ age_scan+Sex	0.061	0.1	-0.012	0.133	0.375

PP| periv_frontal~ age_scan+Sex	0.046	0.206	-0.025	0.117	0.409
PP| periv_parietal~ age_scan+Sex	0.077	**0.041**	0.003	0.15	0.307
PP| periv_occipital~ age_scan+Sex	-0.024	0.506	-0.096	0.048	0.632
PP| periv_temporal~ age_scan+Sex	0.042	0.257	-0.031	0.115	0.409
PP| deep_frontal~ age_scan+Sex	0.06	0.098	-0.011	0.131	0.375
PP| deep_parietal~ age_scan+Sex	0.029	0.428	-0.043	0.101	0.584
PP| deep_occipital~ age_scan+Sex	-0.01	0.797	-0.082	0.063	0.854
PP| deep_temporal~ age_scan+Sex	0.019	0.611	-0.053	0.091	0.705
PP| juxtacort_frontal~ age_scan+Sex	0.051	0.161	-0.02	0.122	0.402
PP| juxtacort_parietal~ age_scan+Sex	0.052	0.154	-0.02	0.124	0.402
PP| juxtacort_occipital~ age_scan+Sex	-0.04	0.267	-0.111	0.031	0.409
PP| juxtacort_temporal~ age_scan+Sex	0.005	0.896	-0.066	0.076	0.896
RPP| periv_basal.ganglia~ age_scan+Sex	0.062	0.098	-0.012	0.135	0.163
RPP| deep_basal.ganglia~ age_scan+Sex	0.08	**0.031**	0.008	0.153	0.0664
RPP| juxtacort_basal.ganglia~ age_scan+Sex	0.035	0.332	-0.036	0.106	0.393
RPP| periv_frontal~ age_scan+Sex	0.131	**<0.001**	0.059	0.201	**<0.001**
RPP| periv_parietal~ age_scan+Sex	0.09	**0.014**	0.018	0.161	**0.035**

RPP| periv_occipital~ age_scan+Sex	0.03	0.393	-0.039	0.1	0.421
RPP| periv_temporal~ age_scan+Sex	0.059	0.118	-0.015	0.132	0.177
RPP| deep_frontal~ age_scan+Sex	0.117	**0.002**	0.044	0.188	**0.01**
RPP| deep_parietal~ age_scan+Sex	0.114	**0.002**	0.042	0.184	**0.01**
RPP| deep_occipital~ age_scan+Sex	0.039	0.275	-0.031	0.109	0.375
RPP| deep_temporal~ age_scan+Sex	0.034	0.341	-0.036	0.105	0.393
RPP| juxtacort_frontal~ age_scan+Sex	0.094	**0.01**	0.022	0.165	**0.03**
RPP| juxtacort_parietal~ age_scan+Sex	0.104	**0.005**	0.032	0.174	**0.019**
RPP| juxtacort_occipital~ age_scan+Sex	0.067	0.067	-0.005	0.138	0.126
RPP| juxtacort_temporal~ age_scan+Sex	0.024	0.51	-0.047	0.095	0.51
MAP| periv_basal.ganglia~ age_scan+Sex	0.111	**0.003**	0.038	0.182	**0.011**
MAP| deep_basal.ganglia~ age_scan+Sex	0.061	0.1	-0.012	0.133	0.15
MAP| juxtacort_basal.ganglia~ age_scan+Sex	0.049	0.179	-0.023	0.12	0.224
MAP| periv_frontal~ age_scan+Sex	0.126	**0.001**	0.055	0.195	**0.005**
MAP| periv_parietal~ age_scan+Sex	0.083	**0.023**	0.012	0.153	**0.043**
MAP| periv_occipital~ age_scan+Sex	0.071	0.054	-0.001	0.143	0.09
MAP| periv_temporal~ age_scan+Sex	0.089	**0.015**	0.018	0.16	**0.032**

MAP| deep_frontal~ age_scan+Sex	0.107	**0.004**	0.034	0.178	**0.012**
MAP| deep_parietal~ age_scan+Sex	0.118	**0.001**	0.047	0.187	**0.005**
MAP| deep_occipital~ age_scan+Sex	0.036	0.33	-0.036	0.108	0.354
MAP| deep_temporal~ age_scan+Sex	0.055	0.135	-0.017	0.127	0.184
MAP| juxtacort_frontal~ age_scan+Sex	0.098	**0.007**	0.027	0.168	**0.018**
MAP| juxtacort_parietal~ age_scan+Sex	0.117	**0.001**	0.046	0.186	**0.005**
MAP| juxtacort_occipital~ age_scan+Sex	0.025	0.479	-0.045	0.096	0.479
MAP| juxtacort_temporal~ age_scan+Sex	0.046	0.215	-0.027	0.117	0.248
HR| periv_basal.ganglia~ age_scan+Sex	0.022	0.544	-0.05	0.095	0.789
HR| deep_basal.ganglia~ age_scan+Sex	0.049	0.181	-0.023	0.12	0.388
HR| juxtacort_basal.ganglia~ age_scan+Sex	-0.015	0.684	-0.086	0.056	0.789
HR| periv_frontal~ age_scan+Sex	0.089	**0.015**	0.017	0.161	0.145
HR| periv_parietal~ age_scan+Sex	0.037	0.318	-0.035	0.108	0.596
HR| periv_occipital~ age_scan+Sex	0.006	0.864	-0.064	0.077	0.926
HR| periv_temporal~ age_scan+Sex	0.018	0.624	-0.055	0.092	0.789
HR| deep_frontal~ age_scan+Sex	0.087	**0.02**	0.014	0.159	0.145
HR| deep_parietal~ age_scan+Sex	0.078	**0.039**	0.004	0.151	0.146

HR| deep_occipital~ age_scan+Sex	0.025	0.492	-0.046	0.095	0.789
HR| deep_temporal~ age_scan+Sex	0.017	0.638	-0.055	0.089	0.789
HR| juxtacort_frontal~ age_scan+Sex	0.052	0.161	-0.021	0.124	0.388
HR| juxtacort_parietal~ age_scan+Sex	0.056	0.131	-0.017	0.128	0.388
HR| juxtacort_occipital~ age_scan+Sex	0.081	**0.029**	0.008	0.153	0.145
HR| juxtacort_temporal~ age_scan+Sex	-0.001	0.982	-0.073	0.072	0.982

Legend: CI (confidence interval); ρ *(Covariate-adjusted Spearman's correlation coefficient)*.

Supplementary Table 7. Results for the covariate-adjusted Spearman's rank correlation test assessing the association between blood pressure measurements and regional WMHV

Outcome	β	SE	P-value	Low CI	High CI	adjR2
SBP	0.045	0.034	0.190	-0.022	0.111	0.152
DBP	0.070	0.036	0.051	-0.001	0.140	0.061
HR	0.050	0.036	0.169	-0.021	0.121	0.046
MAP	0.067	0.035	0.058	-0.002	0.136	0.085
PP	0.003	0.033	0.939	-0.063	0.068	0.187
RPP	0.074	0.036	**0.038**	0.004	0.145	0.058

Supplementary Table 8. Results of the linear regression models assessing the association between the PRS of WMH and blood pressure measurements. *Footnote: the PRS of WMH and the hemodynamic measures were standardized. β* represents the increment in the blood pressure *measurements per one-standard deviation increase in the PRS of WMH.*

Outcome	Mediator	Effect	Estimate	P-value	Low CI	High CI
periv_frontal	DBP	ACME	0,008	**0,040**	0,000	0,019
periv_frontal	DBP	ADE	0,103	**<0,001**	0,055	0,179
periv_frontal	DBP	Total Effect	0,111	**<0,001**	0,061	0,186
periv_frontal	DBP	Proportion Mediated	0,069	**0,040**	0,002	0,223
periv_occipital	DBP	ACME	0,007	**0,040**	0,001	0,015
periv_occipital	DBP	ADE	0,085	**<0,001**	0,034	0,142
periv_occipital	DBP	Total Effect	0,091	**<0,001**	0,042	0,151
periv_occipital	DBP	Proportion Mediated	0,074	**0,040**	0,008	0,198
periv_temporal	DBP	ACME	0,007	0,080	-0,001	0,017
periv_temporal	DBP	ADE	0,072	**0,040**	0,014	0,124
periv_temporal	DBP	Total Effect	0,079	**0,040**	0,016	0,132
periv_temporal	DBP	Proportion Mediated	0,076	0,120	-0,034	0,262
deep_frontal	DBP	ACME	0,007	0,080	0,000	0,014
deep_frontal	DBP	ADE	0,080	**<0,001**	0,030	0,162
deep_frontal	DBP	Total Effect	0,087	**<0,001**	0,042	0,165
deep_frontal	DBP	Proportion Mediated	0,076	0,080	0,001	0,310
deep_parietal	DBP	ACME	0,008	**0,040**	0,000	0,018
deep_parietal	DBP	ADE	0,068	**0,040**	0,003	0,127
deep_parietal	DBP	Total Effect	0,076	**0,040**	0,007	0,138
deep_parietal	DBP	Proportion Mediated	0,099	0,080	-0,003	0,594
deep_occipital	DBP	ACME	0,005	0,240	-0,002	0,017

deep_occipital	DBP	ADE	0,059	0,080	-0,014	0,113
deep_occipital	DBP	Total Effect	0,064	0,120	-0,009	0,119
deep_occipital	DBP	Proportion Mediated	0,049	0,280	-0,241	0,353
deep_temporal	DBP	ACME	0,004	0,120	0,000	0,011
deep_temporal	DBP	ADE	0,110	**<0,001**	0,047	0,178
deep_temporal	DBP	Total Effect	0,114	**<0,001**	0,051	0,180
deep_temporal	DBP	Proportion Mediated	0,033	0,120	-0,002	0,095
juxtacort_frontal	DBP	ACME	0,008	**<0,001**	0,001	0,019
juxtacort_frontal	DBP	ADE	0,056	**0,040**	0,006	0,116
juxtacort_frontal	DBP	Total Effect	0,064	**0,040**	0,011	0,124
juxtacort_frontal	DBP	Proportion Mediated	0,101	**0,040**	0,008	0,467
periv_frontal	MAP	ACME	0,010	**<0,001**	0,002	0,022
periv_frontal	MAP	ADE	0,101	**<0,001**	0,047	0,169
periv_frontal	MAP	Total Effect	0,112	**<0,001**	0,056	0,172
periv_frontal	MAP	Proportion Mediated	0,095	**<0,001**	0,028	0,238
periv_occipital	MAP	ACME	0,006	**<0,001**	0,001	0,018
periv_occipital	MAP	ADE	0,084	**<0,001**	0,021	0,143
periv_occipital	MAP	Total Effect	0,091	**<0,001**	0,023	0,145
periv_occipital	MAP	Proportion Mediated	0,058	**<0,001**	0,009	0,198
periv_temporal	MAP	ACME	0,007	0,120	-0,001	0,015
periv_temporal	MAP	ADE	0,083	**<0,001**	0,026	0,149
periv_temporal	MAP	Total Effect	0,090	**<0,001**	0,028	0,156
periv_temporal	MAP	Proportion Mediated	0,082	0,120	-0,020	0,224

deep_frontal	MAP	ACME	0,009	0,120	-0,002	0,019
deep_frontal	MAP	ADE	0,078	**0,040**	0,010	0,136
deep_frontal	MAP	Total Effect	0,087	**0,040**	0,016	0,143
deep_frontal	MAP	Proportion Mediated	0,101	0,160	-0,081	0,301
deep_parietal	MAP	ACME	0,009	**0,040**	0,001	0,018
deep_parietal	MAP	ADE	0,073	**0,040**	0,016	0,147
deep_parietal	MAP	Total Effect	0,082	**0,040**	0,023	0,158
deep_parietal	MAP	Proportion Mediated	0,101	0,080	-0,016	0,319
deep_occipital	MAP	ACME	0,002	0,560	-0,003	0,008
deep_occipital	MAP	ADE	0,061	0,160	-0,012	0,121
deep_occipital	MAP	Total Effect	0,062	0,120	-0,009	0,126
deep_occipital	MAP	Proportion Mediated	0,024	0,600	-0,186	0,773
deep_temporal	MAP	ACME	0,005	0,120	0,000	0,013
deep_temporal	MAP	ADE	0,109	**<0,001**	0,038	0,161
deep_temporal	MAP	Total Effect	0,114	**<0,001**	0,045	0,165
deep_temporal	MAP	Proportion Mediated	0,044	0,120	-0,003	0,176
juxtacort_frontal	MAP	ACME	0,009	**0,040**	0,000	0,020
juxtacort_frontal	MAP	ADE	0,067	0,080	-0,004	0,136
juxtacort_frontal	MAP	Total Effect	0,076	**<0,001**	0,007	0,153
juxtacort_frontal	MAP	Proportion Mediated	0,105	**0,040**	0,009	1,760
periv_frontal	RPP	ACME	0,009	0,080	-0,002	0,021
periv_frontal	RPP	ADE	0,110	**<0,001**	0,026	0,172
periv_frontal	RPP	Total Effect	0,119	**<0,001**	0,031	0,180
periv_frontal	RPP	Proportion	0,067	0,080	-0,010	0,213

		Mediated				
periv_occipital	RPP	ACME	0,002	0,440	-0,003	0,009
periv_occipital	RPP	ADE	0,082	**<0,001**	0,010	0,136
periv_occipital	RPP	Total Effect	0,084	**<0,001**	0,015	0,140
periv_occipital	RPP	Proportion Mediated	0,028	0,440	-0,033	0,171
periv_temporal	RPP	ACME	0,005	**0,040**	0,001	0,010
periv_temporal	RPP	ADE	0,084	**0,040**	0,015	0,144
periv_temporal	RPP	Total Effect	0,090	**0,040**	0,020	0,150
periv_temporal	RPP	Proportion Mediated	0,062	0,080	0,000	0,178
deep_frontal	RPP	ACME	0,009	**0,040**	0,001	0,021
deep_frontal	RPP	ADE	0,077	**0,040**	0,002	0,158
deep_frontal	RPP	Total Effect	0,086	**0,040**	0,012	0,173
deep_frontal	RPP	Proportion Mediated	0,093	0,080	-0,031	0,462
deep_parietal	RPP	ACME	0,009	**<0,001**	0,001	0,020
deep_parietal	RPP	ADE	0,077	**0,040**	0,011	0,139
deep_parietal	RPP	Total Effect	0,086	**<0,001**	0,020	0,146
deep_parietal	RPP	Proportion Mediated	0,113	**<0,001**	0,016	0,517
deep_occipital	RPP	ACME	0,001	0,640	-0,006	0,006
deep_occipital	RPP	ADE	0,054	0,120	-0,013	0,130
deep_occipital	RPP	Total Effect	0,054	0,120	-0,017	0,133
deep_occipital	RPP	Proportion Mediated	0,018	0,680	-1,072	0,212
deep_temporal	RPP	ACME	0,004	0,200	-0,001	0,012
deep_temporal	RPP	ADE	0,087	**0,040**	0,010	0,153
deep_temporal	RPP	Total Effect	0,091	**0,040**	0,016	0,159

deep_temporal	RPP	Proportion Mediated	0,038	0,160	-0,011	0,223
juxtacort_frontal	RPP	ACME	0,008	0,080	0,000	0,017
juxtacort_frontal	RPP	ADE	0,073	0,080	-0,003	0,127
juxtacort_frontal	RPP	Total Effect	0,081	0,080	0,001	0,135
juxtacort_frontal	RPP	Proportion Mediated	0,100	0,160	-0,182	0,345

Supplementary Table 9. Results for the mediation analysis exploring the mediator role of DBP. RPP and MAP in the association between the PRS of WMH and regional WMHV.
Legend: CI (confidence interval); ACME (Average Causal Mediation Effect) ; ADE (Average Direct Effect)

Outcome	Mediator	Effect	Estimate	P-value	Low CI	High CI
periv_frontal	DBP	ACME	0,007	0,240	-0,001	0,019
periv_frontal	DBP	ADE	0,115	**<0,001**	0,054	0,177
periv_frontal	DBP	Total Effect	0,122	**<0,001**	0,057	0,179
periv_frontal	DBP	Proportion Mediated	0,048	0,240	-0,015	0,149
periv_occipital	DBP	ACME	0,005	0,080	-0,001	0,013
periv_occipital	DBP	ADE	0,091	**<0,001**	0,028	0,166
periv_occipital	DBP	Total Effect	0,096	**<0,001**	0,032	0,168
periv_occipital	DBP	Proportion Mediated	0,050	0,080	-0,007	0,164
periv_temporal	DBP	ACME	0,006	**0,040**	0,000	0,013
periv_temporal	DBP	ADE	0,077	**<0,001**	0,014	0,135
periv_temporal	DBP	Total Effect	0,082	**<0,001**	0,018	0,138
periv_temporal	DBP	Proportion Mediated	0,061	**0,040**	0,006	0,246
deep_frontal	DBP	ACME	0,006	0,120	0,000	0,015
deep_frontal	DBP	ADE	0,093	**<0,001**	0,021	0,164
deep_frontal	DBP	Total Effect	0,099	**<0,001**	0,035	0,174
deep_frontal	DBP	Proportion Mediated	0,048	0,120	-0,003	0,233
deep_parietal	DBP	ACME	0,009	**0,040**	0,002	0,021
deep_parietal	DBP	ADE	0,089	**<0,001**	0,023	0,169
deep_parietal	DBP	Total Effect	0,098	**<0,001**	0,033	0,173
deep_parietal	DBP	Proportion Mediated	0,087	**0,040**	0,011	0,352
deep_occipital	DBP	ACME	0,003	0,600	-0,005	0,010

deep_occipital	DBP	ADE	0,050	0,240	-0,009	0,148
deep_occipital	DBP	Total Effect	0,053	0,120	-0,004	0,152
deep_occipital	DBP	Proportion Mediated	0,045	0,640	-0,242	1,675
deep_temporal	DBP	ACME	0,003	0,240	-0,003	0,010
deep_temporal	DBP	ADE	0,123	**<0,001**	0,035	0,181
deep_temporal	DBP	Total Effect	0,126	**<0,001**	0,042	0,185
deep_temporal	DBP	Proportion Mediated	0,024	0,240	-0,054	0,138
juxtacort_frontal	DBP	ACME	0,008	0,080	0,000	0,016
juxtacort_frontal	DBP	ADE	0,068	0,120	-0,006	0,141
juxtacort_frontal	DBP	Total Effect	0,076	0,080	0,000	0,155
juxtacort_frontal	DBP	Proportion Mediated	0,081	0,160	-0,041	0,398
periv_frontal	MAP	ACME	0,007	0,120	-0,001	0,018
periv_frontal	MAP	ADE	0,118	**<0,001**	0,040	0,221
periv_frontal	MAP	Total Effect	0,125	**<0,001**	0,047	0,227
periv_frontal	MAP	Proportion Mediated	0,051	0,120	-0,005	0,185
periv_occipital	MAP	ACME	0,003	0,240	-0,004	0,014
periv_occipital	MAP	ADE	0,097	**<0,001**	0,037	0,161
periv_occipital	MAP	Total Effect	0,101	**<0,001**	0,040	0,162
periv_occipital	MAP	Proportion Mediated	0,029	0,240	-0,036	0,119
periv_temporal	MAP	ACME	0,005	0,120	-0,002	0,014
periv_temporal	MAP	ADE	0,086	**<0,001**	0,007	0,144
periv_temporal	MAP	Total Effect	0,091	**<0,001**	0,019	0,147
periv_temporal	MAP	Proportion Mediated	0,047	0,120	-0,016	0,433

deep_frontal	MAP	ACME	0,008	**<0,001**	0,001	0,018
deep_frontal	MAP	ADE	0,087	**0,040**	0,018	0,149
deep_frontal	MAP	Total Effect	0,095	**<0,001**	0,028	0,153
deep_frontal	MAP	Proportion Mediated	0,071	**<0,001**	0,007	0,426
deep_parietal	MAP	ACME	0,010	**<0,001**	0,001	0,020
deep_parietal	MAP	ADE	0,090	**<0,001**	0,032	0,155
deep_parietal	MAP	Total Effect	0,100	**<0,001**	0,047	0,175
deep_parietal	MAP	Proportion Mediated	0,092	**<0,001**	0,010	0,320
deep_occipital	MAP	ACME	0,000	0,840	-0,005	0,005
deep_occipital	MAP	ADE	0,059	0,200	-0,012	0,130
deep_occipital	MAP	Total Effect	0,059	0,160	-0,013	0,132
deep_occipital	MAP	Proportion Mediated	-0,002	0,920	-0,193	0,317
deep_temporal	MAP	ACME	0,002	0,520	-0,003	0,009
deep_temporal	MAP	ADE	0,121	**<0,001**	0,060	0,215
deep_temporal	MAP	Total Effect	0,123	**<0,001**	0,065	0,219
deep_temporal	MAP	Proportion Mediated	0,010	0,520	-0,026	0,067
juxtacort_frontal	MAP	ACME	0,007	**<0,001**	0,000	0,018
juxtacort_frontal	MAP	ADE	0,062	0,120	-0,033	0,124
juxtacort_frontal	MAP	Total Effect	0,069	0,120	-0,021	0,129
juxtacort_frontal	MAP	Proportion Mediated	0,084	0,120	-0,414	0,327
periv_frontal	RPP	ACME	0,010	**<0,001**	0,001	0,021
periv_frontal	RPP	ADE	0,115	**<0,001**	0,057	0,197
periv_frontal	RPP	Total Effect	0,125	**<0,001**	0,058	0,204
periv_frontal	RPP	Proportion	0,082	**<0,001**	0,009	0,198

		Mediated				
periv_occipital	RPP	ACME	0,001	0,640	-0,008	0,008
periv_occipital	RPP	ADE	0,093	**<0,001**	0,042	0,156
periv_occipital	RPP	Total Effect	0,094	**<0,001**	0,042	0,158
periv_occipital	RPP	Proportion Mediated	0,012	0,640	-0,102	0,142
periv_temporal	RPP	ACME	0,003	0,520	-0,006	0,017
periv_temporal	RPP	ADE	0,073	**<0,001**	0,010	0,149
periv_temporal	RPP	Total Effect	0,076	**<0,001**	0,009	0,152
periv_temporal	RPP	Proportion Mediated	0,037	0,520	-0,251	0,458
deep_frontal	RPP	ACME	0,010	**<0,001**	0,002	0,021
deep_frontal	RPP	ADE	0,089	**<0,001**	0,026	0,161
deep_frontal	RPP	Total Effect	0,100	**<0,001**	0,034	0,174
deep_frontal	RPP	Proportion Mediated	0,096	**<0,001**	0,022	0,264
deep_parietal	RPP	ACME	0,009	**<0,001**	0,001	0,019
deep_parietal	RPP	ADE	0,089	**0,040**	0,016	0,148
deep_parietal	RPP	Total Effect	0,098	**<0,001**	0,029	0,150
deep_parietal	RPP	Proportion Mediated	0,076	**<0,001**	0,013	0,461
deep_occipital	RPP	ACME	0,001	0,800	-0,006	0,008
deep_occipital	RPP	ADE	0,063	0,120	-0,011	0,155
deep_occipital	RPP	Total Effect	0,064	0,120	-0,011	0,157
deep_occipital	RPP	Proportion Mediated	0,003	0,840	-0,438	0,466
deep_temporal	RPP	ACME	0,002	0,480	-0,006	0,009
deep_temporal	RPP	ADE	0,119	**<0,001**	0,039	0,188
deep_temporal	RPP	Total Effect	0,121	**<0,001**	0,043	0,188

deep_temporal	RPP	Proportion Mediated	0,014	0,480	-0,046	0,105
juxtacort_frontal	RPP	ACME	0,008	**<0,001**	0,002	0,017
juxtacort_frontal	RPP	ADE	0,060	0,200	-0,007	0,140
juxtacort_frontal	RPP	Total Effect	0,069	0,080	-0,001	0,145
juxtacort_frontal	RPP	Proportion Mediated	0,128	0,080	-0,490	1,200

Supplementary Table 10. Results for the mediation analysis exploring the mediator role of DBP. RPP and MAP in the association between the PRS of WMH and regional WMHV in non-hypertensive individuals. *Legend: CI (confidence interval); ACME (Average Causal Mediation Effect) ; ADE (Average Direct Effect)*
